# Comparative Pathogenomics of *Escherichia coli*: Polyvalent Vaccine Target Identification through Virulome Analysis

**DOI:** 10.1128/IAI.00115-21

**Published:** 2021-07-15

**Authors:** J. R. Clark, A. M. Maresso

**Affiliations:** a BCM Tailor Labs, Department of Molecular Virology and Microbiology, Baylor College of Medicine, Houston, Texas, USA; Stanford University

**Keywords:** *Escherichia coli*, vaccine development, comparative genomics, pathogenomics, ExPEC, InPEC, enteric pathogens, genomics, pathogenesis, vaccines, virulence factors

## Abstract

Comparative genomics of bacterial pathogens has been useful for revealing potential virulence factors. Escherichia coli is a significant cause of human morbidity and mortality worldwide but can also exist as a commensal in the human gastrointestinal tract. With many sequenced genomes, it has served as a model organism for comparative genomic studies to understand the link between genetic content and potential for virulence. To date, however, no comprehensive analysis of its complete “virulome” has been performed for the purpose of identifying universal or pathotype-specific targets for vaccine development. Here, we describe the construction of a pathotype database of 107 well-characterized completely sequenced pathogenic and nonpathogenic E. coli strains, which we annotated for major virulence factors (VFs). The data are cross referenced for patterns against pathotype, phylogroup, and sequence type, and the results were verified against all 1,348 complete E. coli chromosomes in the NCBI RefSeq database. Our results demonstrate that phylogroup drives many of the “pathotype-associated” VFs, and ExPEC-associated VFs are found predominantly within the B2/D/F/G phylogenetic clade, suggesting that these phylogroups are better adapted to infect human hosts. Finally, we used this information to propose polyvalent vaccine targets with specificity toward extraintestinal strains, targeting key invasive strategies, including immune evasion (group 2 capsule), iron acquisition (FyuA, IutA, and Sit), adherence (SinH, Afa, Pap, Sfa, and Iha), and toxins (Usp, Sat, Vat, Cdt, Cnf1, and HlyA). While many of these targets have been proposed before, this work is the first to examine their pathotype and phylogroup distribution and how they may be targeted together to prevent disease.

## INTRODUCTION

Escherichia coli has long been a significant cause of human morbidity and mortality. It is a common inhabitant of the gastrointestinal tract, readily exchanges elements associated with antibiotic resistance, and has an extensive pangenome of at least 13,000 genes (and possibly over 100,000) which include numerous virulence factors (VFs) that complicate an understanding of disease etiology (1–12). Currently, there is no vaccine available, and attempts to generate one have been limited by the lack of universal protection against all invasive strains or types of infection this organism is capable of causing (13–15). Unlike the causative agent of diphtheria and pertussis, which are largely viewed as toxigenic diseases caused by secreted toxins from Corynebacterium diph theriae and Bordetella pertussis, respectively, E. coli infections largely resist being pigeon-holed by a single VF, instead being multifactorial—a composite of virulence factors contributing to each step in the diverse range of diseases this organism causes (11, 16–20). This and the fact that the organism borders on being an opportunistic pathogen—with commensal strains often apparently possessing the same virulence factors—makes the development of vaccines challenging (21). Along these lines, strains are organized into so-called pathological types or “pathotypes” (also known as pathovars). Pathotypes are groups of pathogenic strains that share the same phenotype of disease. In a broad sense, these can be broken up into two types based on whether the strain causes disease outside (extraintestinal pathogenic E. coli [ExPEC]) or inside (intestinal pathogenic E. coli [InPEC]) the intestines (12, 22). These two pathotypes are broken down further based on the specifics of the disease they cause. For example, neonatal meningitis E. coli (NMEC) and uropathogenic E. coli (UPEC) are considered ExPECs, while enteroaggregative E. coli (EAEC) and enterohemorrhagic E. coli (EHEC) are InPECs. A full list of the pathotypes discussed here, along with their acronyms and characteristics, can be found in Table 1.

**TABLE 1:** E. coli pathotypes, acronyms, disease presentations, and associated VFs

Pathotype	Acronym	Disease presentation(s)	Associated VFs (references)
Extraintestinal Pathogenic E. coli	ExPEC	Disease outside the intestines	See below
Uropathogenic E. coli	UPEC	Urinary tract infections (23, 24)	*fim*, *hlyA*, *csg*, *pap*, *sfa*, *afa*, *cdtAB*, *iha*, *iutA*, *iroN*, *fyuA*, *sitA*, *chuA*, *hma*, *kpsMT*, *agn43/flu*, *pic*, *sat*, *vat*, *usp* (12, 24, 25)
Neonatal Meningitis E. coli	NMEC	Bacterial meningitis	*fim*, *sfa*, *mat*, *ibeA*, *irp*, *iroN*, *kpsMT*, K1 (25)
Avian Pathogenic E. coli	APEC	Multiple ExPEC diseases in avian species (26–28)	*Colicin*, *ibeA*, *iutA*, *iroN*, *sitA*, *tsh*, *fim*, *fyuA*, *pap*, *vat* (12, 25, 28)
Intestinal Pathogenic E. coli	InPEC	Disease of the intestines	See below
Adherent-Invasive E. coli	AIEC	Associated with intestinal inflammation (29, 30)	*fimH*, *lpf*, *pap*, *sfa*, *afa*, *vat*, *hlyA*, *cnf I*, *cdtAB*, *ibeA* (31–33)
Enterohemorrhagic E. coli	EHEC	Bloody diarrhea, hemorrhagic colitis, hemolytic-uremic syndrome (32)	LEE pathogenicity island; *stx*, *espP*, *lpf*, *efa*, *toxB*, *eibG*, *ehaA*, *ompA*, *iha*, *paa* (32, 34)
Enteroaggregative E. coli	EAEC	Acute and chronic watery diarrhea (32, 35)	*set*, *agg*, *aaf*, *agg3*, *astA*, *pet*, *sat*, *aap*, *aagR*, *shf*, *pic*, *irp2*, *hly*, *tia* (32, 36)
Enteroaggregative Hemorrhagic E. coli	EAHEC	Similar to EHEC, with increased adherence and antibiotic resistance (32, 37)	*iha*, *pic*, *pet*, *stx* (32)
Enterotoxigenic E. coli	ETEC	Mild to severe watery diarrhea (32)	*CFA* fimbriae, *astA*, *eltAB*, *estIa*, *clyA*, *eatA* (32, 38)
Enteropathogenic E. coli	EPEC	Severe acute watery diarrhea (32, 39, 40)	LEE pathogenicity island, *set*, *paa*, *lpf*, *iha*, *ehaA* (32, 41)

Strains are also characterized by the phylogenetic group (phylogroup) to which they belong. In contrast to pathotype, which are based largely on disease phenotype, phylogroup assignments are based on genetic lineage. There are four major (A, B1, B2, and D) and five minor (C, E, F, G, and cryptic clade I) phylogroups in E. coli (42). The four major phylogroups were first observed by multilocus enzyme electrophoresis and subsequently found to be resolved by grouping core E. coli genomes (43, 44). One main, if underappreciated, difference between phylogroups is the presence of the *chu* system responsible for heme importation. This system is found in phylogroup B2, D, E, F, and G strains but is absent in phylogroup A, B1, and C strains. Iron is an essential, possibly growth rate-limiting, nutrient that is found predominantly in heme of red blood cells in mammalian hosts (45–47). Phylogroups A and B1 appear to be the source of most human commensals (44, 48). Strains of the B1 phylogroup also may be more prevalent in domesticated animal isolates (44). In contrast to the A and B1 phylogroups, strains from the B2 and D phylogroups are more commonly found in ExPEC strains (48–50). Less is known about the minor phylogroups, but ST23 and ST88 of phylogroup C are often found to be avian pathogenic E. coli (APEC) strains (51–53), while phylogroup E contains most of the known EHEC isolates, such as the well-known O157:H7 serotype. Phylogroup F is very similar to phylogroup B2, but with the exception of certain sequence types, they generally lack many of the ExPEC-associated virulence factors (11, 54). Lastly, phylogroup G has only recently been resolved, and little is known about it other than being found between phylogroups F and B2 phylogenetically (55).

Here, we use a comparative genomics approach to comprehensively organize every major virulence factor in E. coli into a holistic picture that allows comparison across pathotype, phylogroup, and VF category. This organization reveals vaccine targets that speciate by phylogroup specifically but also highlight some unexpected entry points that may disarm this pathogen from multiple angles. Many of the vaccine targets proposed here have been proposed before as monovalent targets. However, to our knowledge, there has been no work looking at their pathotype and phylogroup distribution or how this could be leveraged to identify novel vaccine targets or polyvalent strategies. Furthermore, we reveal here several novel findings that provide insight into E. coli evolution, pathogen-versus-commensal delineation, and diagnostic classification.

## RESULTS AND DISCUSSION

### Curation of the *E. coli* virulome and visualization of results.

The first step in our analysis was to curate a database of E. coli virulence factors by known strains. This included retrieving known virulence factors from the Virulence Factor Database (VFDB), VICTORs, and PATRIC, cross-referencing and confirming their function from hundreds of literature sources, and using a tiered approach to analyze strains (56–58). On the first tier, we used a strain database of 107 strains which had complete chromosome sequences (sequences of high quality) and had published evidence for their pathotype assignment. Two incomplete strains were grandfathered in from preliminary analyses (NC101 and REL606), but these nonetheless had published pathotype evidence. These strains were organized by their pathotype and visualized in detail. This is referred to here as our “pathotype database.” The next tier database contained 1,348 complete E. coli chromosomes that were organized into phylogroups using an *in silico* method based on Clermont phylotyping developed in-house (see Materials and Methods; see also Appendix S1 in the supplemental material) (59–61). This is referred to as our “phylogroup database.” Genetic insights gleaned from the first tier (pathotype database) were tested against this larger phylogroup database, mainly in the form of gene distribution. Using both methods, we found many apparently novel associations between virulence factors, phylogroups, and pathotypes. Figure S1 details the pipeline for separation of the E. coli genomes into phylogroups.

**FIG 1:**
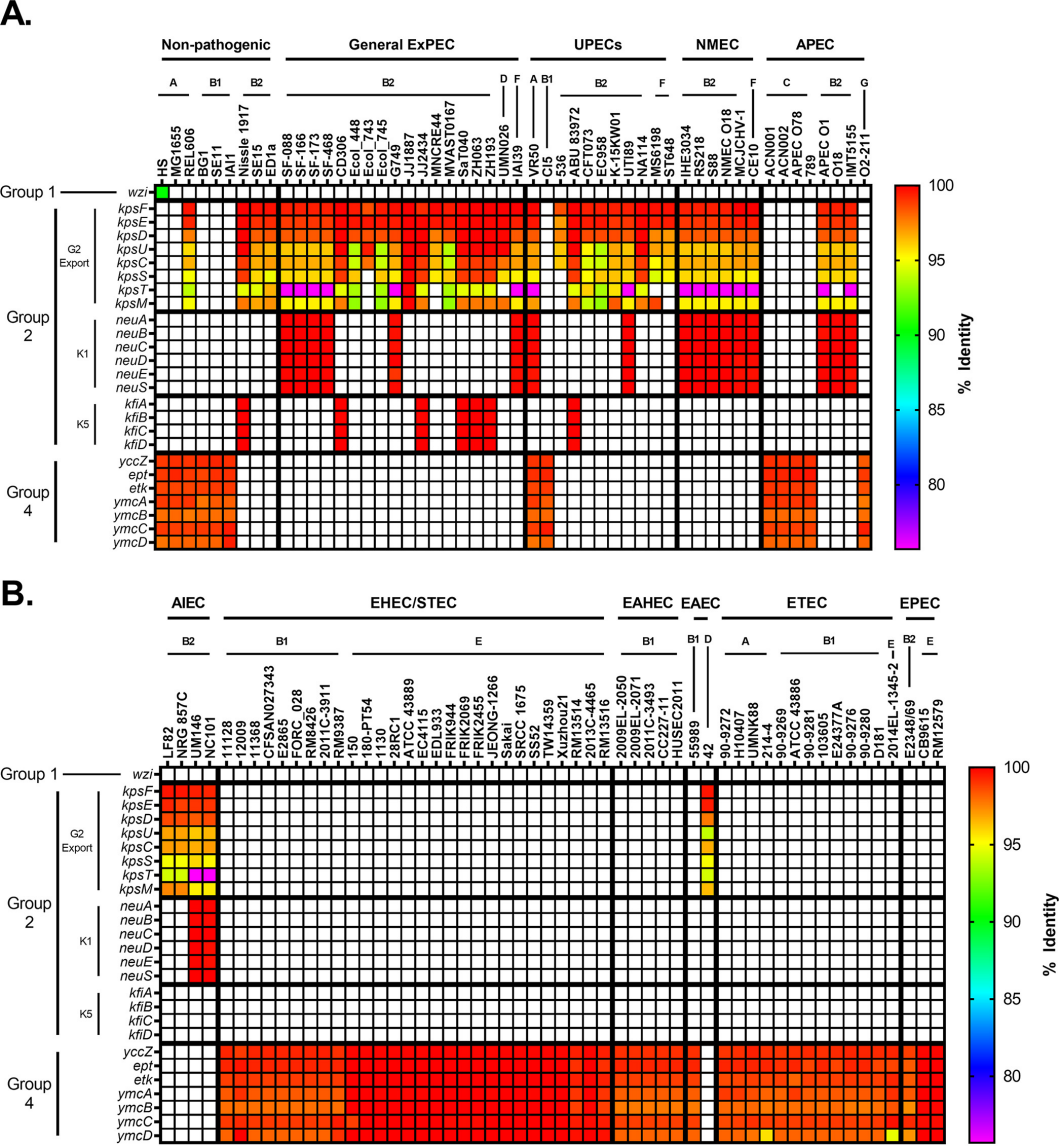
Pathotype distribution of E. coli capsule. (A and B) Heatmap showing nonpathogenic E. coli and ExPECs (A) and InPECs (B). Columns are organized first by pathotype, then by phylogroup, and finally by sequence type (sequence type not shown). The *wzi* gene used as a reference is specific for G1C. G2C and G3C both use the *kpsFEDUCS* and *kpsTM* operons for export. The biosynthetic operons for the most widely distributed and studied K-types, K1 (*neu*) and K5 (*kfi*), are also shown. G4C is synthesized by *yccZ*, *ept*, *etk*, and *ymcABCD* genes. The percent identity was determined using megaBLAST with reference genes found in Data Set S1 in the supplemental material.

To visualize the relationship between pathotype, phylogroup, type of virulence factor, and any polymorphisms in genes associated with virulence factors, a heatmap template was developed. These heatmaps are divided into two panels: nonpathogenic strains and ExPECs (general ExPECs, UPECs, NMECs, and APECs) (see, for example, Fig. 1A) and InPECs (AIECs, EHECs/STECs, EAHECs, EAECs, ETECs, and EPECs [Table 1]) (see, for example, Fig. 1B). Each column represents a single strain that is listed at the top of the heatmap. These strains are organized first by pathotype, then by phylogroup, and finally by sequence type. Rows represent a single gene, which is listed to the left of the heatmap. Genes are generally organized by class, operon, or otherwise related function.

Each cell in the heatmap is colored based on percent nucleotide identity compared to reference used to generate the alignments. The range of colors for each figure are based on the lowest value—which indicates divergence from the reference gene—and so varies from figure to figure. Coloring cells this way allows the user to use color as a proxy for both conservation and allelic distribution, and our organization allows the reader to investigate this distribution by pathotype, phylogroup, or sequence type. It is important to note that matching colors do not necessarily mean identical alleles. Instead, it indicates that the gene in those strains have the same number of mutations relative to the reference gene. However, we did not come across a case where matching colors were from separate alleles.

### K-capsule group types are strongly associated with phylogroup.

Surface sugars are an important virulence factor in many pathogenic bacteria as they generally act as protectins, hiding the bacterium from the host’s immune system (62). Three U.S. Food and Drug Administration-approved vaccines (for N. meningitidis, H. influenzae, and S. pneumoniae) have capsule as their component and thus may guide E. coli vaccine development since E. coli also produces capsule. In E. coli there are three major types of surface polysaccharides: lipopolysaccharide (LPS), capsular polysaccharide (CPS or K-antigen), and exopolysaccharide (EPS) (63–66). Of these, LPS and CPS are serospecific surface polysaccharides (64, 66). E. coli K-antigens are characterized into groups 1, 2, 3, and 4 (64). K-antigens that belong to groups 1 (G1C) and 4 (G4C) are related to LPS O-antigens and use similar biosynthetic machinery. G1C and G4C are found in intestinal pathogenic E. coli, including EPECs, ETECs, and EHECs (64). Group 2 capsule (G2C) and 3 (G3C) are found in extraintestinal pathogenic E. coli and are the group of interest here (64). K-antigens from these groups utilize a separate assembly and transport system (the *kps* operon) to those of the group 1 and 4 K-types. The structure of G2C and G3C are similar to those found in N. meningitidis and H. influenzae bacteria, which strengthens the case for using them as vaccine targets. G2C often appear similar to polysaccharides found on the surface of eukaryotic cells, and these include two of the most extensively studied K-antigens, K1 and K5 (64).

G1C was found only in one of the strains examined in our pathotype database, the commensal strain HS (nonpathogenic; A) (Fig. 1). G3C was found in none of the strains. While initially puzzling, the G1C and G3C distribution in our RefSeq phylogroup databases indicate that G1C and G3C are not widespread and found predominantly in phylogroups C and D, respectively. These two phylogroups are underrepresented in our pathotype database (Fig. 1). Given the rarity of G1C and G3C in both databases, they are not recommended candidates for vaccine development.

**FIG 2:**
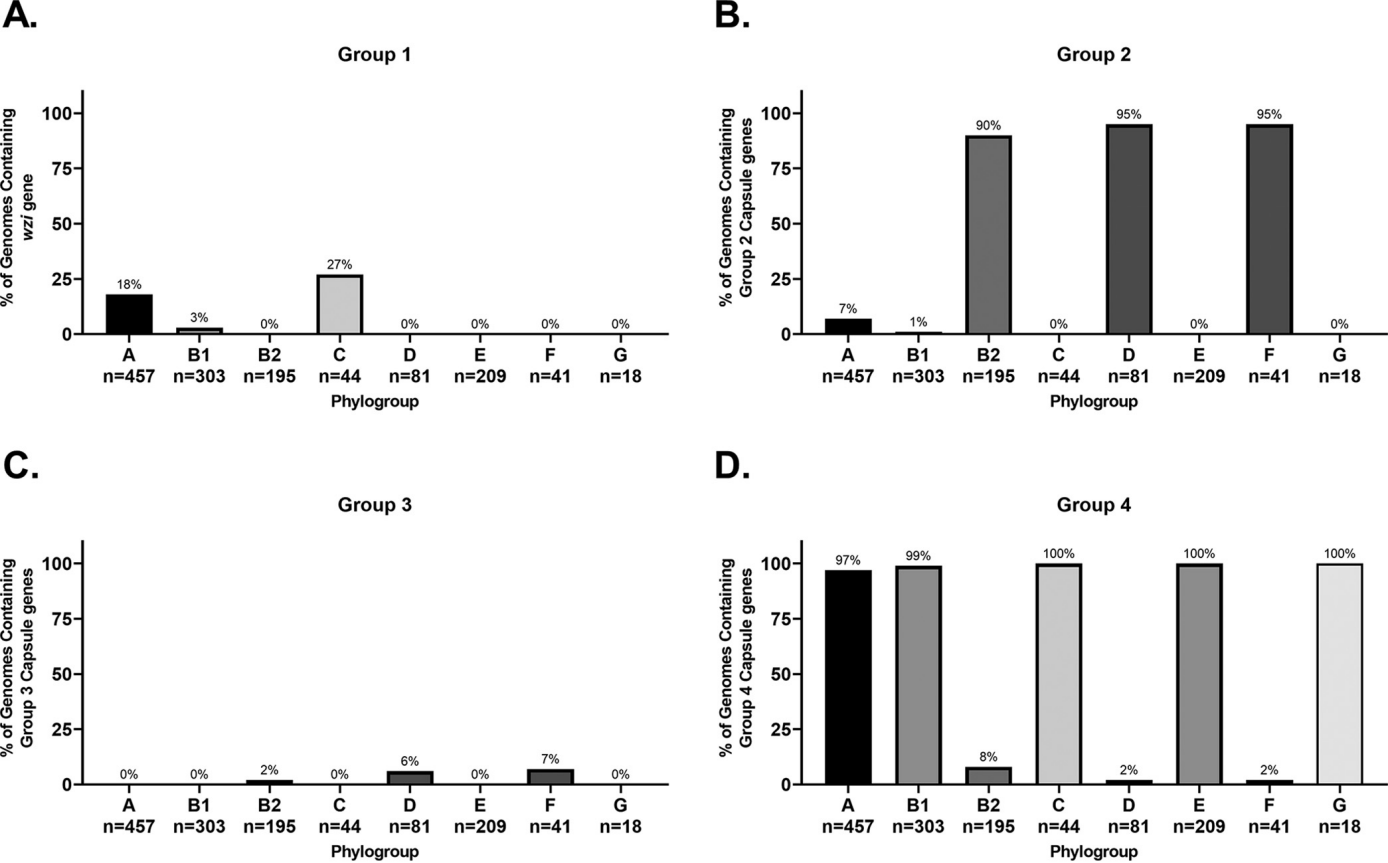
Phylogroup distribution of E. coli capsule. megaBLAST was used to bin strains of each phylogroup based on hit versus no hit. (A) Distribution of *wzi* from strain HS, which is specific for G1C. (B) Distribution of *kpsMTII*. (C) Distribution of *kpsMIII* (using AAC38078.1 as a reference). (D) Distribution of *ymcDCBA*, *yccZ*, *ept*, and *etk*, which are specific for G4C. Distributions were determined using megaBLAST to bin strains from each phylogroup into hit versus no hit.

G2C and G4C were more common, being found mostly in ExPECs and InPECs of our pathotype database, respectively (Fig. 1). These results suggested that G2C are by far the most common capsule type found in ExPECs and are almost completely absent from InPECs, with the exception of E. coli 042 (EAEC), a member of the D phylogroup. This trend agreed with the literature that G2C are virulence factors of ExPECs, and it further strengthens the notion that capsular polysaccharide from group 2 should be considered a vaccine target. The G2C distribution in our RefSeq phylogroup database showed that G2C is associated with the B2, D, and F phylogroups, rather than with the ExPEC pathotype, which explains G2C in the 042 (EAEC; D) strain (Fig. 1B). However, this strong phylogroup association does not exclude a pathotype-based selection. Instead, this may partly explain why ExPEC strains tend to be from these three phylogroups. This is highlighted by exceptions to the rule in strain VR50, an asymptomatic bacteriuria (ABU) strain of the A phylogroup, which contains both G2C and G4C, and the E2348/69 strain, which is an EPEC strain and the only member of the B2 phylogroup in our pathotype database that lacks G2C and contains G4C capsule (Fig. 1). In our phylogroup database, only 8% (15/195) of B2 strains carried the genes to produce G4C (Fig. 2). Of these, 87% (13/15) also carried the locus of enterocyte effacement (LEE) which is a pathogenicity island associated with diarrheal E. coli (namely, EHECs and EPECs) (67). Of B2 strains not carrying genes to encode the G4C, none of these 180 strains carry the LEE pathogenicity island. This, as well as the overall pathotype distribution of G2C and G4C, suggests that G4C may be important for survival within the intestines, while G2C is important for survival in other parts of the host. Thus, it would seem that targeting G2C capsule might be protective against strains that disseminate from the gastrointestinal tract into extraintestinal tissues. This has interesting implications since the phylogroup distribution of these capsule groups suggests that the B2, D, and F phylogroups are adapted to infections outside the intestines and opens up the range of potential vaccine targets from known virulence factors and surface antigens to those that are associated specifically with these phylogroups.

Interestingly, all AIEC strains, which are found in the intestines, also carried G2C (Fig. 1B) (68). This appears to be because the AIEC strains in this data set are from the B2 phylogroup; however, the majority of AIEC strains isolated thus far belong to the B2 phylogroup, so that does not necessarily mean our strains are not representative. This deviation from the phylogroups of other InPECs may be explained by the association of AIECs with inflammatory bowel disease. In such a dysregulated and immune-factor-heavy environment, a capsule type that is associated with avoidance of the immune system provides obvious benefits. This dovetails with our findings above: if G2C acts as a protectin against the immune system outside the intestines and G2C is predominantly found in B2 strains, this could explain why most AIEC strains belong to the B2 phylogroup—they simply are more resistant to the immune system and possibly survive better intracellularly (69–72).

Lastly, another interesting finding is that strains from the recently described phylogroup G, which is a sister phylogroup to the B2 phylogroup, apparently carry G4C (Fig. 2) (55). This may suggest that phylogroup G, like B2 EPEC strains, diverged from phylogroups B2, D, and F and is more adapted to an intestinal niche. This also has interesting implications for the evolution and acquisition of capsular genes, since it suggests that these divergent G and B2 strains may have exchanged G2C for G4C, which has been an important step in their evolutionary development.

### *kpsT* is predictive for group 2 capsule K-types.

**FIG 3:**
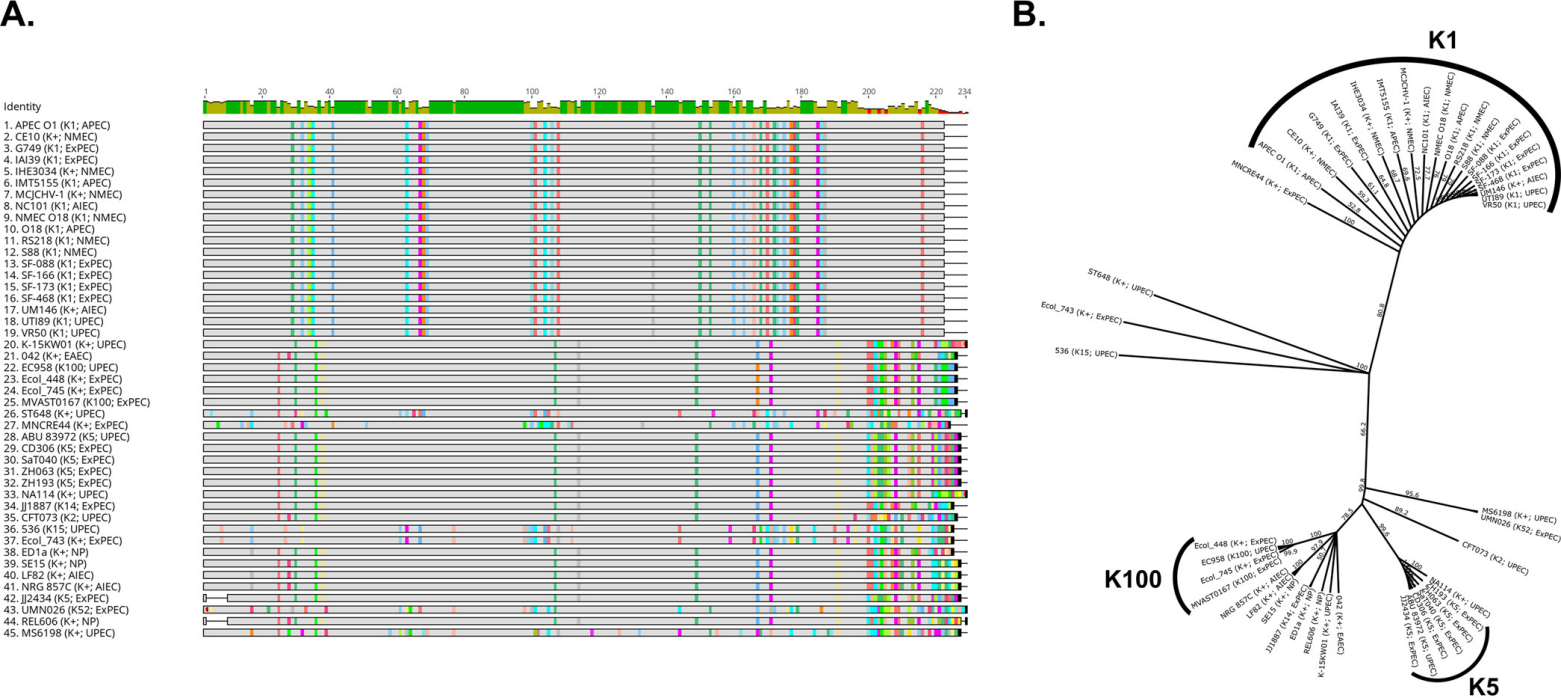
Alignment of KpsT shows strong association between *kpsT* allele and K-antigen. (A) Geneious alignment of the amino acid sequence of KpsT. Sequences were sorted by differences to compared to K1 strain KpsT. An identity histogram is shown at the top, and colors represent amino acid differences from the majority consensus. (B) Unrooted phylogenetic tree built from using Geneious TreeMaker and the alignment shown in panel A, with bootstrap support with 1,000 replicates. Branch labels indicate percent consensus support. Branches were transformed proportionally to allow for clearer comparison.

One of the most exciting things about the work presented here is the novel patterns that emerge from this layout. A good example of this was a striking result from the capsule comparisons where there was an apparent 1:1 association of the K-type with specific alleles of *kpsT*, a gene which encodes a ATP-binding protein member of the KpsTM ABC transport complex (Fig. 1; see also Fig. S2 in the supplemental material). While other genes in the *kps* operon showed either significant differences within each K-type or high conservation across K-types, *kpsT* showed a high degree of difference between K-types, but little-to-no change within a given K-type (Fig. 3; see also Fig. S3 in the supplemental material). This can clearly be seen in the amino acid alignment of all the KpsT sequences in our pathotype database (Fig. 3A). To test whether this trend holds up in a larger data set, we took advantage of the fact that the biosynthetic operons for two well-known G2C K-types are known: *neu* for K1 and *kfi* for K5. We then used megaBLAST to search our phylogroup database for 100% identical matches for the *kpsT* allele found in strains known to have K1 (*kpsT_K1_*) or K5 (*kpsT_K5_*) and cross referenced them against strains that contained the *neu* or *kfi* operons, respectively. Overall, our results showed that 89% (58/65) of all strains carrying the *neu* operon also contain *kpsT_K1_*, while 94% of all strains carrying the *kfi* operon contain *kpsT_K5_*. However, if we lower the stringency and include *kpsT* alleles that were >99% identical (<5 single nucleotide polymorphisms) to *kpsT_K1_* or *kpsT_K5_*, 99% (64/65) of strains carrying the *neu* operon carry a *kpsT_K1_*-like allele, and 100% (50/50) of strains carrying the *kfi* operon carry a *kpsT_K5_*-like allele. These results show that *kpsT* is tightly linked to K-type and suggest that it may interact directly with variable regions of the exported polysaccharide. If true, it is likely that this *kpsT*-to-K-type association will hold for other K-types. Due to its location inside the cell, KpsT is unlikely to be a candidate antigen for vaccine consideration (see Fig. S2). However, these results do have important implications outside vaccine development. For one, K-typing can be achieved by simply sequencing this gene, thereby allowing for K-types to be reported alongside phylogroup and virulence factors in clinical studies. This will be especially important for rarer K-types. It could also allow for rapid bacteriophage therapeutic selection, since several types of phages specifically target capsule (73, 74). Finally, our results suggest that KpsT could be an attractive drug target, since disruption of this gene should be sufficient to prevent capsule export, thus targeting can be focused on K-types that are associated with extraintestinal pathogenesis while sparing commensals.

### Fimbriae and adhesins are associated with phylogroup rather than pathotype.

**FIG 4:**
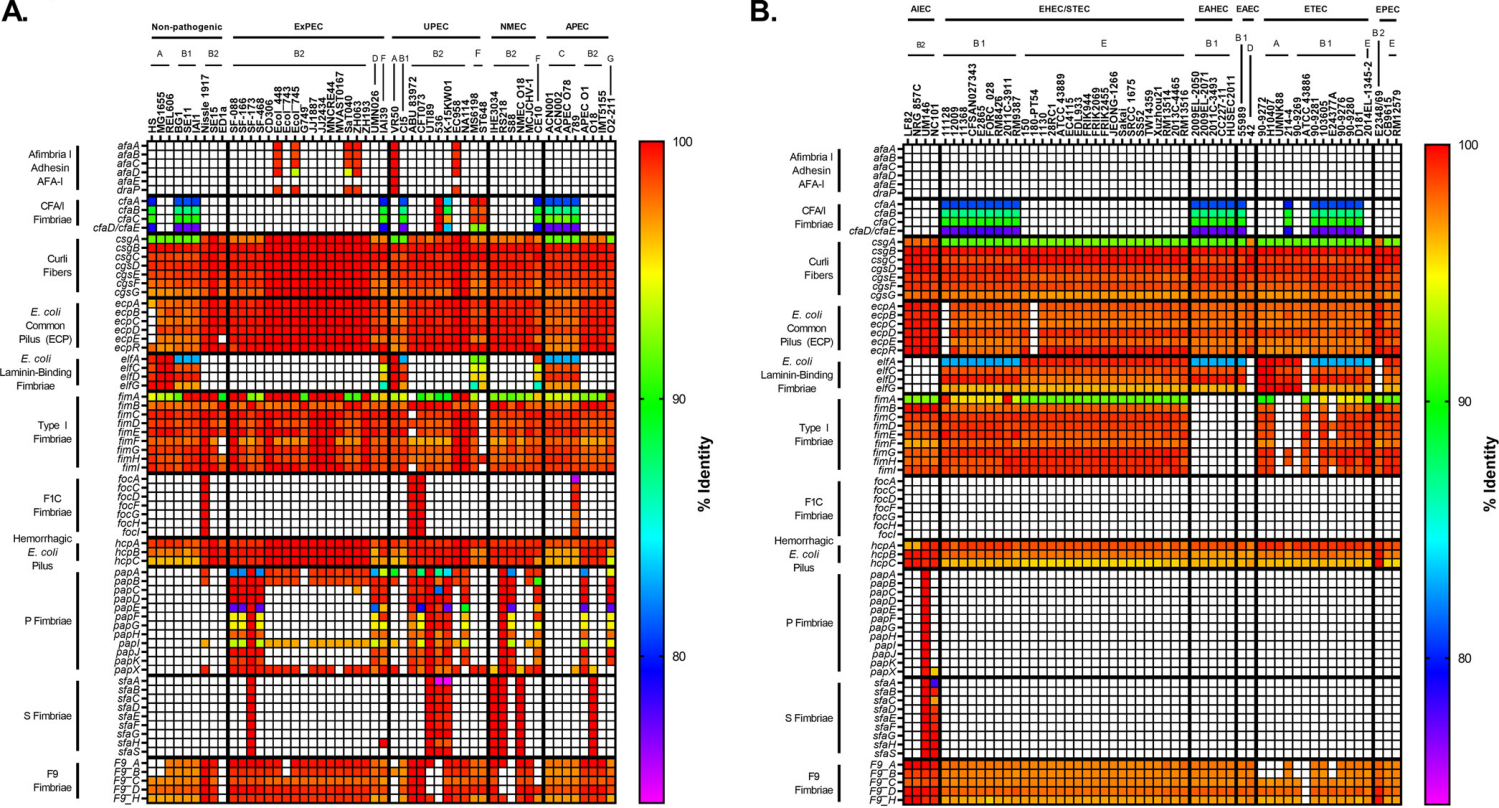
Pathotype distribution of adherence fimbriae. (A) megaBLAST percent identity results for nonpathogenic and ExPEC strains. (B) megaBLAST percent identity results for InPEC strains. The percent identity was determined using megaBLAST with the reference genes in Data Set S1 in the supplemental material.

Fimbriae and adhesins are proteins found on the surface of all E. coli. Many are thought to be associated with adhesion to certain molecules, environments, or cell types (75). They are highly immunogenic and, if their distribution and mechanisms can be understood, may make excellent vaccine targets (76). Our results showed that fimbriae tend to be associated with phylogroup rather than pathotype (Fig. 4).

Type I fimbriae are encoded by the *fim* operon, target α-mannose, and are known to be in both commensals and pathogenic E. coli (77–81). It has been reported to bind several other molecules and cell types, including collagen, fibronectin, laminin, and macrophages (82–85). AIEC adherence to intestinal epithelial cells of Crohn’s disease (CD) patients is dependent on type I fimbriae, apparently because of the overexpression of CEACAM6 in CD patients (86). These fimbriae have been shown to promote urinary tract colonization and persistence, as well as cellular invasion (87, 88). However, there are some conflicting results where the ABU strain 83972 (which lacks the complete *fim* operon) did not promote adherence in a murine urinary tract when *fim* was complemented (89). Our results show that type I fimbriae are indeed widely distributed and well conserved in nonpathogenic, ExPEC, and InPEC strains (Fig. 4). Surprisingly, EAEC and EAHEC appear to completely lack the *fim* operon, and this absence does not correlate with phylogroup, sequence type, or serotype (Fig. 4B). This is despite the fact that the *fim* operon of prototypical EAEC 042 strain has been studied (90). It is possible that the F9 or *sfm* fimbriae that are found in 042 were mistaken for the type I fimbriae given their similarity and the fact that they are often mistakenly annotated as *fim*. In each case for EAEC and EAHEC, the only hit found for the *fim* locus is a partial 171 bp hit for *fimH.* This partial hit is found in the correct location on the chromosome (between the *gntP* and *nanC* genes), but it is interrupted by an IS*1*-family transposable element. This supports the notion that the B1 EAEC and EAHEC strains studied here may share an ancestor. Interestingly, of the eight non-EAEC/EAHEC strains lacking the full *fimH* operon, this partial 171-bp hit and transposable element was also found in the ST648 (ExPEC; F phylogroup) strain and 90-9281 (ETEC; B1 phylogroup). It should be noted that there are two E. coli 042 strains found in the NCBI database. The one analyzed here has a 5,241,977-bp chromosome with accession number FN554766 (RefSeq NC_017626) and has had its analysis as an EAEC strain published (91). The other has a 4,692,707 bp chromosome with accession number CP042934. This entry does not have an accompanying article, and the title gives no indication that it is an EAEC or EAHEC strain.

The afimbrial adhesin Afa is encoded by the *afa* genes and is generally associated with ExPEC, UPEC, and diffusely adhering E. coli (DAEC) strains (81, 92). The vast majority of strains containing *afa* genes belonged to the ST131 clonal group (Fig. 4). The full set of genes to make this adhesin protein were only found in the VR50 strain (A; ABU/UPEC), which is an asymptomatic bacteriuria strain (Fig. 4A) (93). But this is because there are multiple divergent alleles of *afaE*, and a single reference (*afaE-I* was used here) will not cover them all using our method (94). The limited distribution of *afa* genes make it a poor vaccine target by itself, but combining it with other antigens in a polyvalent vaccine is a viable option.

**FIG 5:**
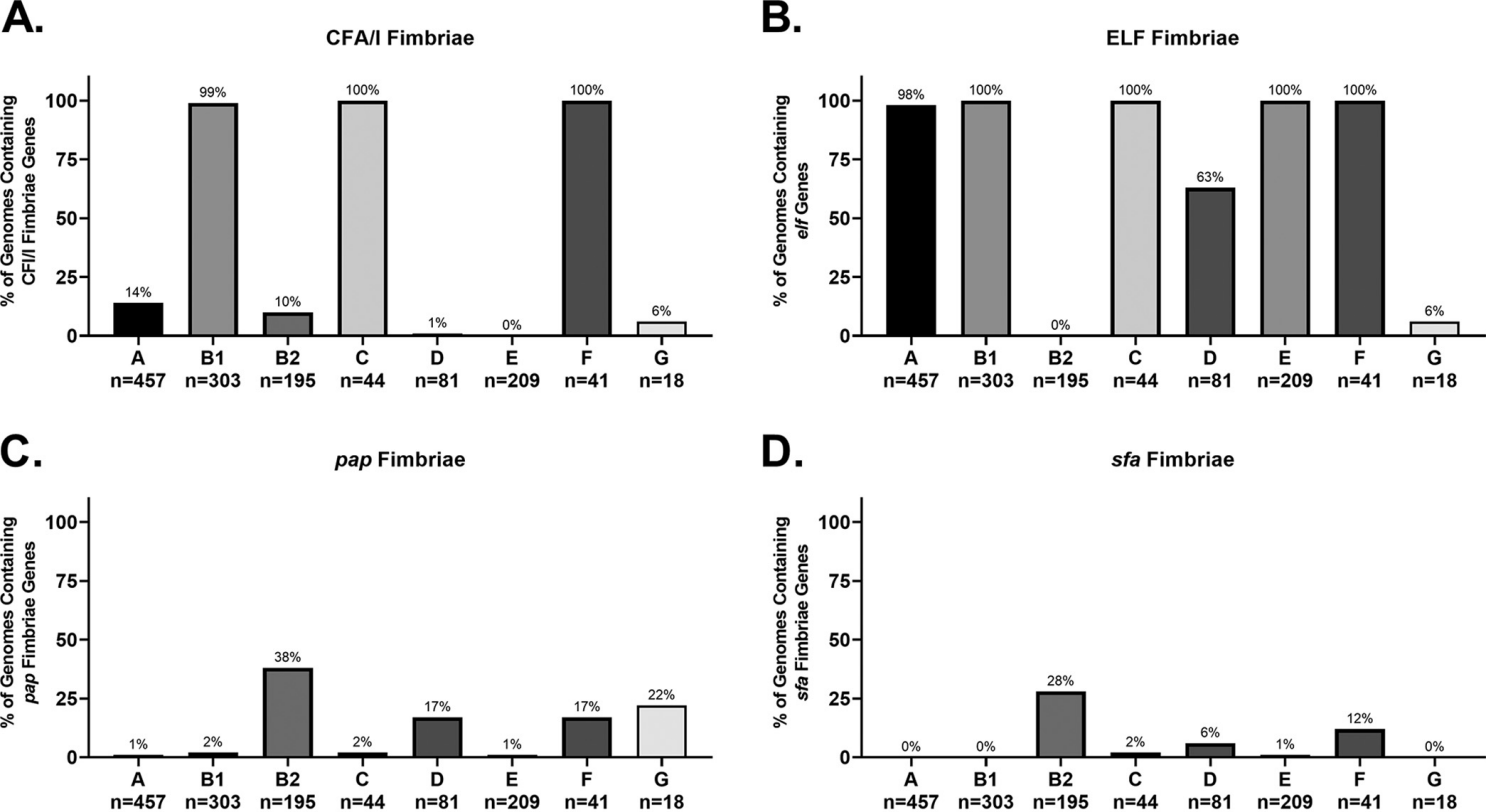
Phylogroup distribution of select fimbriae. (A) Distribution of CFA/I fimbriae, which has until now been associated with ETEC strains. (B) Distribution of the ELF fimbrial genes. (C and D) Distributions of UPEC-associated P and S fimbriae, respectively. Note that only *papCDEFH* genes were used to differentiate strains that contained a full array of *pap* genes and those that contain a disrupted *pap* operon. Distributions were determined using megaBLAST to bin strains from each phylogroup into hit versus no hit.

Colonization factor antigen I (CFA/I) fimbria is a class 5 fimbria generally considered associated with human colonization of ETEC strains (95). The CFA/I fimbriae are found in ETEC strains are encoded by a plasmid, but there is apparently a divergent form (∼30% identical to the ETEC plasmid version) on the chromosome that was widely found in strains belonging to the B1, C, and F phylogroups (Fig. 4 and 5). This trend is verified by our phylogroup database, where 100% of B1, C, and F strains contain hits for CFA/I (Fig. 5A). The most surprising result here is that all strains from the F phylogroup in both our databases contained this type of fimbria because the F phylogroup is more closely related to B2 strains (Fig. 4 and 5). This finding may be a clue to the lifestyle of the understudied F phylogroup. Interestingly, the only B2 strains that contained CFA/I were those from ST127 (Fig. 4A). Given that B1 strains are most often found as commensals in domesticated animals, it is possible that this fimbria promotes colonization in nonhuman hosts. Our finding also suggests that CFA/I as a vaccine target against ETECs will need to be carefully studied, since there is a possibility of cross-reactivity with nonpathogenic strains.

E. coli laminin-binding fimbriae (ELF) were first described in EDL933, an O157:H7 EHEC/STEC strain, where is was found to contribute to the ability to bind Hep-2 cells (96). That group also found that antibodies against ELF were able to partially block adherence of EDL933. Our results suggested that ELF is more generally found among strains that do not belong to the B2 or D phylogroup (Fig. 4 and 5). In fact, it appears that no strain from the B2 phylogroup (0/39; 0%) in either our pathotype database or phylogroup database (0/195; 0%) harbor ELF on their chromosome, but it is found in all other phylogroups in our pathotype database (Fig. 4 and 5). It was also found in more than 97% of strains from phylogroups A, B1, C, E, and, surprisingly, F in our phylogroup database (Fig. 5B). It is interesting that, like CFA/I fimbriae, ELF is found in a phylogroup F that is more closely related to phylogroups B2 and D, which are generally considered to be more like ExPECs. This all makes ELF unlikely to be a viable vaccine target for either ExPECs or InPECs, but again may offer clues to the niche of phylogroup F strains.

Fimbriae of serotype 1C (F1C) is encoded by the *focA*, *focC*, *focD*, *focF*, *focG*, *focH*, and *focI* genes (97). These fimbriae are associated with uropathogenic strains and selectively bind to glycosphingolipids found on bladder, urethra, and kidney cells (98–101). It has also been shown to play a role in intestinal colonization of Nissle 1917 (nonpathogenic) (102). Despite the association with uropathogenicity, our search located true F1C fimbria hits in only three strains—Nissle 1917 (nonpathogenic), ABU 83972 (UPEC), and CFT073 (UPEC)—but that several cross hits with *sfa* genes did occur. The three strains with F1C were all members of the ST73 sequence type, which may indicate that this F1C is specific to certain sequence types.

F1C fimbriae are closely related to S-fimbrial adhesins, which are encoded by *sfa* genes and found on PAI III_536_ (103). However, they have distinct receptors, with S-fimbria-binding sialyl galactosides (104, 105). This matches our results where the *foc* reference genes from CFT073 (UPEC) showed significant homology for *sfa* reference genes from the UTI89 (UPEC) strain. The major differences were found in the major subunits *focA* and *sfaA*, which only shared 75.7% identity, and the adhesins *focH* and *sfaH*, which shared 84.8% identity. The other genes of each loci could be considered alleles of the same gene: the first minor subunits (*focI* and *sfaD*) shared 98.7% identity, the second minor subunits (*focF* and *sfaG*) shared 99.2% identity, the periplasmic chaperones (*focC* and *sfeE*) shared 98.7% identity, and the outer membrane ushers (*focD* and *sfaF*) shared 99.6% identity. The second gene downstream of the *sfa* locus is a previously uncharacterized bona fide *papX* regulator (100% coverage, 96% identical to reference *papX*). The *sfa* locus also contains two other regulatory genes that are like *pap* genes: *sfaB*, which shares high similarity with *papB*, and *sfaC*, which shares high similarity with *papI*. This all suggests a connected evolutionary history between these three fimbriae and may link them with uropathogenicity. On a practical level, this relatedness made it difficult to determine whether to score hits as *sfa* or *foc*. In the present study, we considered the hits to be hits for the F1C fimbriae if a full-length *focG* was present and S fimbriae if *sfaS* was present. In general, this method agreed with deciding which fimbriae were present by determining whether the adhesin was a better hit for *focH* or *sfaH*. However, strain 789 (APEC) presents an interesting problem for either way: this strain contains what appears to be a *focH* adhesin in an operon that is predominantly made up of S-fimbrial genes (>99% identical), with the exception of the minor subunit *sfaS*, which diverged and only had a minor hit (Fig. 4A). This may be an uncharacterized hybrid F1C/S fimbria. For strain 789, hits were included under both the F1C and S fimbria subsection to highlight the relatedness. The divergences between the traditional targets of fimbria vaccines, major subunits *focA* and *sfaA* and adhesins *focH* and *sfaH*, signifies a high probability that F1C and S fimbriae could only be targeted together with a polyvalent vaccine. However, the relatedness of the minor subunits means that they should be explored as potential targets.

P fimbriae are encoded by *pap* (pyelonephritis-associated pili) genes and are associated with uropathogenic strains and target glycosphingolipids (101, 106). It is part of both PAI I_CFT073_ and PAI II_CFT073_ pathogenicity islands (107). Some studies have found that P fimbriae are associated with 90% of acute pyelonephritis but less than 20% of ABU strains (80). Many ExPEC and NMEC strains contained only a few of the genes required for P-fimbria production, including the major repeating subunit, *papA* (Fig. 4). In some cases, such as in strains belonging to the ST131 group, full true hits for *papA*, *papB*, *papI*, and *papX* were found with nearby mobile elements that may explain the absence of the rest of the loci. In others, there were hits for *papB* and *papI* that appeared to be cross-hits with some S-fimbria genes (*sfaB* and *sfaC*, respectively). There were *papX* hits returned in any strain with the P or S fimbriae due to the *papX* homologue found near the *sfa* operon and in fact was found in over 70% of B2, D, and F strains in our phylogroup database, indicating that it may be ancestral to this lineage. It is important to note that it is possible that the bias toward certain sequence types (i.e., ST131 and ST95) in our pathotype database may skew these percentages, but the *papB/sfaB* hit does cover the majority of sequence types found in the ExPEC and NMEC category (Fig. 4). Interestingly, the only ST131 with a full complement of *pap* genes was NA114, a UPEC strain isolated in India (Fig. 4) (108). On the other hand, 70% (7/10) of ST95 strains, 67% (2/3) of ST73 strains, and 100% (3/3) of ST127 strains carried the full complement of *pap* genes (Fig. 4). P-fimbrial genes are only rarely found outside the B2/D/F/G clade in our phylogroup database (Fig. 5).

The *afa*, *foc*, *pap*, and *sfa* genes are considered markers for ExPEC potential. However, the results from our pathotype and phylogroup databases suggest that these genes are found almost exclusively in the B2, D, F, and G cluster and are found in less than 2% of strains from the A, B1, C, and E cluster (Fig. 5). Its presence in the latter cluster could be explained by horizontal gene transfer (HGT), but its presence in the former could be more complicated. The leading hypothesis is that these genes give a competitive advantage in intestinal colonization (2, 49, 50). This may be related to the fact that *elf* and CFA/I fimbriae are well conserved in phylogroups associated with commensalism or intestinal pathogenesis but lacking in B2 and D strains. This again makes the F phylogroup intriguing because it contains both of those fimbriae but can also carry the ExPEC-associated fimbriae. No matter which proposed evolutionary route for phylogroup diversification turns out to be correct, the presence of ELF and CFA/I in F strains indicates these fimbriae were acquired or lost in the population multiple times in other phylogroups.

A polyvalent fimbria vaccine targeting *afa*, *foc*, *pap*, and *sfa* could potentially target most strains responsible for ExPEC infections. Although these fimbriae are mostly found in the B2, D, F, and G clade, this clade is responsible for the majority of extraintestinal infections. Importantly, such a vaccine could also conceivably prevent long-term colonization by ExPEC, since these adhesins appear to contribute to colonization of the intestines, and many B2 and D strains seem to lack fimbriae found in other intestinal E. coli, such as CFA/I and ELF. This vaccine would also target ExPEC strains from other phylogroups that have acquired them through HGT, such as VR50 (A; ExPEC) and 789 (C; APEC). The most logical targets of such a vaccine would be the adhesin or major subunit, but the overlap between minor subunits of F1C and S fimbriae should be investigated as well.

One major question remains with P fimbriae: which protein to target. Of the highly conserved genes, *papB*, *papI*, and *papX* encode regulatory proteins, excluding them as potential targets (109–111). The only other highly conserved gene is *papA*, which encodes the major subunit. However, some studies have shown it to be dispensable for binding when *papE* is present, unlike *papF* and *papG*, which are required for binding (75, 112). This presents more questions than answers: conservation of *papB*, *papI*, or *papX* can be explained by *trans*-regulatory functions (109–111). The conservation of *papA* is, at first glance, a mystery. However, upon closer inspection, it appears that *papA* may be susceptible to transposon insertion, since it appears to have been disrupted multiple separate times based on alignments of *papA* (see Fig. S4 in the supplemental material). Given this, *papH* appears to be the best target for vaccine intervention.

### Strains from the B2 phylogroup are enriched for iron acquisition genes.

**FIG 6:**
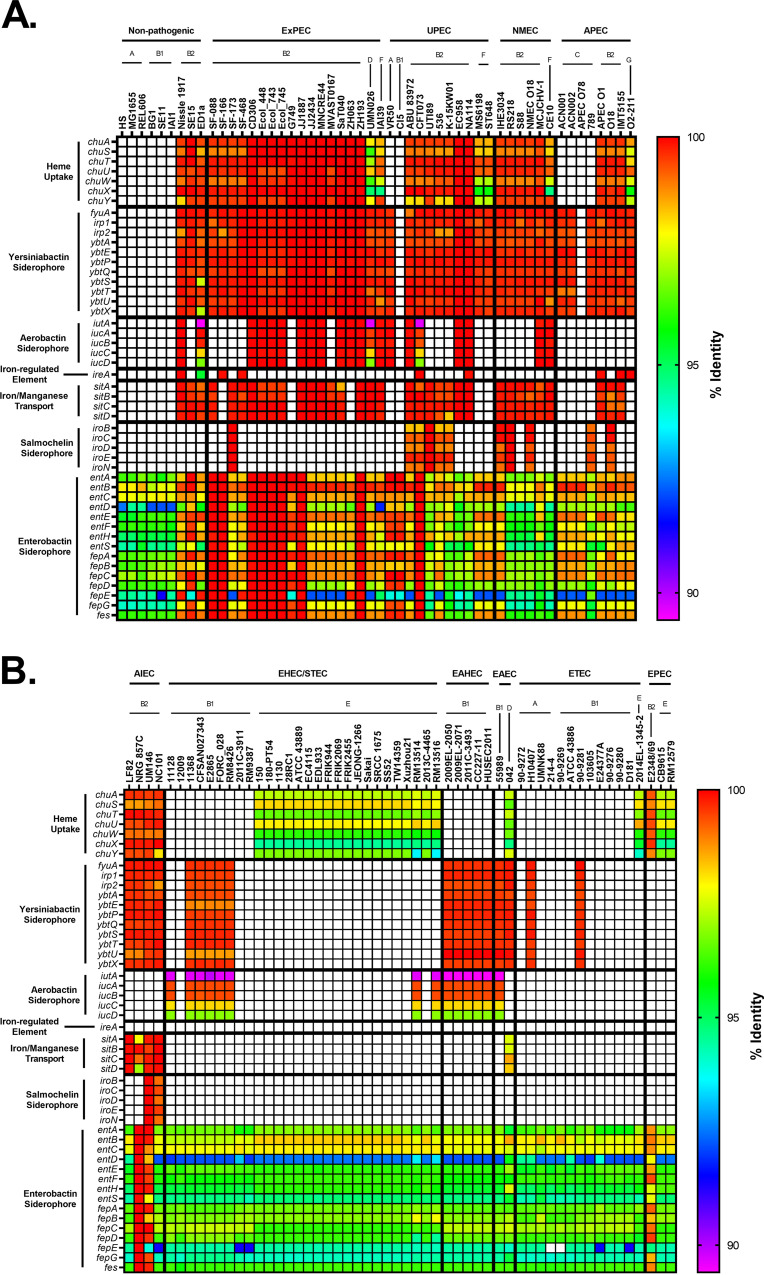
Pathotype distribution of iron acquisition genes. (A) Nonpathogenic and ExPEC strains. (B) InPEC strains. The *chu* operon is responsible for heme uptake. Yersiniabactin, aerobactin, and salmochelin are virulence-associated iron-binding siderophores, whereas enterobactin is a ubiquitous iron-binding siderophore. The percent identity was determined using megaBLAST with the reference genes in Data Set S1 in the supplemental material.

**FIG 7:**
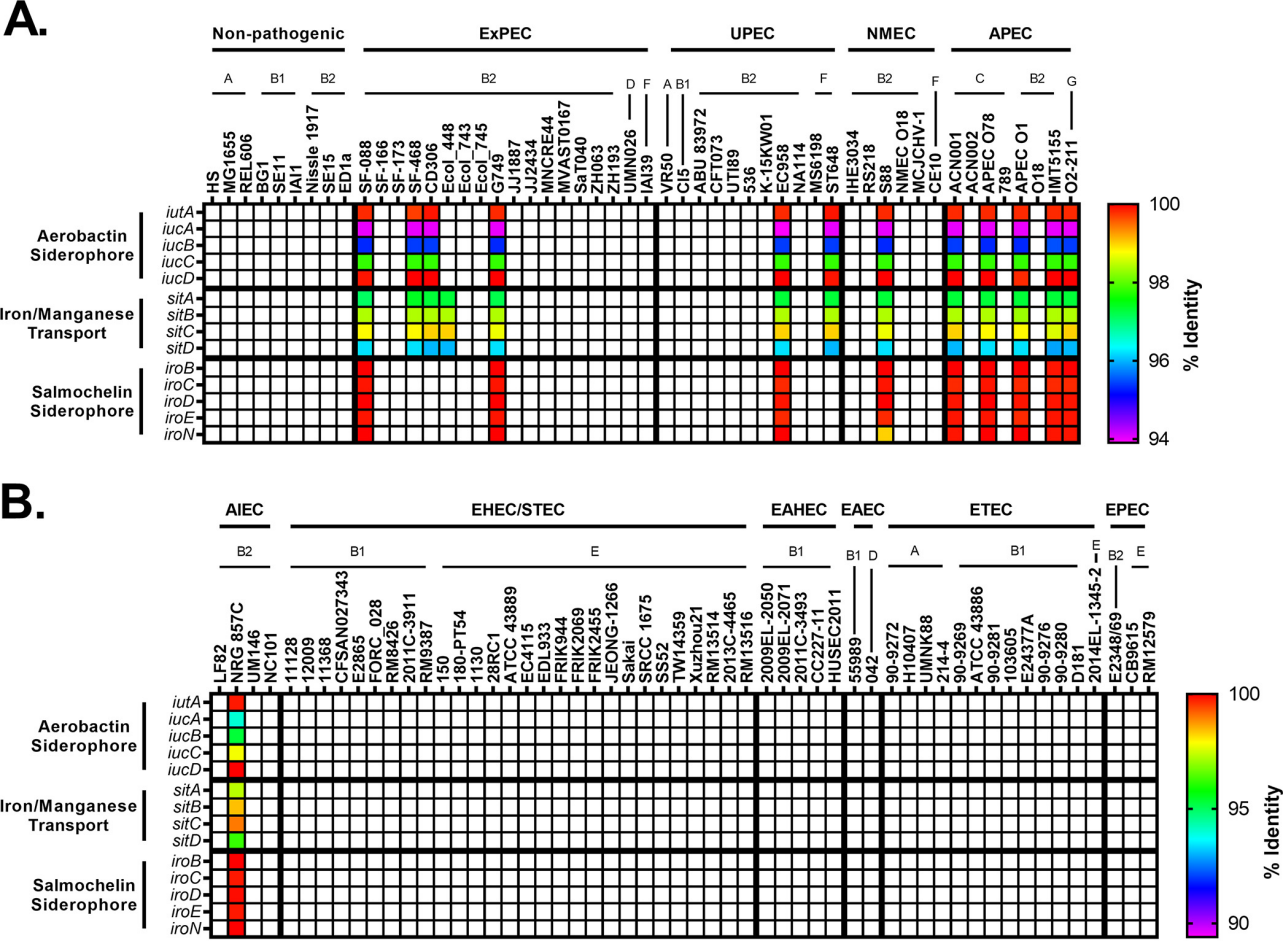
Distribution of plasmid-encoded iron acquisition genes. (A) Nonpathogenic and ExPEC strains. (B) InPEC strains. Plasmids for each strain in the pathotype database were obtained and analyzed using megaBLAST against the iron acquisition data set. The percent identity was determined using megaBLAST with the reference genes in Data Set S1 in the supplemental material.

Iron acquisition proteins have long been known to be associated with virulence because iron is a limiting essential nutrient for pathogenic bacteria in the host (24, 113–116). Hypothesizing that the pathogenic pathotypes may harbor a preponderance of such genes, we compared genes encoding siderophores, hemophores, and iron transporters between pathogenic and commensal strains. Our results show that pathogenic strains are enriched for iron acquisition genes (Fig. 6). The three exceptions to this were the nonpathogenic phylogroup B2 strains ED1a, Nissle 1917, and SE15 (Fig. 6A). This general trend of increased iron acquisition may be another explanation for why members of the B2 and D phylogroups are overrepresented in ExPECs, since recent work has shown that iron acquisition gene increase intrinsic virulence (117). Because several pathogenic bacteria lacked these virulence-linked iron acquisition genes on their chromosomes, we also searched their plasmids (Fig. 7).

ExPECs tended to have more iron acquisition than InPECs (Fig. 6). With non-APEC ExPECs, only 5 (G749, CI5, MS6198, ST648, and VR50) of 35 (14.3%) strains carry fewer than three of these iron-uptake loci (Fig. 6A). InPECs tended to have few iron acquisition types, with only AIECs and strain 042 (EAEC; D) having more than two of the loci examined (Fig. 6B). All of these trends can be explained by phylogeny: of the five ExPECs deficient in virulence-associated iron acquisition, only one was a B2 strain (Fig. 6A). EHECs/STECs tended to have either the *chu* heme operon or the yersiniabactin siderophore system, depending on whether they are from the E phylogroup (*chu*) or the B1 phylogroup (yersiniabactin) (Fig. 6B). This may suggest that EHEC/STEC strains from different phylogroups have different primary sources of iron during infection, since the *chu* operon is responsible for the uptake of heme, whereas yersiniabactin targets ferric iron (Fe^3+^) with an affinity that would allow it to steal iron from host iron-bound proteins, including transferrin and lactoferrin (118, 119). The A, B1, and C phylogroups lacked *chu* because, by definition of the phylogroups, they lack *chuA*. Interestingly, the ETEC pathotype seems to be the most devoid of iron acquisition genes, possibility indicating a close relationship with commensals, which has been noted for some ETEC strains (120).

**FIG 8:**
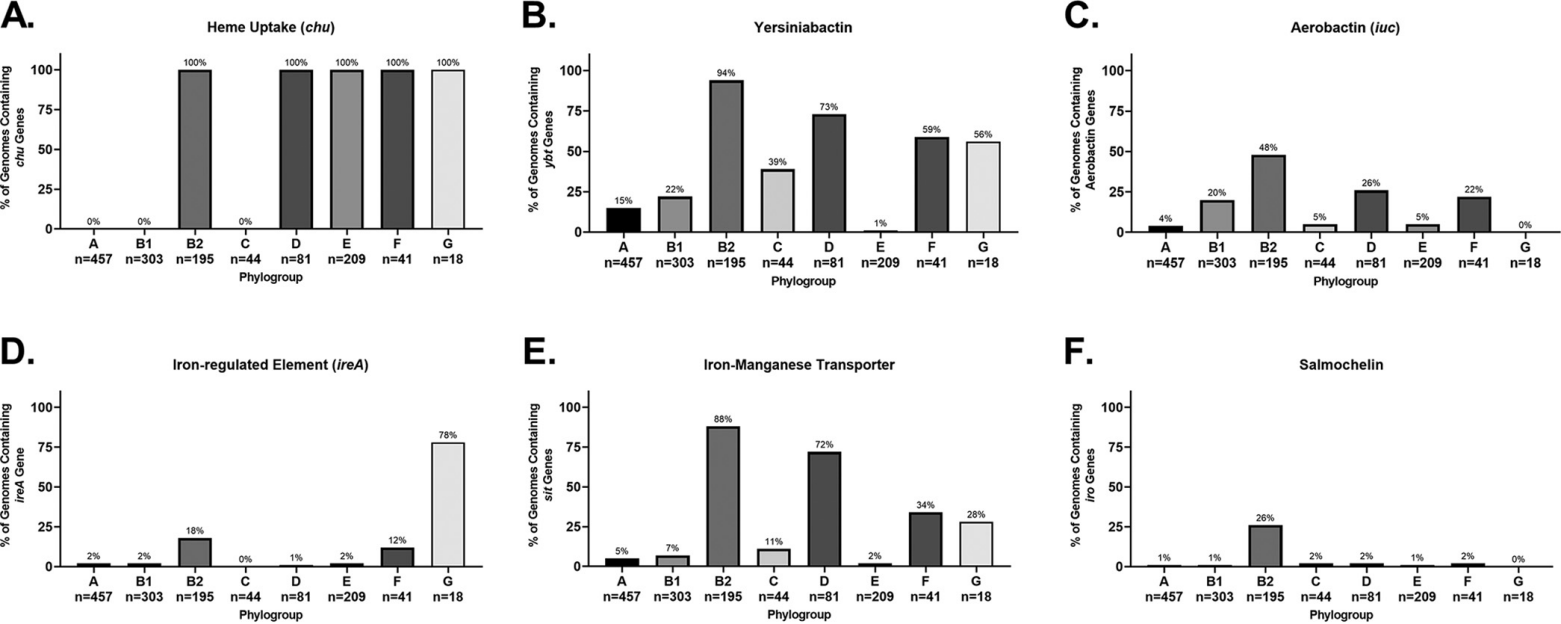
Phylogroup distribution of iron acquisition genes. (A) Distribution of the heme uptake genes: *chuASTUWXY*. This is an important control for our phylogroup database and shows that the B2/D/F/G clade and E phylogroup are correctly distinguished from the A/B1/C clade. (B) Distribution of strains carrying any of the yersiniabactin genes: *fyuA*, *irp1*, *irp2*, and *ybtAEPQSTUX*. (C) Distribution of strains carrying any of the aerobactin genes: *iucABCD* or *iutA*. (D) Distribution of strains carrying the iron-regulated gene *ireA*. (E) Distribution of strains carrying any of the iron/manganese transporter genes: *sitABCD*. (F) Distribution of strains carrying any of the salmochelin genes: *iroBCDEN*. Distributions were determined using megaBLAST to bin strains from each phylogroup into hit versus no hit.

In the phylogroup database, our results for *chu* show that it is found in 100% of B2, D, E, F, and G strains (Fig. 8A). This is an important control for our method since these phylogroups are characterized by the presence of *chuA.* Still, the *chu* system has been shown to play an important role in uropathogenesis (121, 122). The distribution of yersiniabactin, encoded by *fyuA*, also agrees with the results from our pathotype database and highlights the trends more clearly. Over 90% of B2 strains carry yersiniabactin (*fyuA*), a siderophore found on PAI IV_536_ (Fig. 8B) (117). It is surprising to find that such an overwhelming majority of B2 strains carried *fyuA* because it is a well-known ExPEC-associated gene. In fact, it has been proposed as a gene to differentiate UPEC strains from commensals and other pathotypes (123). While *fyuA* is only found in 22% (68/303) of B1 strains, 42 of those strains carry the *stx* toxin, making them EHEC or STEC strains (Fig. 8B). Finding yersiniabactin in many B1 EHEC/STEC strains does make sense, however, since unlike members of the E phylogroup (including the well-known O157 serotype), B1 strains do not have the *chu* system to extract iron from blood. This could also explain why less than 1% of E-phylogroup strains contain yersiniabactin: the majority of those strains are members of the EHEC/STEC pathotype, and they all have the *chu* system to extract heme-iron after inducing bloody diarrhea (Fig. 8A and B). This does lead to some obvious questions about selection in cattle, where EHEC/STEC strains are found as asymptomatic intestinal residents. Yersiniabactin does not appear to be highly conserved, but it is enriched for in both the B2/D/F/G clade and the C phylogroup. This pattern could be explained by extensive horizontal transfer coupled with the lack of strong positive selection during intestinal colonization and commensalism. The presence of yersiniabactin in the C phylogroup is probably related to APEC strains from that phylogroup: three of our four strains matching that criteria in our pathotype database contained the siderophore (Fig. 6A).

Aerobactin is a siderophore found on PAI I_CFT073_ that has been shown to be important for uropathogenesis (124, 125). The aerobactin locus is found throughout pathotypes and phylogroups, which is not surprising considering it is found on a mobile element (Fig. 6 and 8). There do appear to be two distinct forms of the operon, with one form being found predominantly in InPEC strains of the B1 phylogroup and the other being found mostly in ExPEC strains of the B2 phylogroup, which raises questions about how or whether this locus is transferred between phylogroups or just within them (Fig. 6). Between these forms of aerobactin, the biggest divergence was found in *iutA* (89.6% identical to ExPEC ST131 reference), the gene encoding the aerobactin receptor, which may make it difficult to target with a vaccine. This divergent *iutA* is predominantly found in strains from the B1 and E phylogroups, but there are instances of it being found in the B2 phylogroup, such as with CFT073 (Fig. 6). This is interesting because CFT073 belongs to the ST73, which also contains the ABU 83972 (UPEC) strain and the Nissle 1917 (Nonpathogenic) strain, two strains that carry the less-divergent *iutA* gene (Fig. 6A). There is less divergence in the chromosomal versions of *iucC* and *iucD*, which are aerobactin biosynthesis genes (Fig. 6). Of the 61 B1 strains that carry *iutA*, 89% (54/61) belong to the EHEC/STEC pathotype (*stx*^+^). Outside these EHEC/STEC strains, *iutA* is again found predominantly in the B2, D, and F cluster (Fig. 8C).

Genes encoding aerobactin were also found on numerous plasmids from the B2, F, and C phylogroups and was in 63% (5/8) of the APEC strains (Fig. 7). The plasmid version of aerobactin appeared to be nearly identical across plasmids, regardless of pathotype or phylogroup, indicating that this plasmid is probably widespread and contributes to virulence (Fig. 7). The plasmid-encoded version was different from the chromosomally encoded version (Fig. 6 and 7). They all contained the ST131-like *iutA* gene but differed significantly from the chromosomal version in their biosynthetic genes. This indicates that they probably diverged long ago, but the actual receptor was conserved. The plasmid carrying the aerobactin operon also contained nearly identical operons for the *sit* (iron-manganese transporter) and *iro* (salmochelin) iron acquisition genes (Fig. 7). This plasmid is the pS88/pColV plasmid associated with APEC virulence and NMEC strains (126, 127).

Iron-regulated element A (*ireA*) is a siderophore-receptor like protein that is associated with ExPEC strains, implicated in adherence, and found on PAI_CFT073_ II (128). It appears to be present only sporadically in members of the B2 phylogroup and strain O2-211 (APEC), which is a member of the recently characterized G phylogroup (Fig. 6). This gene is relatively rare in our phylogroup database, being found in less than 3% of strains in phylogroups A, B1, C, D, and E (Fig. 8D). However, it is still only found in 20% of phylogroup B2 strains and 12% of phylogroup F strains. Surprisingly, it is found in 78% (14/18) of phylogroup G strains in our database (Fig. 8D).

The iron-manganese transporter system encoded by the *sit* locus was found almost exclusively in the chromosome of the B2/D/F/G cluster and follows a pattern similar to yersiniabactin, except for being less common in the C phylogroup (Fig. 6). Only 4 of the 39 strains (10%) belonging to the B2 phylogroup in our pathotype database lacked this transporter: G749, SF-166, and ZH063 (all ExPECs) and E2348-69 (EPEC), though G749 carried it on a plasmid (Fig. 6 and 7). Distribution of *sit* in our phylogroup database agrees with this, with *sit* being present in 88% of B2 strains, 72% of D strains, and 34% of F strains (Fig. 8E).

The salmochelin siderophore encoded by the *iro* genes on the PAI III_536_ and has been implicated in the adherence and invasion of urothelial cells and virulence in an animal model and is upregulated in the presence of human urine (103, 129–133). This locus is found only on the chromosome of a few strains from the B2 phylogroup, mainly from the UPEC (46%; 5/11), NMEC (50%; 3/6), and AIEC (50%; 2/4) pathotypes, from the ST73, ST95, and ST127 sequence types (Fig. 6). Our phylogroup database saw a similar distribution: *iroN* was found in only 26% of B2 strains and less than 2% of strains from other phylogroups (Fig. 8F).

Iron acquisition genes have a well-known and extensively studied association with uropathogenesis (116, 121, 122, 124, 134–137). Indeed, vaccines targeting siderophores have been proven as a concept in animal models, including mouse models of both E. coli urinary tract infection (UTI) and intestinal colonization by Salmonella (138–145). While some of these studies showed that protective antibodies against siderophores can be generated, the results are not as efficacious as one would hope. This is potentially due to the high level of redundancy in iron acquisition genes seen in uropathogenic strains (135). Still, our work supports previous results suggesting that iron acquisition genes are good targets for an ExPEC vaccine. One obvious bonus to targeting iron acquisition genes is that the risk of targeting commensals may be lower with such a vaccine. This is also supported by evidence that different iron acquisition mechanisms have different levels of importance depending on the type and location of the infection (121, 122, 129, 135). Of course, there is also the risk of redundancy previously mentioned that could make it easy for E. coli to evolve resistance. A solution to this is a polyvalent vaccine. Targeting yersiniabactin, aerobactin, and *sit* would target many members of the B2, D, and F clade, while targeting *ireA* and salmochelin would target rarer sequence types and apparently UPECs specifically (Fig. 6 and 8). Targeting aerobactin, *sit*, and salmochelin would also vaccinate against the pColV plasmid that appears to be associated with ExPECs (Fig. 7).

### The B2 phylogroup is enriched for ExPEC-associated toxins.

**FIG 9:**
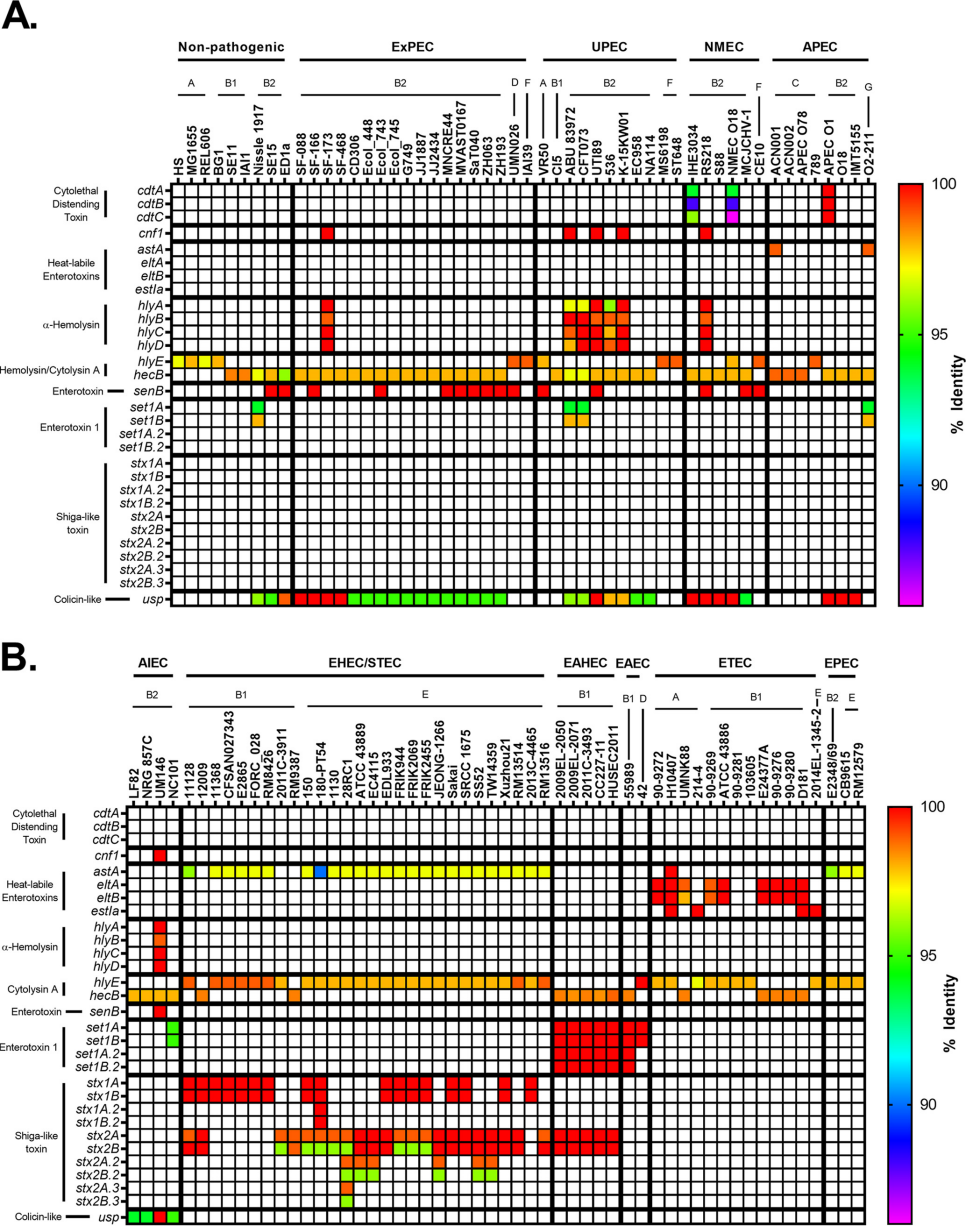
Pathotype distribution of toxins. (A and B) Percent identity results from megaBLAST alignments of nonpathogenic and ExPEC strains (A) and InPEC strains (B). The percent identity was determined using megaBLAST with the reference genes in Data Set S1 in the supplemental material.

Bacterial protein toxins have long been studied as critical virulence factors driving pathogenesis. In some cases, pathotype—such as EHEC/STEC and ETEC—assignment can be made based solely on the presence of certain toxins. While there are toxins known to contribute to ExPEC virulence, no single toxin can ensure an ExPEC phenotype. Like our iron acquisition gene analysis, plasmids were included in the toxin analysis because they are often carried on plasmids. However, unlike the iron acquisition graphs, there were no overlaps between genes on the chromosome and those on plasmids, so they are incorporated into a single graph (Fig. 9). Our analysis of E. coli for well-known toxins produced some surprising findings.

**FIG 10:**
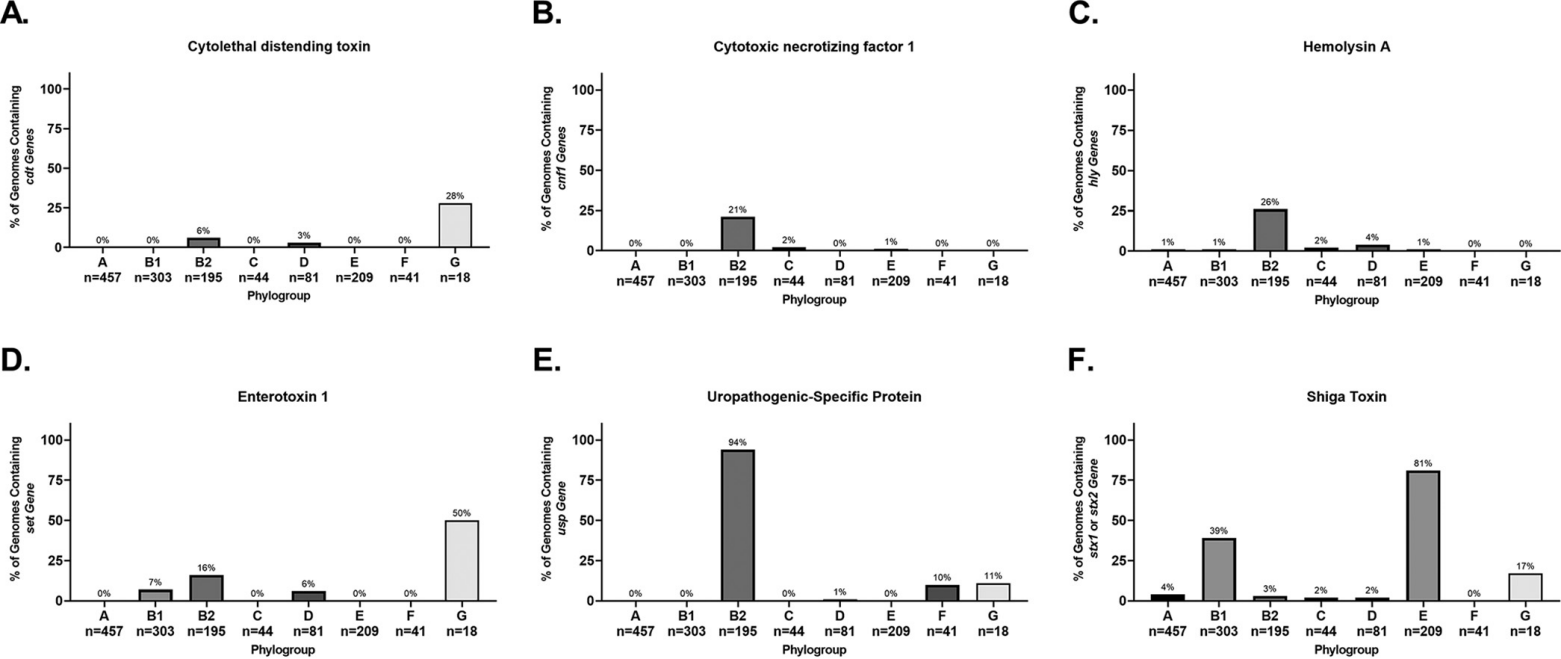
Phylogroup distribution of toxins. (A) Distribution of cytolethal distending toxin (*cdt*). (B) Distribution of cytotoxic necrotizing factor 1 (*cnf1*). (C) Distribution of chromosomal α-hemolysin (*hlyA*). (D) Distribution of chromosomal enterotoxin 1 (*setA1*). (E) Distribution of uropathogenic-specific protein (*usp*). (F) Distribution of phage-encoded Shiga toxin (*stx*_1_ or *stx*_2_). Distributions were determined using megaBLAST to bin strains from each phylogroup into hit versus no hit.

Cytolethal distending toxin (CDT) is a heterotrimeric genotoxin encoded by the *cdtABC* locus (146). After the CdtABC complex binds to a host cell, CdtB is delivered intracellularly where it causes double-stranded breaks and death of the host cell (146). In our pathotype database, *cdtABC* is only found in three B2 phylogroup strains, though two of them were from the ST95 sequence type (Fig. 9). This represented only 20% of ST95 strains in our database. In our phylogroup database, *cdtA* was also rare; only 19 of 1,348 strains carried it on their chromosome (Fig. 10A). The majority (*n* = 11) of these were strains from the B2 phylogroup, whereas 5 were from the G phylogroup, 2 were from the D phylogroup, and 1 was from the A phylogroup (Fig. 10A). Of these, the G phylogroup had the highest percentage (28%) of strains containing *cdtAB*, though there are only 18 strains in this phylogroup in our database (Fig. 10A).

Cytotoxic necrotizing factor 1 (CNF-1) is a chromosome-encoded deamidase toxin that is associated with UPEC and NMEC strains, and a virulence factor that is supposedly found in between 30 and 40% of ExPEC and diarrheal strains (147). The *cnf1* locus was not widely spread in our pathotype database, but it does appear to be associated with ExPEC strains and found only in strains from the B2 phylogroup (Fig. 9). These strains were from the ST73, ST95, ST127, and ST643 sequence types, though not all strains from these sequence types contained the *cnf1* gene. In our phylogroup database, there were only 43 hits for *cnf1*, and 40 of these were in the B2 phylogroup, but only in a low percentage of the total B2 strains (21%; 40/195) (Fig. 10B). The other three hits were in phylogroups E (*n* = 2) and C (*n* = 1). That *cnf1* was found in 30 and 40% of pathogenic strains suggests that an oversized number of ExPEC and diarrheal strains are caused by a relatively small number of strains, particularly from the B2 phylogroup.

Hemolysin E, also known as cytolysin A or silent hemolysin, is a pore-forming toxin encoded by *hlyE* that under certain conditions can confer hemolytic phenotype to E. coli carrying it (148, 149). It is found on PAI I_536_ and PAI II_536_ (103). It belongs to a family of toxins that is also found in Salmonella typhi and Shigella flexneri and reported to be a virulence factor in ETEC strains (32). It appears to have three chromosomal forms, all of which are more than 95% identical to the reference (Fig. 9). In over half of phylogroup B1 (15/27) and C (3/4) strains the *hlyE* gene is truncated by a frameshift, with both the 5′ and 3′ ends present and possibly translated (Fig. 9). This includes phylogroup B1 strains in the EAEC/EAHEC pathotype. In 100% of B2 strains (39/39), *hlyE* is truncated as well, but only the C terminus is present and is annotated as a hemolysin-activating protein, HecB, and it is more than 97% identical to reference *hlyE*. The full *hlyE* gene is found in the remaining (A, D, F, and G) phylogroups, as well as the other members of the B1 and C phylogroups. This truncation has been published before (148), but to our knowledge, no connection to phylogroup has been made before the present study, and the phylogroup distribution can explain the results showing a pathotype association.

α-hemolysin, also known as hemolysin A, is a pore-forming cytolytic toxin encoded by genes found on the PAI I_536_ and II_536_ pathogenicity islands and is only found on the chromosome of a few phylogroup B2 ExPECs and the plasmid of UMNK88 (ETEC; pUMNK88_Hly) (103, 150, 151). The chromosomal version is specifically found in strains from the ST73 (2/3; not found in Nissle 1917 commensal), ST95 (3/10), and ST127 (2/2) (Fig. 9). There is, however, another form that is found on the plasmids of many EHEC/STEC strains. This form is divergent enough to not be hit by megaBLAST alignments except for part of *hlyB* and is found on a plasmid of 82% of the EHEC/STEC strains examined: 78% (7/9) phylogroup B1 strains and 84% (16/19) of phylogroup E strains. Distribution of chromosomal *hlyA* in our phylogroup database shows that it occurs almost exclusively in strains from the B2 phylogroup, where it is found in 26% of strains (50/195) (Fig. 10C).

There has been some work on the distribution of *hlyA* and *hlyE*, such as by Kerenyi et al. (150), who looked at hundreds of clinical isolates for the presence of hemolytic activity, *hlyA*, and *hlyE* (referred to in that study as *shaE*). These researchers very reasonably concluded that *hlyA* and *hlyE* never occur together. Our results suggest an answer: *hlyA* occurs almost exclusively in phylogroup B2, and no B2 strain in our pathotype database contains the N terminus of *hlyE*.

*Shigella* enterotoxin 1 (ShET1) encoded by *setA1* and *setB1* genes that are found on the antisense strand of the *pic* gene in the SHI-1 (*she*) pathogenicity island. In *Shigella*, it has been found to induce intestinal fluid accumulation (152). It has been associated with EAEC infections but also found in many ExPECs as well (125, 153–155). In our pathotype database, this toxin was found to be highly associated with EAEC strains and found on the chromosome of a few B2 phylogroup members from very diverse pathotypes: Nissle 1917 (nonpathogenic; B2), NC101 (AIEC; B2), ABU 83972 (ABU-UPEC; B2), CFT073 (UPEC; B2), and O2-211 (APEC; G) (Fig. 9). However, three of these five strains (Nissle 1917, ABU 83972, and CFT073) belong to the ST73 sequence type. In B1 EAEC and EAHEC strains, the SHI-1 PAI was duplicated and led to two identical copies of each gene. It is interesting to note that all EAEC and EAHEC strains in our pathotype database contained very similar alleles of *setA1* and *setB1* (<0.06 and 0% divergence from the reference, respectively), despite being from diverse phylogroups (B1 and D). This is compared to *setA1* and *setB1* genes in B2 and G strains, which have roughly 5.6 and 1.6% divergence from the reference, respectively. This implicates genes carried on the SHI-1 PAI in EAEC pathogenesis, since it is likely that these divergent phylogroups acquired it independently, but recently. In our phylogroup database, *setA* and *setB* were found at low abundance, but predominantly in phylogroup B2 (Fig. 10D). The most surprising result is that 50% (9/18) of strains from the G phylogroup contained *set* genes (Fig. 10D).

Uropathogenic-specific protein (*usp*) is a colicin-like bacteriocin toxin that is associated with UPEC strains and increases virulence in a mouse model of UTIs (156). In our pathotype database, this supposedly UPEC-specific protein is actually highly associated with the B2 phylogroup, being found in all but one B2 strain: E2348/69 (B2; EPEC) (Fig. 9). Our pathotype database confirms this association: 94% of B2 strains and 10% of F and G strains contain this protein. It is only found in a single phylogroup D strain and none of the strains from the E phylogroup or the A/B1/C cluster (Fig. 10E). This distribution could explain the propensity for B2 strains to cause UTIs, and being so widespread suggests it entered the B2 phylogroup early in its diversification. Its lack of representation in other phylogroups suggests that it is not very mobile or highly selected against in some contexts. However, if it is not mobile, its presence in 10% (4/41) of the F-phylogroup strains is curious, especially since two of the three sequence types that are known to cause UTIs (ST62 and ST648) are represented in our pathotype database and yet do not contain *usp* (Fig. 9A) (54).

It is difficult to say whether or not some of these toxins can be used as a vaccine target, since many of them seem to be dispensable for ExPEC virulence. There has been some early success targeting α-hemolysin (hemolysin A) (157). Targeting α-hemolysin and/or CNF-1 could hit a subset of ExPEC strains, particularly some of the more common ExPEC strains from the ST73 and ST127 sequence types (Fig. 9). A vaccine targeting a combination of these proteins has been shown to significantly reduce instances of cystitis in a mouse model and bacterial loads in urine, but not colonization of the kidneys or bladder (158). However, these are both still found in only a small set of B2 strains and appear absent in ST131s. The *setAB* toxin could potentially be a target against EAECs and some virulent ExPEC sequence types (ST73). Targeting this toxin would also present a unique situation where the protein coded on the opposite strand—*pic*, an autotransporter—could also be a potential target. The so-called uropathogenic-specific protein (*usp*) could potentially act as a target for vaccines targeting B2 strains, but our results suggests that commensal B2 strains would also be targeted. Hemolysin E/cytolysin A also may present a B2 target, but it remains to be seen whether the truncated version found in B2 strains—often annotated as *hecB*—is functional and exported. Of all of these, we believe that α-hemolysin is the more attractive toxin target.

One thing our work does highlight is that the B2 phylogroup is by far the most likely phylogroup to contain these toxins, which does make targeting B2-associated antigens an attractive possibility.

### Type Va and Vc secretion systems (autotransporters) are associated with ExPEC strains.

Type V secretion system is composed of the autotransporters (Va or AT-1), two-partner secretion pathway (Vb), and trimeric autotransporter adhesins (Vc or AT-2) (159). This secretion system is made up of secreted and outer membrane proteins involved in adherence and virulence. They consist of N-terminal signal peptide, a passenger domain, an autochaperone domain, and a C-terminal transmembrane β-barrel domain (160). Other domains are possible, including a lectin-like domain found on the end of the invasion protein, Inv (161, 162). Autotransporters are classified based on their domain architecture as AIDA-I, serine protease autotransport of *Enterobacteriaceae*s (SPATEs), or trimeric autotransporter adhesin (TAA) (160). Overall, autotransporters in our data set were generally associated with ExPEC strains.

**FIG 11:**
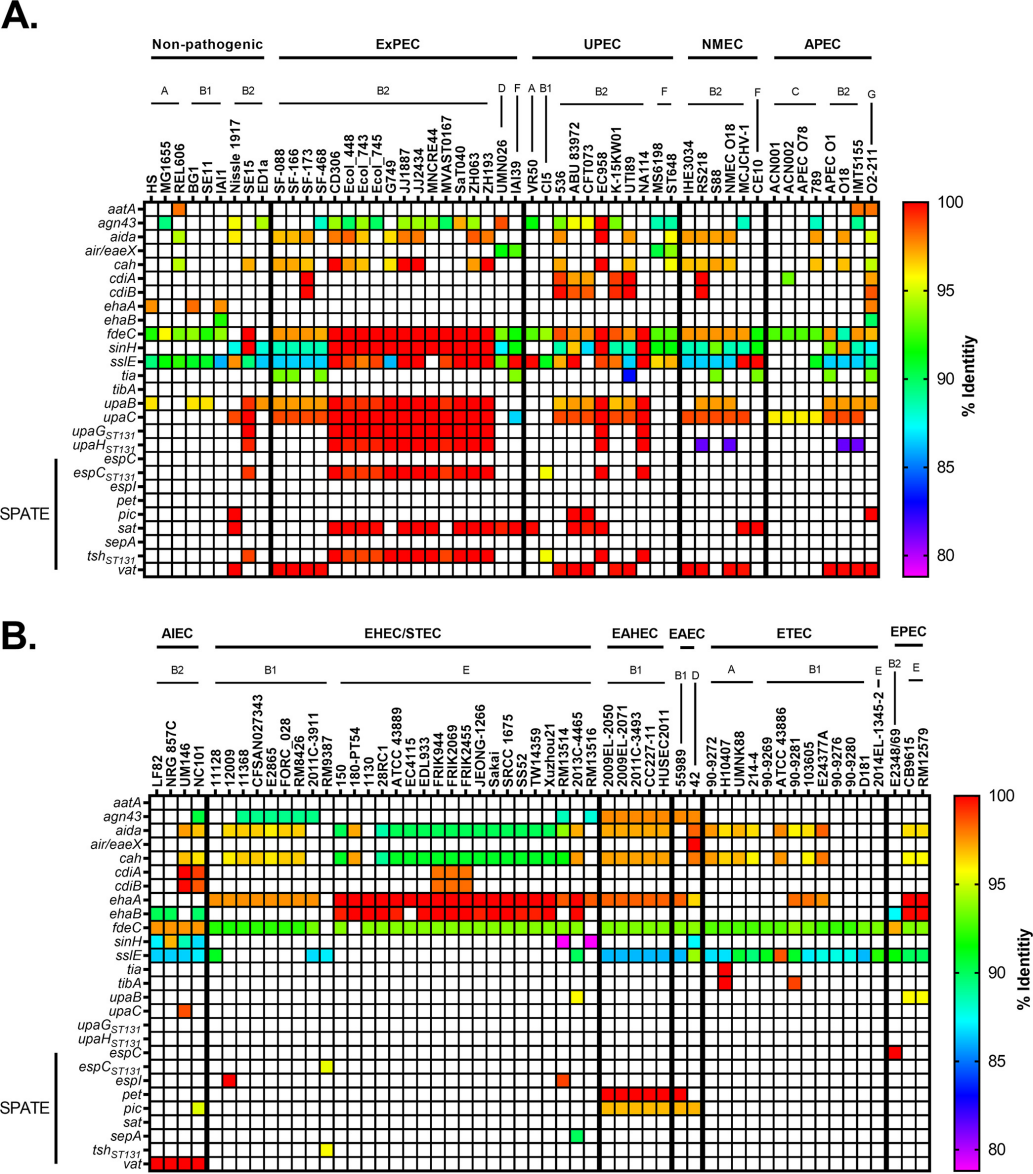
Pathotype distribution of autotransporters. (A and B) Percent identity results from megaBLAST alignments for nonpathogenic and ExPEC strains (A) and InPEC strains (B). SPATEs, serine protease autotransporters of *Enterobacteriaceae*. The percent identity was determined using megaBLAST with the reference genes in Data Set S1 in the supplemental material.

Antigen 43 (*ang43*), an AIDA-I member of the Va pathway, is one of the most abundant phase-varying outer membrane proteins and is encoded on PAI III_536_, and PAI I_CFT073_, along with the genes encoding the Vat or Sat autotransporter, respectively (103, 125, 163). This gene is also known as *flu* for fluffing because it promotes aggregation between cells and colonization of mouse bladders. Some evidence suggests it may be beneficial to UPEC strains (164). Our results suggest that *ang43* is widespread, with no apparent phylogroup or pathotype association (Fig. 11). There does appear to be an EAEC- and EAHEC-specific allele. This allele is found in all EAEC and EAHEC strains, regardless of whether they are from the D or the B1 phylogroup (Fig. 11), and appears to be on the same genomic island (GI 3 in strain 042) that carries *pic*, *set1A*, and *set1B* and two type VI secretion systems (see Fig. S4 in the supplemental material).

EhaA is an autotransporter identified as being associated with the O157:H7 serotype and important for adhesion and biofilm formation (165). However, our results suggest that *ehaA* is more generally associated with EHEC/STEC, EAEC, and EAHEC (Fig. 11). It is also possibly associated with EPEC strains but appears to be absent in the B2 EPEC strain (E2348/69).

EhaB, also called UpaC, is found in two forms: one (labeled *upaC*; ST131 is used as a reference) is found predominantly in ExPEC strains and B2 commensal strains (Nissle 1917 and SE15) (Fig. 11). However, it is absent in most AIEC strains (Fig. 11B). The other form, designated *ehaB* and using the Sakai (EHEC) strain as a reference, is found in 100% of O157 (15/15; all EHEC/STECs) and O55 (3/3; 1 EHEC/STEC and 2 EPECs) serotypes (Fig. 11). It is also found in the B2 phylogroup EPEC strain but in none of the B2 members of the ExPEC phylogroup (Fig. 11). Interestingly, it is also found in three of the four (75%) AIEC strains (Fig. 11B). This means that four of the five InPEC members of the B2 phylogroup carry this allele, while being found in none of the 3 B2 commensals or 32 B2 ExPEC strains. These results suggest that *ehaA* and *ehaB* are important virulence factors for intestinal pathogenesis and suggests that *ehaB* may be an AIEC-associated virulence factor.

Another AIEC- or InPEC-specific finding in our results is the profound changes in *upaB*, which encodes an autotransporter that binds to fibronectin and glucosaminoglycans (166). It has been shown to promote uropathogenesis and colonization of the bladder in a mouse model (167). Our results suggest *upaB* is found widely throughout ExPEC and nonpathogenic B2 strains, with only 2 of 35 of these strains missing the gene (Fig. 11). However, the two commensal strains that carry this gene are from the ST73 and ST131 sequence types and appear to have lost virulence factors on their way to becoming nonpathogenic. In contrast to ExPEC B2 strains, all five B2 InPEC strains (four AIEC and one EPEC) lacked a similar *upaB* (Fig. 11B). In the AIEC strains, the *upaB* the gene contained either a premature stop codon (LF82 and UM146) or a 189-bp insertion (NC101 or NRG 857C), explaining why this potential connection has not been investigated. The B2 EPEC strain E2348_69 did not elicit even a partial hit.

Several autotransporters appear to be tightly linked in the ST131 group: *espC*_ST131_, *tsh*_ST131_, *upaG*_ST131_, and *upaH*_ST131_ (Fig. 11). Outside ST131 strains, these autotransporters only appear sporadically (Fig. 11). In fact, *espC*_ST131_ and *tsh*_ST131_ are only found in one strain outside ST131 strains in our pathotype database: CI5 (UPEC; B1) (Fig. 11). However, closer examination shows that this appears to be caused by remarkable diversity in the *upaG*_ST131_ and *upaH*_ST131_ genes. UpaH is a Va AIDA-I autotransporter that is involved in biofilm formation and colonization (168, 169). UpaG is a Vc trimeric autotransporter adhesin that has been shown to promote biofilm formation, adherence to host matrix, and abiotic surfaces (170). These genes are highly conserved, but alignments show a large degree of variation from sequence type to sequence type, mainly in the middle of the sequence. This has been noted in both genes (169, 171). To reflect this, *upaG* and *upaH* are annotated to indicate they used ST131 references. This could mean that UpaG and UpaH could be useful for quickly determining whether an isolate belongs to virulent sequence types (such as ST131 or ST73) using multiplex PCR. As a vaccine target, UpaG and UpaH may lack specificity, however, for UpaH conserved regions have been identified throughout phylogroups and work on a vaccine is under way (168, 169). The question remains as to whether UpaH is involved in colonization of other areas, since it is found in pathotypes (e.g., EHEC) that do not colonize the bladder.

Two SPATEs--EspC_ST131_ and Tsh_ST131_--are found almost exclusively in ST131 strains with the interesting exception of also being found in the CI5 strain (B1; UPEC) (172, 173) (Fig. 11A). However, while these were annotated as *espC* and *tsh* by the VFDB and some annotation software, it appears that this annotation may be incorrect. Using these references, we saw no hits in any of our EPEC strains or APEC strains, where research on EspC and Tsh has been done, respectively (27, 173–175) (Fig. 11). Closer examination revealed that *espC* from E2348/69 was only 60% identical to this reference, so a second *espC* (*espC*_E2348/69_) was included, while *tsh* from APEC strains were only ∼41% identical to *tsh*_ST131_. In fact, *tsh*_ST131_ appears to be related to *adcA* (CBG90828; 70% identical; 72% coverage) and *espC*_ST131_ is related to a putative autotransporter gene (CBG91787; 87% identical; 100% coverage) from Citrobacter rodentium (176–178). The *espC*_E2348/69_ is found on integrative element 5 (IE5), and the surrounding genes do not match those of *espC*_ST131_, while *tsh*_ST131_ is found only two genes upstream of *espC*_ST131_. The only strain carrying *espC*_E2348/69_ was the E2348/69 strain (Fig. 11). This is probably because E2348/69 is the only B2 EPEC strain in our database, and this gene appears to be isolated to LEE-containing B2 strains in our phylogroup database. This would explain the results by Mellies et al. (173), who only found these genes in a subset of EPEC strains. The fact that *tsh*_ST131_ and *espC*_ST131_ are specific for ST131s does make them potential vaccine targets, but more work must be done to determine whether the proteins they encode contribute to virulence and induce an antibody response.

**FIG 12:**
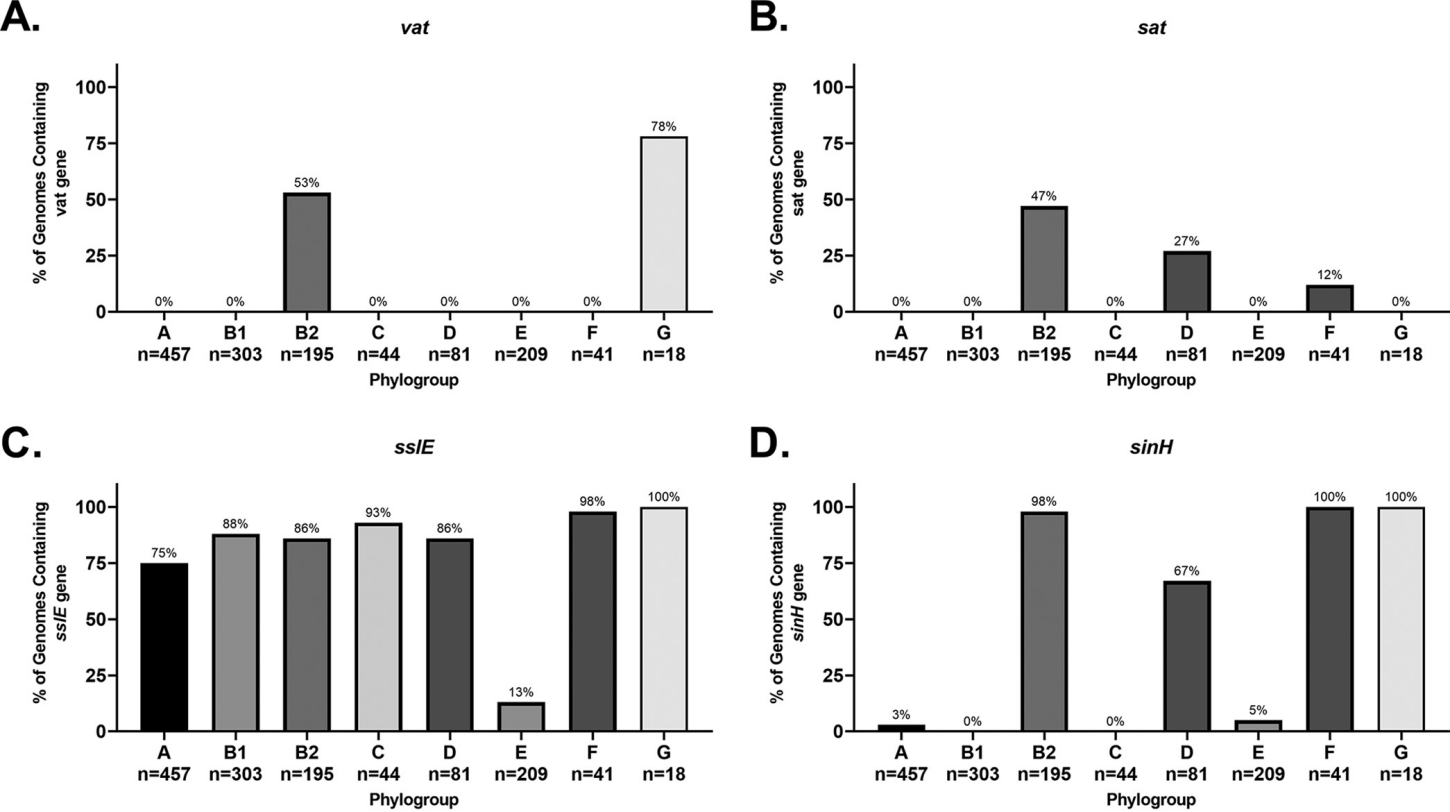
Phylogroup distribution of autotransporters. (A) Distribution of the gene encoding vacuolating autotransporter toxin (*vat*). (B) Distribution of the gene encoding secreted autotransporter toxin (*sat*). (C) Distribution of the gene encoding accessory colonization protein (*sslE*). (D) Distribution of the gene encoding invasion-like protein (*sinH*). Distributions were determined using megaBLAST to bin strains from each phylogroup into hit versus no hit.

Vacuolating autotransporter toxin (Vat) has been shown to contribute to uropathogenesis (179). Our data show that it is only found in the B2 and G phylogroups. It is found in the well-known ST73 and ST95, as well as lesser-known sequence types, but not ST131 strains. It should also be noted that sometimes this *vat* gene is mistakenly annotated as temperature-sensitive hemagglutinin, *tsh* (or the closely related hemoglobin-binding protease, *hbp*) or *sepA*, which encodes a *Shigella* virulence factor (180, 181). Of these genes, only *sepA* was found on the chromosome of one strain examined in our database, though *tsh/hbp* was found on the plasmid of 50% (4/8) of APEC strains, and *sepA* was found on the plasmid of all B1 EAEC and EAHEC strains. To verify each *vat* hit, we examined each hit for a *marA*/*papX* regulator (*vatX*) immediately downstream and the *yag* operon roughly 4 kb upstream (179). In our phylogroup database, *vat* is found exclusively in phylogroups B2 (53%; 103/195) and G (78%; 14/18) (Fig. 12A). Vat is a potential target, as it is found in many of the common ExPEC sequence types (ST73, ST95, and ST127), and it elicits an antibody response in UTI patients infected with *vat^+^* E. coli (179). The largest downside is that targeting Vat would not target ST131, a major cause of ExPEC infections worldwide.

Secreted autotransporter toxin (Sat) is another vacuolating cytotoxin implicated in uropathogenesis (182, 183). Like Vat, it also elicits a strong antibody response, but unlike *vat*, it is also found in ST131 and ExPEC strains from the D and F phylogroup in our pathotype database (Fig. 11) (183). It is also more widely distributed in our phylogroup database, where it is found in 47% of B2 strains (92/195), 27% of D strains (22/81), and 12% of F strains (5/41) (Fig. 12B). One interesting note is that *sat* is found in only one of the 457 strains in our phylogroup A database: VR50. While both *sat* and *vat* are only found in only barely 50% of B2 strains, 87% (170/195) of B2 strains carry one or the other (or both), making a polyvalent vaccine targeting both an intriguing prospect (Fig. 12B). Such a hypothetical vaccine would provide protection against 86% (30/35) of the ExPEC, UPEC, and NMEC strains in our pathotype database, while only targeting 11% (1/9) of the nonpathogenic strains.

The invasion-like autotransporter *fdeC* binds to human epithelial cells and contributes to colonization of kidneys and bladder in an animal model, and it may be protective as a vaccine target (184). In our pathotype database, the gene encoding the FdeC adhesin is found in nearly every strain (Fig. 11). It appears that certain alleles of *fdeC* may be associated with intestinal lifestyles, although these allelic trends appear to fall along A/B1/C/E and B2/D/F/G clusters (Fig. 11). However, the fact that *fdeC* genes are found throughout almost all strains makes the protein it encodes a less ideal vaccine target.

The accessory colonization factor encoded by *sslE* is a zinc-metalloprotease with mucinase activity (185). The *sslE* gene is found across all phylogroups but is mostly lacking the EHEC/STEC pathotype. This is even seen in EHEC/STEC strains that belong to the B1 phylogroup, despite B1 strains in other phylogroup still having this gene (Fig. 11). This trend is also seen in our phylogroup database (Fig. 12C). Roughly 75% of *stx*^+^ B1 strains carry *sslE* compared to 96% of B1 strains lacking *stx* (see Fig. S6). In the E phylogroup, only 1% of *stx^+^* strains carry *sslE* compared to 56% of *stx* mutant strains (see Fig. S6). This may indicate that *sslE* is either not required or selected against during EHEC infections.

Interestingly, inoculation with FdeC and SslE have been shown to be protective against UPECs (184, 186). However, our results show that both of these targets could produce significant off-target effects considering how broadly they are conserved in commensals (Fig. 11).

The *pet* and *pic* autotransporters are normally associated with EAEC strains (32). In our data set, *pet* is found almost exclusively on the chromosomes EAEC and EAHEC strain from the B1 phylogroup and, strangely, on the chromosome of ED1a (B2; commensal). However, it is truncated and predicted to be expressed in two parts (Fig. 11). The 042 (D; EAEC) strain lacks *pet* on its chromosome, but a divergent form annotated as *pet* and picked up by our reference *pet* is found on its plasmid (data not shown). The *pic* gene is associated with EAEC as well, and all six EAEC or EAHEC strains share a near identical allele—EAECs are 97% identical to the reference, while EAHECs are 96.9% identical. There is an allele of *pic* that is found in B2 strains, specifically those belonging to ST73 (Fig. 11). The only other instance is also the most divergent allele, which is found in the NC101 (AIEC; B2).

The invasin-like protein SinH appears to be strongly associated with ExPEC strains, but the distribution results from our phylogroup database suggests this association is more likely with the B2/D/F/G cluster than specifically with ExPEC strains (Fig. 11 and 12), although this does not rule out a contribution to virulence. Outside of ExPEC strains, B2 commensal strains also carry this gene, but in ED1a the beta-barrel domain that links the protein to the outer membrane is missing due to a premature stop codon (Fig. 11). There also appears to be an ST131-specific allele that is a relatively divergent (ca. 88 to 90% identical) from the *sinH* found in other ExPECs (see Fig. S7). There are two significant hits in the RM13514 (EHEC/STEC) and RM13516 (EHEC/STEC) strains, but the SinH in RM13514 is missing the beta-barrel domain, and the RM13516 strain is very divergent and aligns poorly with the other SinHs on the protein level (see Fig. S7). Our phylogroup database verifies the association with the B2 phylogroup, where it is found in 98% of strains, and shows that *sinH* is also strongly associated with the F and G phylogroups (100%) and, to a lesser extent, with the D phylogroup (48%) (Fig. 12).

### Other adhesins and miscellaneous virulence genes are either ubiquitous or enriched in B2 strains.

**FIG 13:**
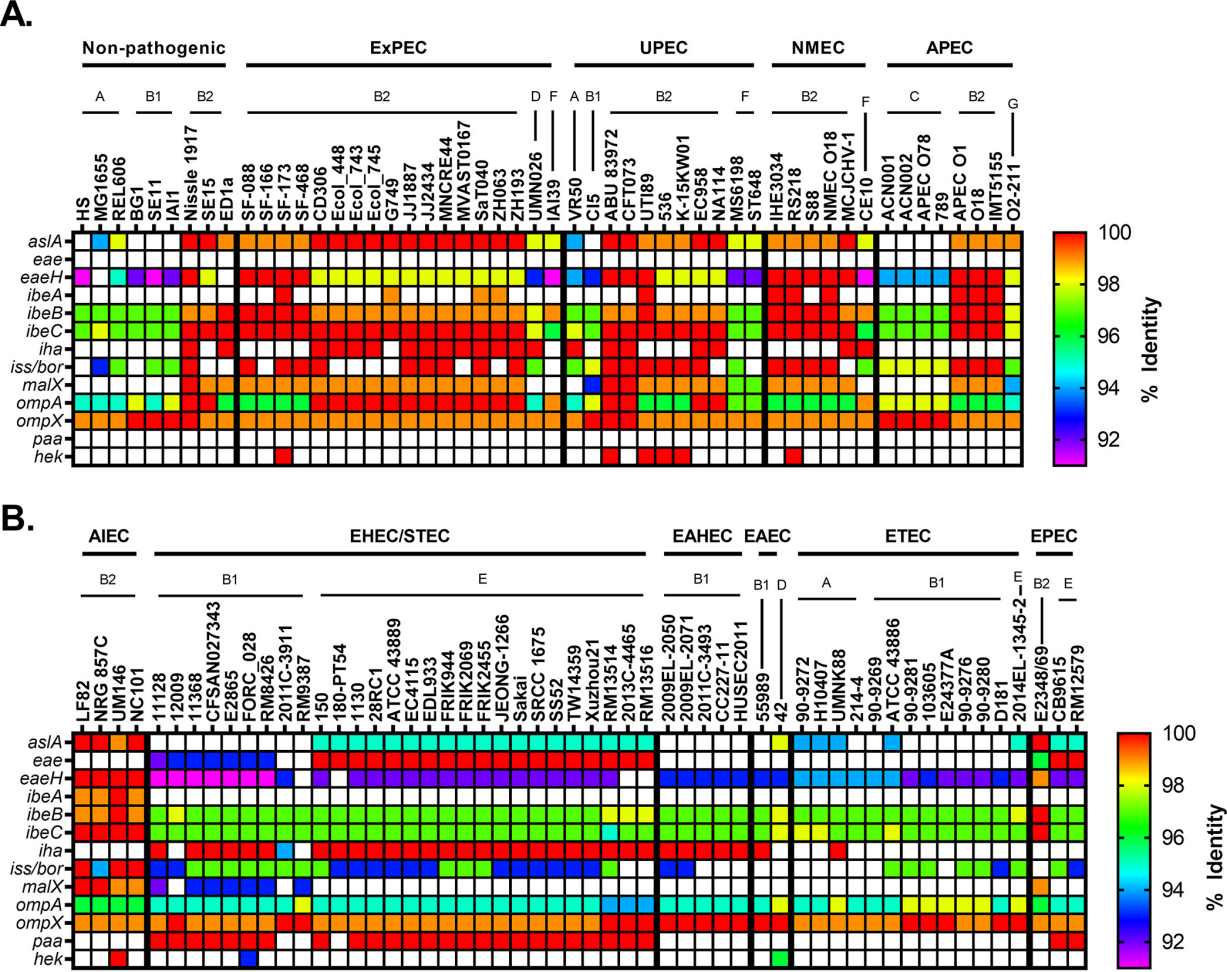
Pathotype distribution of other chromosomal virulence factors. (A and B) Percent identity results from megaBLAST alignments for nonpathogenic and ExPEC strains (A) and InPEC strains (B). The percent identity was determined using megaBLAST with the reference genes found in Data Set S1 in the supplemental material.

The locus of enterocyte effacement (LEE) carries a type 3 secretion system related to virulence, is known to be associated with EPEC and EHEC/STEC strains, and is responsible for their characteristic attaching-and-effacing (A/E) phenotype (187). The LEE-encoded attachment protein intimin is a product of *eae* (also called *eaeA*) and is responsible for EHEC and EPEC characteristic attachment phenotype, and our results are in agreement (Fig. 13). Another adhesin, EaeH, was first identified in ETEC. It is upregulated when ETEC strains interact with epithelial cells and promotes adhesion and toxin delivery (188, 189). However, it seems this adhesin is ubiquitous, since it is found in all but five strains, though there may be some allelic differences in *eaeH* (Fig. 13). Unlike most molecules that show allelic differences roughly along the A/B1/C/E and B2/D/F/G clades, *eaeH* appears to have a B2-specific allele.

Porcine A/E-associated protein (encoded by *paa*) is an LEE-encoded adhesin that is required for EHEC infections (190). It has been found to be more immunogenic than intimin, and it confers a slight protective effect against colonization of EHEC strains in mice when used as a vaccine (191). Our results shows that it is predominantly found in LEE-carrying pathotypes, though it appears to be missing in some B1 EHEC/STEC strains and the B2 EPEC strain (Fig. 13).

**FIG 14:**
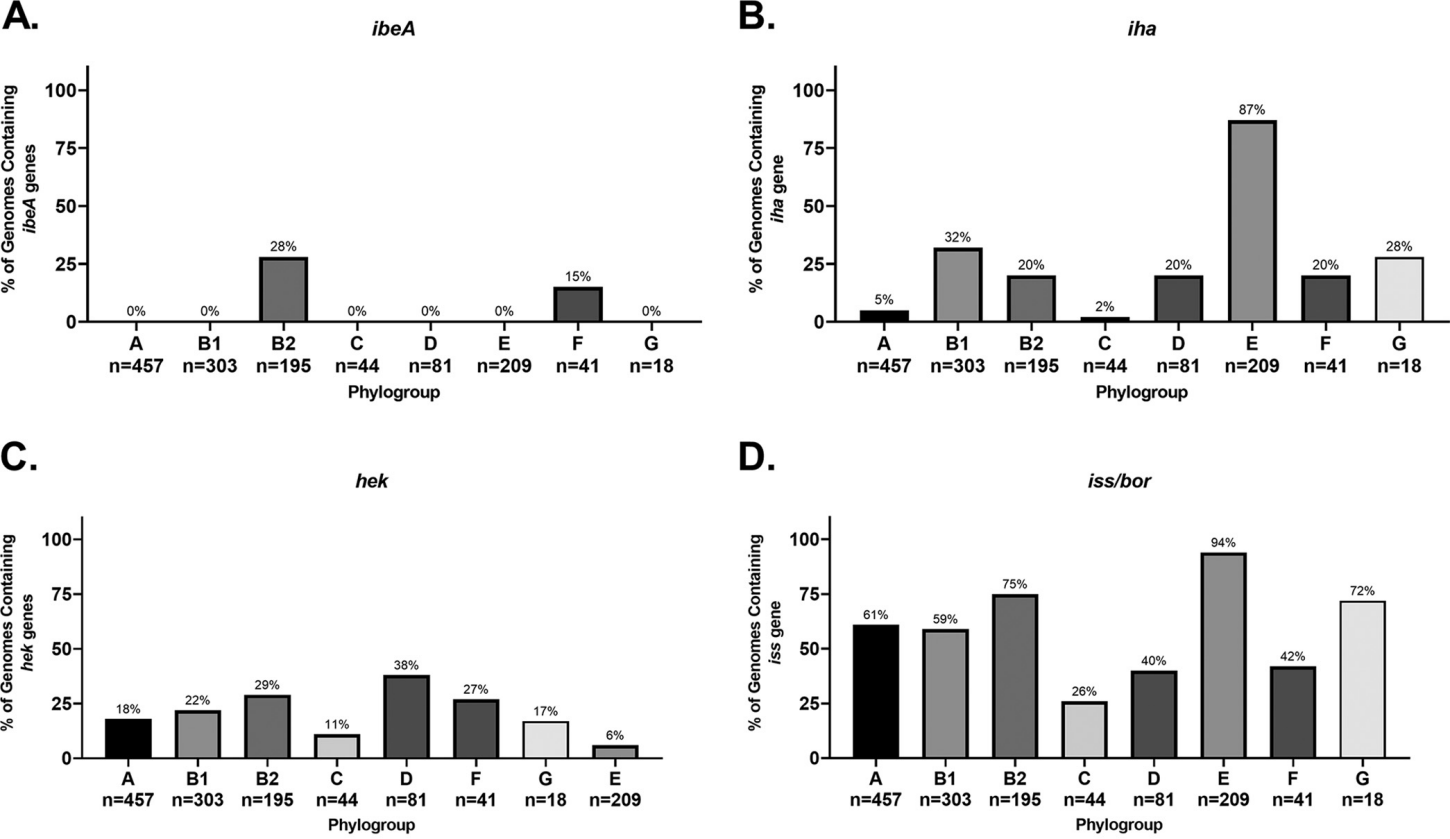
Phylogroup distribution of other virulence factors. (A) Distribution of the gene encoding invasion of brain endothelium A (*ibeA*). (B) Distribution of the gene encoding iron-regulated homologous adhesin (*iha*). (C) Distribution of the gene encoding the Hek adhesin (*hek*). (D) Distribution of the genes encoding increased serum survival protein or *bor* (*iss*/*bor*).

The invasion of brain endothelium A (*ibeA*) gene encodes a protein that has been shown to be important for invasion of the blood-brain barrier (192). It has also been shown to be an important virulence factor in APEC strains (192). Our pathotype results largely support these assertions, but the gene appears to only be found in B2 strains of these pathotypes: 3/5 B2 strains in NMEC and 3/3 B2 strains in APEC (Fig. 13). The F-phylogroup strain (CE10) and two of the B2 strains NMEC strains lacked *ibeA*, and none of the phylogroup C APEC strains carried the gene (Fig. 13). Interestingly, all four AIEC strains in our database contained *ibeA*, which supports recent work showing that this gene is important for intestinal colonization, cellular invasion, and macrophage survival of the NRG857 (AIEC) strain (Fig. 13B) (33). In our phylogroup database, *ibeA* is only found in phylogroups B2 and F, which may further explain why most AIEC strains are from the B2 phylogroup (Fig. 14A).

The *malX* gene encodes a phosphotransferase system II enzyme and is associated with ExPEC strains (193–195). It may also increase persistence in the intestines (156). Our results show that it highly conserved in B2 strains and, to a lesser degree, pathogenic B1 strains from the UPEC and EHEC/STEC pathotypes (Fig. 13).

The iron-regulated homologous adhesin encoded by *iha* gene is part of the PAI_CFT073_ I and PAI_536_ II pathogenicity islands and, unlike the P fimbriae, appears to be more widespread (107, 196). This suggests that *iha* has weaker selection against it or greater selection for it. Iha has been shown to be important for colonization of the kidneys and bladder in a mouse model of infection (196). In our work, it is found widely in B2 strains and pathogenic strains in general (Fig. 13). An interesting exception is that it is not found in either ST95 or ST127 B2 strains, two common sources of ExPEC infections. In EHEC/STEC strains, which are all from the B1 and E phylogroups, it appears highly conserved (Fig. 13). However, it is not found in any of the EPEC strains which may suggest that the acquisition of an *iha* PAI in addition to *stx* is important for EHEC/STEC pathogenesis and differentiates EHEC/STEC strains from EPEC strains (Fig. 13B). It is also lacking in B2 strains from the AIEC, NMEC, and APEC pathotypes, which may be related to their lifestyle (Fig. 13A). In our phylogroup database, it was found in a relatively diverse set of phylogroups and especially enriched in phylogroup E (Fig. 14B). One intriguing thing about targeting Iha is that, outside EHEC/STEC strains, the gene that encodes it is often found on the same pathogenicity island (e.g., PAI_536_ II) as *sat* and *iutA* (aerobactin), which have already been mentioned as potential vaccine targets. Targeting any of these alone has a serious drawback in that they are not present in the majority of the ST95 strains examined in our pathotype database. However, as with our speculations on combining Sat and Vat to cover the majority of ExPEC strains, Vat combined with either Iha or IutA appears to confer similar or greater coverage.

The Hek adhesin is encoded by a gene that is part of the PAI_536_ II pathogenicity island and is involved in autoaggregation, hemagglutination, heparin binding, adherence, and invasion of host cells in NMEC strains (103, 197, 198). Despite being found on the same pathogenicity island as *iutA*, *sat*, and *iha*, it has apparently been lost in most of those strains in our pathotype database (Fig. 13). It is only found sporadically in B2 strains, specifically ST127, and occasionally ST73 and ST95 strains. In our phylogroup database, it is found in low abundance in all phylogroups examined (Fig. 14C). The relatively low number of strains carrying this protein makes it less suitable as a vaccine target.

The prophage-encoded increased serum survival gene (*iss*) and *bor* genes have been associated with ExPEC infections for at least four decades. The proteins encoded by these genes are known to be surface exposed, have been shown to help E. coli resist the host complement system, and elicit an immune response in avian hosts (199–201). Since the proteins encoded by *iss* and *bor* are more than 90% identical, we have not distinguished between the two here (202). Both were first found on plasmids, but more recent work has shown that they are also carried on the chromosome (203). Our results suggest that *iss*/*bor* are widespread and found in all pathotypes (Fig. 13). There appears to be no phylogroup or sequence types trends, since these genes are found sporadically through different sequence types and are found often in all phylogroups (Fig. 13 and 14D). This is most likely because they are encoded by prophages and means that they are unlikely to be suitable vaccine targets.

### Commensalism and vaccine targets.

Our results show that the majority of virulence factors (VFs) and markers for extraintestinal pathogenesis are associated with the B2 phylogroup or the B2/D/F/G cluster. In many cases, these VFs are found exclusively (or nearly exclusively) in these phylogroups. In other cases, the VFs are found predominantly in the B2/D/F/G cluster but also in a minority of strains from other phylogroups. This suggests that the B2/D/F/G cluster is the dominant source of pathogenic strains, which is known, although estimates of the extent to which they are predominant vary. A similar conclusion with B2 and D strains was reached over 20 years ago in an experimental determination of the lethality of 82 strains from the E. coli reference (ECOR) collection cross-referenced against the presence of seven virulence determinants (204). There, researchers found that these virulence factors and lethality correlated with the B2 phylogroup (this was before phylogroups F and G were described). Our work not only verifies this but expands it to a wider range of virulence factors and, importantly, also shows that the B2/D/F/G cluster is the dominant reservoir for extraintestinal pathogenic virulence factors in general.

**FIG 15:**
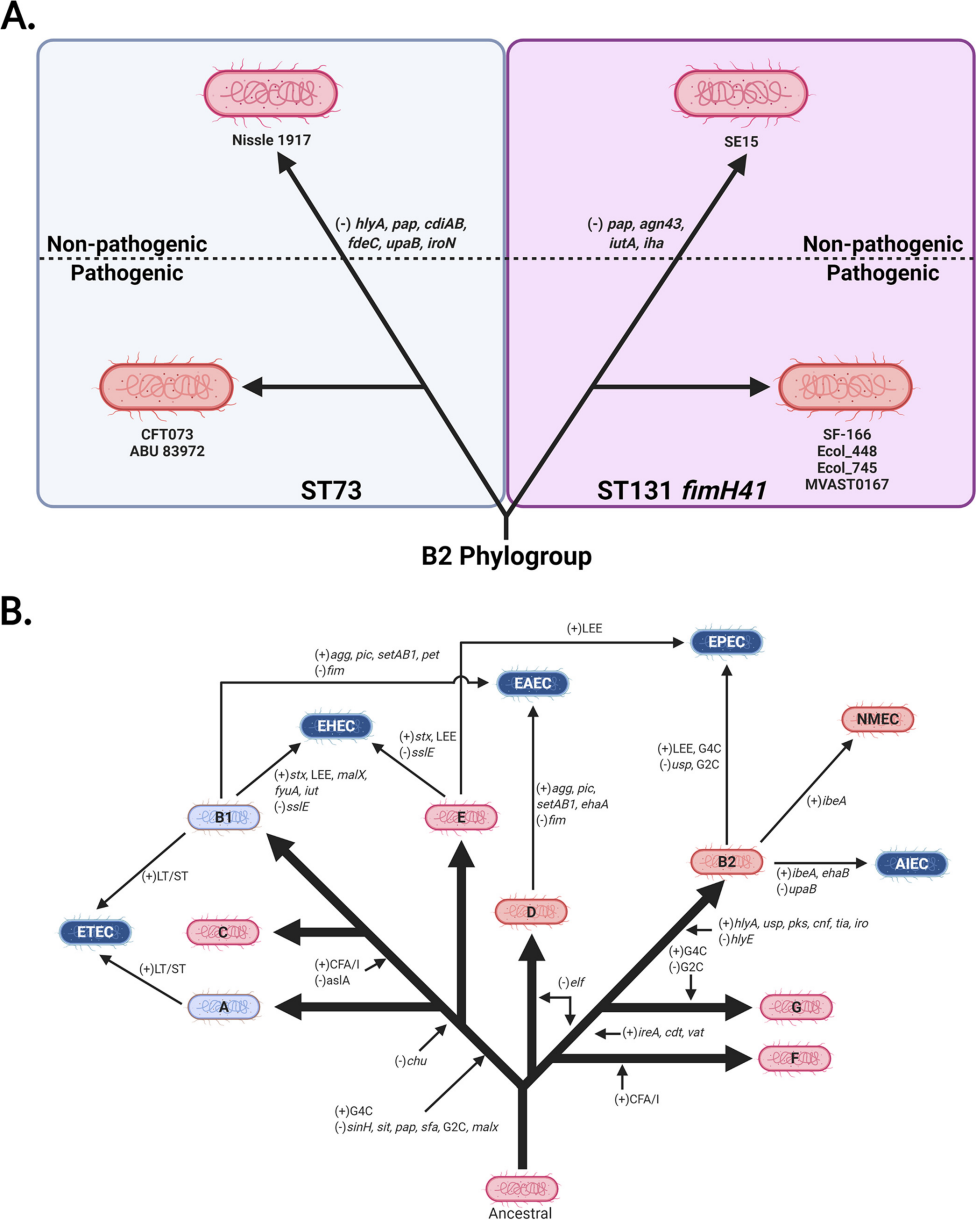
Genetic difference in pathogenicity. (A) Schematic for the loss of pathogenicity in ST73 and ST131 using examples from strains examined in the present study. (B) Gain and loss of virulence factors at various points in E. coli phylogroups. Labels were assigned under the presumption that gaining genetic factors are rare, while loss is common, i.e., the map was built to reflect the lowest gain of genes possible based on the results presented here. ExPEC, extraintestinal pathogenic E. coli; UPEC, uropathogenic E. coli; NMEC, neonatal meningitis-causing E. coli; APEC, avian pathogenic E. coli; InPEC, intestinal pathogenic E. coli; AIEC, adherent-invasive E. coli; EHEC, enterohemorrhagic E. coli; EAEC, enteroaggregative E. coli; ETEC, enterotoxigenic E. coli; EPEC, enteropathogenic E. coli. Light blue, commensals (or those likely to be commensal); dark blue are InPEC strains; red, ExPEC strains; gray, phylogroups of uncertain intrinsic virulence. This illustration was made using BioRender.

A major implication of this is that B2/D/F/G isolates that contain virulence factors should be looked at with suspicion but, without a comprehensive and in-depth overview of virulence factors, it is hard to say whether or not a strain from this cluster can cause disease. This is easy to see with the three nonpathogenic B2 strains in our pathotype database: Nissle 1917, SE15, and ED1a. Nissle 1917 is an ST73 strain, like ABU 83972 (ABU) and CFT073 (UPEC), and SE15 belongs to ST131, of which we have numerous examples of in our pathotype database. In most cases, these commensal strains contain the same virulence factors as pathogenic B2 strains, with some notable exceptions. Compared to other ST73 strains, Nissle 1917 is missing *hlyA* (toxin), full-length *pap* (fimbriae), *cdiAB* (autotransporter), *fdeC* (autotransporter), *upaB* (autotransporter), and salmochelin (iron acquisition). With ST131 strains of the *fimH41* subclass, SE15 has lost the partial *pap* (fimbria) genes seen in other ST131 strains, as well as *agn43* (autotransporter), *sat* (autotransporter), aerobactin/*iutA* (iron acquisition), and *iha* (adhesion/iron acquisition)—all proteins found on PAI_CFT073_ I (Fig. 15A). The loss of these genes appears to abolish pathogenicity in a strain from a highly pathogenic sequence type. It is important to note that most cursory overviews of these B2 commensals would conclude that they have high pathogenic potential, especially if they were compared to strains outside their phylogroup or sequence type. For example, Nissle 1917, still carries *kpsTM*, K5 biosynthetic genes, *pic*, *sat*, *vat*, *foc* fimbriae, *papA*, *fyuA* (yersiniabactin), *iutA* (aerobactin), *ireA*, *iha*, *malX*, and *iss*. It also still carries the *pks* genotoxin island (205). It can be reasonably concluded that this is a virulence fingerprint that is from a strain with high probability of causing disease, and yet Nissle 1917 has been used as a probiotic to successfully treat intestinal diseases for a century (206).

Another implication from our work is that most of the ExPEC-associated VFs are found widely and predominantly in B2 and D strains (Fig. 15B). This suggest that strains from the A/B1/C cluster should be viewed with greater suspicion when clinical isolates are shown to contain virulence factors that are generally found in the B2/D/F/G cluster. This is because for these strains to have these genes, they almost certainly had to acquire them through horizontal gene transfer, and then the gene had to most likely be selected for to fix it in the population. With a gene that confers an advantage during infection, increased virulence is the simplest mechanism of fixation.

However, pathotype associations from this work need to be interpreted with care, since several pathotypes we catalogued have few representative strains and are therefore at greater risk of selection bias. There is, of course, the same risk of bias with our phylogroup associations, but we feel it is much less likely because of the much larger sample size and because our phylogroup database comprises all complete E. coli genomes currently available in NCBI’s RefSeq database.

Our work also suggests that vaccines targeting VFs will likely target many strains in the B2/D/F/G cluster, even nonpathogenic ones. Although it has been postulated that an ideal vaccine candidate would only target pathogenic strains, targeting all B2/D/F/G strains is not necessarily a dead end (13). B2 strains are now common commensals in the developed world, but much rarer in older data sets and in the developing world, and there is no indication that the lack of B2 strains in the gut microbiota has been detrimental to health (207, 208). In fact, the opposite may be true since microbiota in developed countries tend to be less diverse (209). Accepting this fact leads to the obvious conclusion that specifically targeting B2 strains may open new avenues for vaccine research because while we have struggled to clearly define the ExPEC pathotypes, phylogroups can be clearly delineated. A successful B2 or B2/D/F/G cluster vaccine could potentially eliminate a large source of both virulent strains and a virulence factor reservoir.

**FIG 16:**
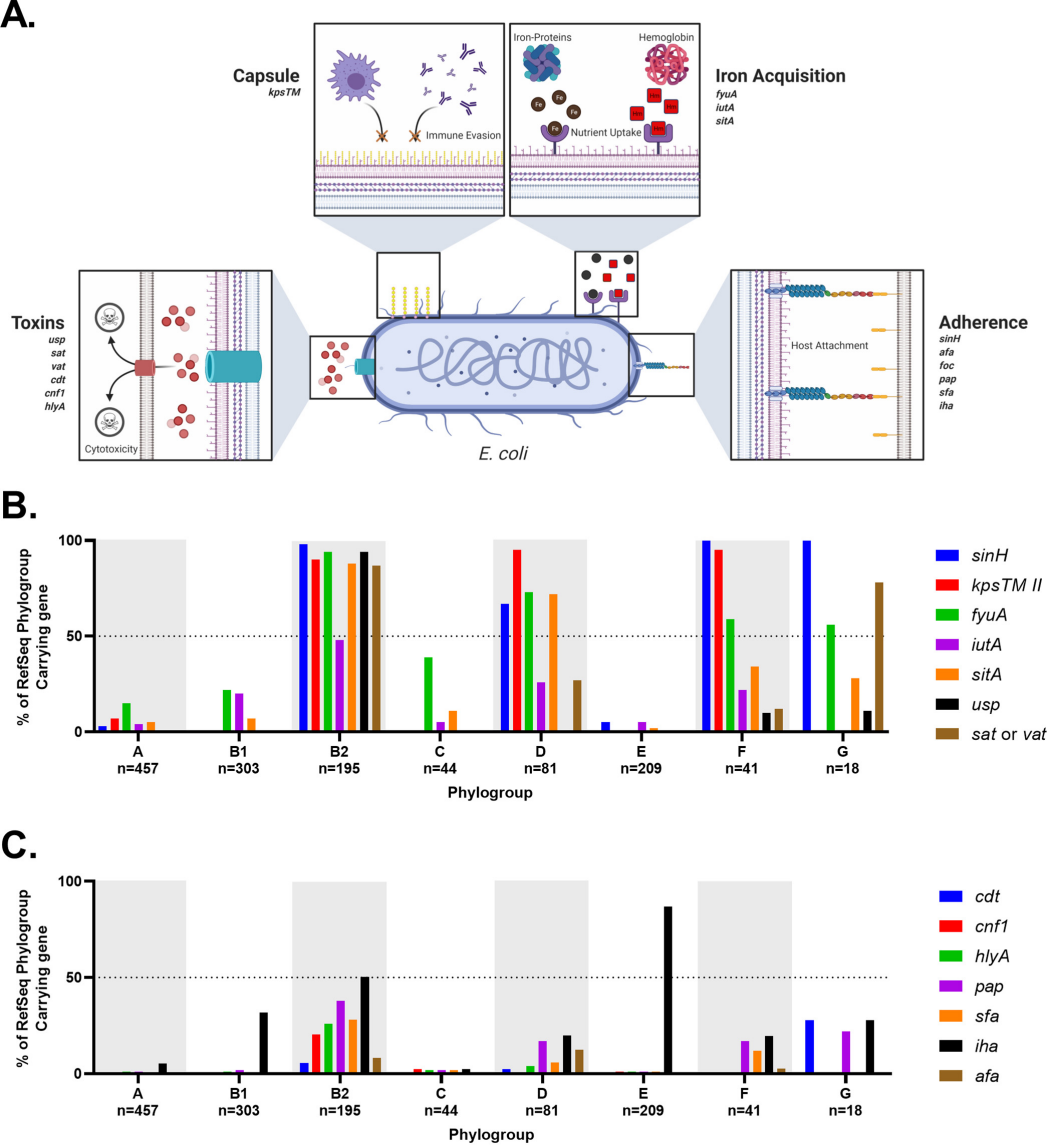
Potential polyvalent vaccine targets. Phylogroup distributions of potential targets were determined by using megaBLAST to cross reference all 1,348 complete E. coli chromosomes in the RefSeq database that could be categorized by phylogroup against potential targets. (A) Schematic overview of targets and their virulence mechanisms. Made using Biorender. (B) Targets with high disease impact and wide distributions through ExPEC-associated phylogroups; (C) targets with limited distribution, but still high disease impact. Terms: uropathogenic protein, *usp*; *Salmonella*-like invasion H, *sinH*; secreted autotransporter toxin, *sat*; vacuolating autotransporter toxin, *vat*; G2C-specific *Kapsel* polysaccharide genes M and T, *kpsTMII*; ferric yersiniabactin uptake receptor, *fyuA*; aerobactin uptake receptor, *iutA*; iron/manganese transporter, *sitA*; cytolethal distending toxin, *cdt*; cytotoxic necrotizing factor-1, *cnf1*; α-hemolysin, *hlyA*; afimbrial adhesin, *afa*; F1C fimbriae, *foc*; P fimbriae, *pap*; S fimbriae, *sfa*; IrgA homologue adhesin, *iha*.

Along these lines, the phylogroup distribution of what we believe are the vaccine targets with the most potential given their prevalence in different pathotypes and phylogroups is shown in Fig. 16. These potential targets are predominantly found on the surface, but it should be noted that the proposed toxin targets are excreted. While many successful vaccines against other organisms have targeted secreted toxins, the multifaceted nature of E. coli virulence means that a monovalent toxin vaccine may reduce the severity of the disease rather than completely prevent an infection. However, a polyvalent vaccine targeting several of these proposed proteins—including toxins—would target multiple mechanisms of virulence such as adherence or immune evasion. This would increase the chances that an infection could be completely prevented rather than simply abated. Such an approach would also make developing resistance while maintaining virulence difficult (Fig. 16A). Targeting uropathogenic-specific protein (Usp), yersiniabactin receptor (FyuA), group 2 capsule (KpsTM), vacuolating toxins (Sat/Vat), and iron/manganese transporter (SitA) would target the vast majority of strains from the B2 and D phylogroups—which are more intrinsically virulent. They would do this while avoiding strains from phylogroups more common among commensal unless those strains have acquired these virulence factors (e.g., strain VR50) (Fig. 16B). Other, less widespread targets such as α-hemolysin (HlyA), afimbrial adhesin (Afa), F1C fimbriae (Foc), P fimbriae (Pap), S fimbriae (Sfa), and aerobactin receptor (IutA) make attractive auxiliary targets because even though they are rare, they have been shown to play a major role in pathogenicity (Fig. 16C).

This information is being used to guide polyvalent vaccine development in our lab, but these genes and their distribution should also be useful when determining the potential pathogenicity of isolates, as well as in further elucidating the pathogenicity of E. coli.

## MATERIALS AND METHODS

### Comparative genomics.

Alignments of virulence factors and strains were created using megaBLAST plugin for Geneious Prime 2019.2.3 using a 1/–2 match/mismatch scoring matrix, linear gap score, and a maximum E value of 1E–10. Where necessary, a culling limit of 1 was used to prevent too many off-target hits. Percent identity outputs were exported as a spreadsheet and used to produce heatmaps in GraphPad Prism version 9.0.0 for Windows (GraphPad Software, San Diego, CA).

Virulence factors database was created using the Virulence Factor Database, Victors, and PATRIC virulence databases as a backbone and expanded upon with literature (56–58). Strain and phylogroup databases were constructed as described below. Virulence factors and sequences can be found in Data Set S1 in the supplemental material.

### RefSeq phylogroup database construction.

Escherichia coli strains for the phylogroup database were retrieved from NCBI’s RefSeq database (accessed 1 September 2020) with the following query string: Search all[filter] AND “Escherichia coli”[ORGN] AND latest[filter] AND “complete genome”[filter] AND (all[filter] NOT “derived from surveillance project”[filter] AND all[filter] NOT anomalous[filter]) Sort by: SORTORDER. The 4,105 strains were trimmed to 1,351 strains above 3.5 megabases. These strains were classified by phylogroup using in-house methods (see Appendix S1 in the supplemental material). Briefly, megaBLAST was used to align short sequences derived from Clermont phylotyping fragments. Strains were binned based on hits or no hits to each fragment (61). The resulting pattern of “hit/no hit” for each of the main four fragments were used to determine phylogroup (see the Fig. S1 legend for more information). Phylogroups E, C, and G strains were classified next based on the presence of the *arpA_gpE*, *trpA_gpC*, and *ygbD_G* PCR fragments, respectively (55, 61, 210). For more information, see Appendix S1 in the supplemental material. A breakdown of strains in our phylogroup data can be found in Fig. S8. Accession numbers for strains divided by phylogroup can be found in Data Set S2 in the supplemental material.

### Strain curation.

Phylogroup and pathotype breakdown of strains in our pathotype database can be found in Fig. S9 in the supplemental material.

The extraintestinal pathogenic E. coli strains (ExPECs) refer to several pathotypes that cause disease outside the intestines; the major pathotypes are uropathogenic E. coli (UPEC), neonatal meningitis E. coli (NMEC), and avian pathogenic E. coli (APEC) (25). There are dozens of virulence factors said to be associated with ExPEC strains, though our results suggest that most of these may actually be found in the majority of strains (25). In the present study, ExPEC strains are labeled as ExPECs unless there is published evidence for the strains to be sorted into the UPEC, NMEC, or APEC pathotypes, even if a strain was collected from the urine of an infected patient (Table 2). This may mostly affect UPEC strains, since the terms ExPEC and UPEC are often used interchangeably. Of the 18 general ExPEC strains, 16 belong to the B2 phylogroup, 1 belongs to the D phylogroup, and 1 belongs to the F phylogroup. Of these strains, 12 belong to the ST131 sequence type. Given the highly virulent nature of ST131 strains, they were all considered ExPECs, with three exceptions: SE15, EC958, and NA114. SE15 was isolated from a healthy adult and shown to lack virulence and also lacks many virulence genes seen in other ExPEC strains, so it is classified as nonpathogenic (Table 1) (211). EC958 and NA114 were classified as UPEC strains because they were isolated in urine and carried UPEC-associated virulence factors not seen in other ExPECs: the *selC* genomic island in EC958 and the full-length *pap* fimbriae in NA114 (212, 213).

**TABLE 2:** Strains, accession numbers, phylogroups, STs, antigens, and references

Pathotype	Strain	Accession no.	Isolation source	Phylogroup	ST	O antigen	H antigen	K antigen^*a*^	FimH type^*b*^	Reference(s)
Nonpathogenic	HS	CP000802	Feces, healthy subject	A	ST46	O9			35	9
	MG1655	CP014225	Feces, healthy subject	A	ST10	O16	H48		27	227
	REL606	CP000819	Lab strain	A	ST93	O7			427/608	228
	BG1	MOAH00000000	Bovine intestines	B1	ST58	O159	H21		53	229
	SE11	AP009240	Feces, healthy subject	B1	ST156	O152	H28		38	230
	IAI1	CU928160	Feces, healthy subject	B1	ST1128	O8	H4		32	231
	Nissle 1917	CP007799	Feces, healthy subject	B2	ST73	O6	H1	K5	30	232
	SE15	AP009378	Feces, health subject	B2	ST131	O150	H5	K+	41	233
	ED1a	CU928162	Feces, healthy subject	B2	ST452	O81	H27	K+	225/580	233, 234
ExPEC	SF-088	CP012635	Bloodstream infection	B2	ST95	O1	H7	K1	30	235
	SF-166	CP012633	Bloodstream infection	B2	ST95	O1	H7	K1	41	235
	SF-173	CP012631	Bloodstream infection	B2	ST95	O18	H7	K1	18	235
	SF-468	CP012625	Bloodstream infection	B2	ST95	O25	H4	K1	27	235
	CD306	CP013831	Feces, cat	B2	ST131	O25	H4	K5	30	236
	Ecol_448	CP015076	Infected urine	B2	ST131	O16	H5	K100?	41	237, 238
	Ecol_743	CP015069	Infected urine	B2	ST131	O16	H5	K+	1426	237, 238
	Ecol_745	CP015074	Infected urine	B2	ST131	OR	H5	K100?	41	237, 238
	G749	CP014488		B2	ST131	O25	H4	K1	22	236
	JJ1887	CP014316	Recurrent cystitis	B2	ST131	O25	H4	K14	30	239
	JJ2434	CP013835		B2	ST131	O25	H4	K5	30	236
	MNCRE44	CP010876	Sputum	B2	ST131	O25	H4	K+	30	240
	MVAST0167	CP014492		B2	ST131	O16	H5	K100?	41	236
	SaT040	CP014495		B2	ST131	O25	H4	K5	22	236
	ZH063	CP014522		B2	ST131	O25	H4	K5	22	236
	ZH193	CP014497		B2	ST131	O25	H4	K5	30	236
	UMN026	CU928163	Urine, acute cystitis	D	ST597	O17	H18	K52	27	241
	IAI39	CU928164	Infected urine	F	ST62	O7	H45	K1	44	233
UPEC	VR50	CP011134	Urine, ABU	A	ST10	OR	H-	K1	27	93, 242
	CI5	CP011018	Pyelonephritis	B1	ST5082	O-	H14		1155	243
	ABU 83972	CP001671	Urine, ABU	B2	ST73	O25	H1	K5	12	244
	CFT073	AE014075	Blood, Pyelonephritis	B2	ST73	O6	H1	K2	10	245, 246
	UTI89	CP000243	Urine, cystitis	B2	ST95	O18	H7	K1	18	247, 248
	536	CP000247	Pyelonephritis	B2	ST127	O6	H31	K15	1467	249
	K-15KW01	CP016358	Fecal, ABU	B2	ST127	O6	H100	K+	310	250
	EC958	HG941718	Infected urine	B2	ST131	O25b	H4	K100	30	212
	NA114	CP002797	urine, UTI	B2	ST131	O25	H4	K+	30	213
	MS6198	CP015834	Urine	F	ST648	O1	H6	K+	27	251
	ST648	CP008697	Pleural effusion	F	ST648	O51	H4	K+	18	
NMEC	IHE3034	CP001969		B2	ST95	O18	H7	K1	18	186, 252
	RS218	CP007149	Cerebrospinal fluid	B2	ST95	O17	H7	K1	18	253–255
	S88	CU928161	Cerebrospinal fluid	B2	ST95	O45	H7	K1	54	127
	NMEC O18	CP007275	Cerebrospinal fluid	B2	ST416	O18	H7	K1	244	194, 256, 257
	MCJCHV-1	CP030111	Cerebrospinal fluid	B2	ST1193	O75	H5	K1	64	258, 259
	CE10	CP003034	Cerebrospinal fluid	F	ST62	O7		K1	44	260
APEC	ACN001	CP007442	Liver, chicken	C	ST23	O78	H9		35	261
	ACN002	CP007491		C	ST23	O79	H9		35	
	APEC O78	CP004009	Lung, turkey	C	ST23	O79			35	262
	789	CP010315	Avian colisepticemia	C	ST88	O78	H19		27	263
	APEC O1	CP000468	Colibacillosis, turkey	B2	ST95	O1	H7	K1	15	264
	O18	CP006830	Pericardium/lung, chicken	B2	ST95	O18	H7	K1	15	265
	O2-211	CP006834	Air sack, chicken	G	ST117	O2	H4		97	266
	IMT5155	CP005930	Colisepticemia, chicken	B2	ST140	O2	H5	K1	15	267, 268
AIEC	LF82	CU651637	Ileum, Crohn’s disease	B2	ST135	O83	H1	K+	436	269
	NRG 857C	CP001855	Ileum, Crohn’s disease	B2	ST135	O83	H1	K+	2	270
	UM146	CP002167	Ileum, Crohn’s disease	B2	ST643	O18	H7	K1	18	271
	NC101	AEFA01000000	Feces, mouse	B2	ST998	O2	H6	K1	1477	272, 273
EHEC/STEC	11128	AP010960	Bloody diarrhea	B1	ST16	O111	H-		86	274
	12009	AP010958	Bloody diarrhea	B1	ST17	O103	H2		25	274
	11368	AP010953	Bloody diarrhea	B1	ST21	O26	H11		440	274
	CFSAN027343	CP037943	Stool	B1	ST21	O26	H11		440	275
	E2865	AP018808	Cattle	B1	ST21	O26	H11		440	
	FORC_028	CP012693	Stool	B1	ST21	O26	H11		440	
	RM8426	CP028116	Creek	B1	ST21	O26	H11		440	
	2011C-3911	CP015240	Stool	B1	ST1727	O79	H7		31	276
	RM9387	CP009104	Feces, cattle	B1	ST2773	O104	H7		32	277
	150	CP028592	Cattle	E	ST11	O157	H7		82	
	180-PT54	CP015832	Outbreak isolate	E	ST11	O157	H7		82	278
	1130	CP017434	Cattle hide	E	ST11	O157			82	
	28RC1	CP015020	Bovine carcass	E	ST11	O157	H7		82	279, 280
	ATCC 43889	CP015854	Feces, HUS	E	ST11	O157	H7		82	281, 282
	EC4115	CP001164	Spinach outbreak	E	ST11	O157	H7		82	283
	EDL933	CP008957	Ground beef	E	ST11	O157	H7		36	284
	FRIK944	CP016625	Calf feces	E	ST11	O157	H7		82	
	FRIK2069	CP015846	Feces	E	ST11	O157	H7		82	
	FRIK2455	CP015844	Feces, steer	E	ST11	O157	H7		82	285, 286
	JEONG-1266	CP014314	Feces, steer	E	ST11	O157	H7		82	287
	Sakai	BA000007	Stool	E	ST11	O157	H7		36	288, 289
	SRCC 1675	CP015023	Apple cider	E	ST11	O157	H7		36	279, 280
	SS52	CP010304	Feces, cattle	E	ST11	O157	H7		82	290
	TW14359	CP001368	Spinach outbreak	E	ST11	O157	H7		82	291
	Xuzhou21	CP001925	Feces	E	ST11	O157	H7		36	292
	RM13514	CP006027	Lettuce outbreak	E	ST32	O145	H28		331	293
	2013C-4465	CP015241	Stool	E	ST335	O55	H7		82	
	RM13516	CP006262	Ice cream outbreak	E	ST6130	O145	H28		54	293
EAHEC	2009EL-2050	CP003297	Bloody diarrhea	B1	ST678	O104	H4			294
	2009EL-2071	CP003301	Bloody diarrhea	B1	ST678	O104	H4			294
	2011C-3493	CP003289	Stool, HUS	B1	ST678	O104	H4			295
	CC227-11	CP011331	Bloody diarrhea	B1	ST678	O104	H4			296
	HUSEC2011	HF572917	Stool, HUS	B1	ST678	O104	H4			
EAEC	55989	CU928145	Watery diarrhea	B1	ST678	O104	H4			233
	042	FN554766	Diarrhea	D	ST414	O44	H18	K+		91
ETEC	90-9272	CP024239	Diarrhea	A	ST48	O15	H11		41	297
	H10407	FN649414	Diarrhea	A	ST48	O78	H11	K80?	41	120, 298
	UMNK88	CP002729	Porcine neonatal diarrhea	A	ST100	O149	H10			299
	214-4	CP025840	Stool	A	ST398	Nontypable			25	300
	90-9269	CP024661	Diarrhea	B1	ST761*	OUND	H4			297
	ATCC 43886	CP024256	Feces	B1	ST1312	O25	H16		198	301
	90-9281	CP024243	Diarrhea	B1	ST58	O128	H27			297
	103605	CP025920	Stool	B1	ST443	O115	H5		24	
	E24377A	CP000800	Diarrhea	B1	ST1132	O139	H28		54	9, 302, 303
	90-9276	CP024299	Diarrhea	B1	ST5305	O114	H49		32	297
	90-9280	CP024240	Diarrhea	B1	ST5305	O114	H49		32	297
	D181	CP024252	Diarrhea	B1	ST4493	O182	H21		24	304
	2014EL-1345-2	CP024223		E	ST182	O169	H41		30	305
EPEC	E2348/69	FM180568	Diarrhea	B2	ST15	O127	H6		57	306
	CB9615	CP001846	Diarrhea, infant	E	ST335	O55	H7		82	307
	RM12579	CP003109	Urine	E	ST335	O55	H7		82	308

a K+ indicates a strain that contained *kps* genes without additional information. If the *kps* genes were identical to those for strains that had experimentally determined K-types or if the K-type was described with some uncertainty, the K-type is followed by a “?” (for example “K100?”).

b FimH types were determined using CGE’s FimTyper (226).

UPECs are characterized by their ability to multiply intracellularly in the urinary tract (25, 214). Many of the virulence factors described for this pathotype are represented in this work because UPECs are among the most studied pathogens to date. The loss of some virulence factors leads to asymptomatic bacteriuria (ABU). Our strain collection has 13 strains specifically labeled UPEC strains; two of these are known to cause ABU (83972 and VR50). The UPEC strains represented here belong to five different phylogroups and eight sequence types.

Strains that belong to the NMEC phylogroup can survive in the bloodstream and invade the meninges of infants (25). NMEC strains are generally considered to be difficult to distinguish from commensals (215).

The avian pathogenic E. coli (APEC) causes colibacillosis in poultry and other avian species and has many of the same virulence factors that are found in ExPEC strains (26).

Four strains belong to the adherent-invasive E. coli (AIEC) pathotype. This pathotype was first described in 1999 with the LF82 strain isolated from the ileal mucosa of a Crohn’s disease (CD) patient (216). AIECs have since been associated with inflammatory bowel disease, are characterized by the ability to invade intestinal epithelial cells and replicate in macrophages, and have been found to bind receptors overexpressed in CD patients using type I fimbriae (29, 86, 217). There have been no unique genetic factors described for this pathotype, other than a weak association with certain alleles of *chiA* chitinase, a gene encoding a protein shown to promote AIEC adherence to IEC (1, 216, 218). The four complete AIEC genomes here all belong to the B2 phylogroup, the phylogroup to which the majority of isolated AIEC strains belong.

Shiga-toxin-producing E. coli (STEC) strains have acquired the phage-associated Stx toxin (34, 39, 219). This pathotype is a foodborne illness that can produce a wide range of symptoms from asymptomatic to life-threatening hemorrhagic colitis and hemolytic-uremic syndrome (HUS). Technically, pathogenic STEC strains that have the ability to cause disease in humans are called enterohemorrhagic E. coli (EHEC) and belong predominantly to seven serotypes, including the well-known O157:H7 serotype. In practice, however, the terms STEC and EHEC are used somewhat interchangeably, along with verotoxigenic E. coli (VTEC). In addition to the Stx toxin, the most prominent virulence factor found in these strains is the LEE pathogenicity island, which encodes intimin among other things (34). Other described virulence genes in the literature include *espP*, *lpf*, *efa*, *toxB*, *eibG*, *ehaA*, *ompA*, *iha*, and *paa* (32).

Enteroaggregative E. coli (EAEC) is an emerging diarrheagenic pathotype that is characterized by its “stacked-brick” adherence pattern on Hep-2 cells. This pathotype is a serious cause of childhood diarrhea in developing countries and traveler’s diarrhea (35). EAEC can be asymptomatic, but during symptomatic infections the most common symptoms are watery diarrhea, abdominal pain, nausea, and vomiting (220).

During an outbreak of bloody diarrhea in Europe in 2011, a new hybrid pathotype called enteroaggregative hemorrhagic E. coli (EAHEC) was described (37). All known members of this pathotype are EAEC strains of the O104:H4 serotype that have acquired the Stx phage (221).

Enterotoxigenic E. coli (ETEC) is a pathotype that gets its name for the plasmid-transmitted heat-labile enterotoxin (LT) and heat-stable enterotoxin (ST). These strains can carry one or both toxins. LT is similar to cholera toxin and has a similar mechanism of action. ST is a much smaller peptide that binds to guanylate cyclase to increase cyclic GMP in the small intestines. ETEC is the most common cause of E. coli diarrheagenic disease and the leading cause of traveler’s diarrhea. It is also a major cause of acute infectious diarrhea, which accounts for an estimated 20% of all childhood deaths (222).

Enteropathogenic E. coli (EPEC) is another major diarrheagenic E. coli. This pathotype is characterized by the formation of A/E lesions. The attachment to intestinal cells is mediated by intimin, encoded by *eae* found in the LEE pathogenicity island (40).

Of these strains, 6 were nonpathogenic strains, 2 were lab-derived, 4 were AIEC, 29 were ExPEC/UPEC, 6 were NMEC, 8 were APEC, 28 were EHEC/STEC, 5 were EAHEC, 2 were EAEC, 13 were ETEC, and 3 were EPEC strains. For Clermont phylogroups, 8 belonged to the A phylogroup, 27 to the B1 phylogroup, 39 to the B2 phylogroup, 4 to the C phylogroup, 2 to the D phylogroup, 22 to the E phylogroup, 4 to the F phylogroup, and 1 to the G phylogroup. Table 2 contains a complete list of strains in our pathotype database. Figure S5 shows the pathotype and phylogroup distributions.

The sequence type of each strain was determined using Center for Genomic Epidemiology (CGE) MLST 2.0 software (223, 224). For strains where the O and H serotypes were not known, CGE SeroTypeFinder software was used (225). K-types were listed if they had been experimentally determined or if *neu* (for K1) or *kfi* (for K5) operons were present. When the strain contained *kps* genes without additional information, “K+” is listed in Table 2. If the *kps* genes were identical to strains that had experimentally determined K-types or if the K-type was described with some uncertainty, the K-type was listed followed by a “?” (for example “K100?”). FimH type was determined using CGE’s FimTyper (226).

## References

[1] Kaper JB, Nataro JP, Mobley HLT. 2004. Pathogenic *Escherichia coli*. Nat Rev Microbiol 2:123–140. 10.1038/nrmicro818. 15040260

[2] Diard M, Garry L, Selva M, Mosser T, Denamur E, Matic I. 2010. Pathogenicity-associated islands in extraintestinal pathogenic *Escherichia coli* are fitness elements involved in intestinal colonization. J Bacteriol 192:4885–4893. 10.1128/JB.00804-10. 20656906 PMC2944530

[3] Abram K, Udaondo Z, Bleker C, Wanchai V, Wassenaar TM, Robeson MS, Ussery DW. 2021. Mash-based analyses of *Escherichia coli* genomes reveal 14 distinct phylogroups. Commun Biol 4:117. 10.1038/s42003-020-01626-5. 33500552 PMC7838162

[4] Baumler DJ, Peplinski RG, Reed JL, Glasner JD, Perna NT. 2011. The evolution of metabolic networks of *Escherichia coli*. BMC Syst Biol 5:182. 10.1186/1752-0509-5-182. 22044664 PMC3229490

[5] Croxen MA, Finlay BB. 2010. Molecular mechanisms of *Escherichia coli* pathogenicity. Nat Rev Microbiol 8:26–38. 10.1038/nrmicro2265. 19966814

[6] Mainil J. 2013. *Escherichia coli* virulence factors. Vet Immunol Immunopathol 152:2–12. 10.1016/j.vetimm.2012.09.032. 23083938

[7] Gov Y, Bitler A, Dell’Acqua G, Torres JV, Balaban N. 2001. RNAIII inhibiting peptide (RIP), a global inhibitor of *Staphylococcus aureus* pathogenesis: structure and function analysis. Peptides 22:1609–1620. 10.1016/S0196-9781(01)00496-X. 11587789

[8] Dobrindt U, Agerer F, Michaelis K, Janka A, Buchrieser C, Samuelson M, Svanborg C, Gottschalk G, Karch H, Hacker J. 2003. Analysis of genome plasticity in pathogenic and commensal *Escherichia coli* isolates by use of DNA arrays. J Bacteriol 185:1831–1840. 10.1128/jb.185.6.1831-1840.2003. 12618447 PMC150128

[9] Rasko DA, Rosovitz MJ, Myers GSA, Mongodin EF, Fricke WF, Gajer P, Crabtree J, Sebaihia M, Thomson NR, Chaudhuri R, Henderson IR, Sperandio V, Ravel J. 2008. The pangenome structure of *Escherichia coli*: comparative genomic analysis of *E. coli* commensal and pathogenic isolates. J Bacteriol 190:6881–6893. 10.1128/JB.00619-08. 18676672 PMC2566221

[10] Bohlin J, Brynildsrud OB, Sekse C, Snipen L. 2014. An evolutionary analysis of genome expansion and pathogenicity in *Escherichia coli*. BMC Genomics 15:882. 10.1186/1471-2164-15-882. 25297974 PMC4200225

[11] Denamur E, Clermont O, Bonacorsi S, Gordon D. 2021. The population genetics of pathogenic *Escherichia coli*. Nat Rev Microbiol 19:37–54. 10.1038/s41579-020-0416-x. 32826992

[12] Pitout JDD. 2012. Extraintestinal pathogenic *Escherichia coli*: a combination of virulence with antibiotic resistance. Front Microbiol 3. 10.3389/fmicb.2012.00009. PMC3261549 22294983

[13] Brumbaugh AR, Mobley HL. 2012. Preventing urinary tract infection: progress toward an effective *Escherichia coli* vaccine. Expert Rev Vaccines 11:663–676. 10.1586/erv.12.36. 22873125 PMC3498450

[14] Poolman JT, Wacker M. 2016. Extraintestinal pathogenic *Escherichia coli*, a common human pathogen: challenges for vaccine development and progress in the field. J Infect Dis 213:6–13. 10.1093/infdis/jiv429. 26333944 PMC4676548

[15] Rojas-Lopez M, Monterio R, Pizza M, Desvaux M, Rosini R. 2018. Intestinal pathogenic *Escherichia coli*: insights for vaccine development. Front Microbiol 9. 10.3389/fmicb.2018.00440. PMC5869917 29615989

[16] Pappenheimer AM. 1977. Diphtheria toxin. Annu Rev Biochem 46:69–94. 10.1146/annurev.bi.46.070177.000441. 20040

[17] Hadfield TL, McEvoy P, Polotsky Y, Tzinserling VA, Yakovlev AA. 2000. The pathology of diphtheria. J Infect Dis 181:S116–S120. 10.1086/315551. 10657202

[18] Weiss AA, Hewlett EL. 1986. Virulence factors of *Bordetella pertussis*. Annu Rev Microbiol 40:661–686. 10.1146/annurev.mi.40.100186.003305. 2877614

[19] Kilgore PE, Salim AM, Zervos MJ, Schmitt H-J. 2016. Pertussis: microbiology, disease, treatment, and prevention. Clin Microbiol Rev 29:449–486. 10.1128/CMR.00083-15. 27029594 PMC4861987

[20] Plowe CV. 2011. Vaccine design: innovative approaches and novel strategies. Clin Infect Dis 53:318–318. 10.1093/cid/cir415.

[21] Leimbach A, Hacker J, Dobrindt U. 2013. *Escherichia coli* as an all-rounder: the thin line between commensalism and pathogenicity. Curr Top Microbiol Immunol 858:3–32. 10.1007/82_2012_303 23340801

[22] Robins-Browne RM, Holt KE, Ingle DJ, Hocking DM, Yang J, Tauschek M. 2016. Are *Escherichia coli* pathotypes still relevant in the era of whole-genome sequencing? Front Cell Infect Microbiol 6:141. 10.3389/fcimb.2016.00141. 27917373 PMC5114240

[23] Yamamoto S. 2007. Molecular epidemiology of uropathogenic *Escherichia coli*. J Infect Chemother 13:68–73. 10.1007/s10156-007-0506-Y. 17458672

[24] Wiles TJ, Kulesus RR, Mulvey MA. 2008. Origins and virulence mechanisms of uropathogenic *Escherichia coli*. Exp Mol Pathol 85:11–19. 10.1016/j.yexmp.2008.03.007. 18482721 PMC2595135

[25] Sarowska J, Futoma-Koloch B, Jama-Kmiecik A, Frej-Madrzak M, Ksiazczyk M, Bugla-Ploskonska G, Choroszy-Krol I, Ewers C, Janssen T, Wieler LH. 2019. Virulence factors, prevalence, and potential transmission of extraintestinal pathogenic *Escherichia coli* isolated from different sources: recent reports. Gut Pathog 11:10. 10.1186/s13099-019-0290-0. 30828388 PMC6383261

[26] Ewers C, Janssen T, Wieler LH. 2003. Avian pathogenic *Escherichia coli* (APEC). Berl Munch Tierarztl Wochenschr 116:381–395. 14526468

[27] Dho-Moulin M, Fairbrother JM. 1999. Mar-Jun. Avian pathogenic *Escherichia coli* (APEC). Vet Res 30:299–316. 10367360

[28] Rodriguez-Siek KE, Giddings CW, Doetkott C, Johnson TJ, Nolan LK. 2005. Characterizing the APEC pathotype. Vet Res 36:241–256. 10.1051/vetres:2004057. 15720976

[29] Carvalho FA, Barnich N, Sivignon A, Darcha C, Chan CHF, Stanners CP, Darfeuille-Michaud A. 2009. Crohn’s disease adherent-invasive *Escherichia coli* colonize and induce strong gut inflammation in transgenic mice expressing human CEACAM. J Exp Med 206:2179–2189. 10.1084/jem.20090741. 19737864 PMC2757893

[30] Friswell M, Campbell B, Rhodes J. 2010. The role of bacteria in the pathogenesis of inflammatory bowel disease. Gut Liver 4:295–306. 10.5009/gnl.2010.4.3.295. 20981205 PMC2956340

[31] Yang Y, Liao Y, Ma Y, Gong W, Zhu G. 2017. The role of major virulence factors of AIEC involved in inflammatory bowl disease: a mini-review. Appl Microbiol Biotechnol 101:7781–7787. 10.1007/s00253-017-8507-y. 28918511

[32] Clements A, Young JC, Constantinou N, Frankel G. 2012. Infection strategies of enteric pathogenic *Escherichia coli*. Gut Microbes 3:71–87. 10.4161/gmic.19182. 22555463 PMC3370951

[33] Cieza RJ, Hu J, Ross BN, Sbrana E, Torres AG. 2015. The IbeA invasin of adherent-invasive *Escherichia coli* mediates interaction with intestinal epithelia and macrophages. Infect Immun 83:1904–1918. 10.1128/IAI.03003-14. 25712929 PMC4399045

[34] Delannoy S, Beutin L, Fach P. 2013. Discrimination of enterohemorrhagic *Escherichia coli* (EHEC) from non-EHEC strains based on detection of various combinations of type III effector genes. J Clin Microbiol 51:3257–3262. 10.1128/JCM.01471-13. 23884997 PMC3811616

[35] Kaur P, Chakraborti A, Asea A2. 2010. Enteroaggregative *Escherichia coli*: an emerging enteric food borne pathogen. Interdiscip Perspect Infect Dis 2010:254159–254110. 10.1155/2010/254159. 20300577 PMC2837894

[36] Zamboni A, Fabbricotti SH, Fagundes-Neto U, Scaletsky ICA. 2004. Enteroaggregative *Escherichia coli* virulence factors are found to be associated with infantile diarrhea in Brazil. J Clin Microbiol 42:1058–1063. 10.1128/jcm.42.3.1058-1063.2004. 15004053 PMC356835

[37] Beutin L, Martin A. 2012. Outbreak of Shiga toxin-producing *Escherichia coli* (STEC) O104:H4 infection in Germany causes a paradigm shift with regard to human pathogenicity of STEC strains. J Food Prot 75:408–418. 10.4315/0362-028X.JFP-11-452. 22289607

[38] Gaastra W, Svennerholm AM. 1996. Colonization factors of human enterotoxigenic *Escherichia coli* (ETEC). Trends Microbiol 4:444–452. 10.1016/0966-842x(96)10068-8. 8950814

[39] Blanco M, Schumacher S, Tasara T, Zweifel C, Blanco JE, Dahbi G, Blanco J, Stephan R. 2005. Serotypes, intimin variants, and other virulence factors of eae positive *Escherichia coli* strains isolated from healthy cattle in Switzerland. Identification of a new intimin variant gene (eae-η2). BMC Microbiol 5:23–11. 10.1186/1471-2180-5-23. 15882459 PMC1142320

[40] Kenny B, DeVinney R, Stein M, Reinscheid DJ, Frey EA, Finlay BB. 1997. Enteropathogenic *Escherichia coli* (EPEC) transfers its receptor for intimate adherence into mammalian cells. Cell 91:511–520. 10.1016/S0092-8674(00)80437-7. 9390560

[41] Peleg A, Shifrin Y, Ilan O, Nadler-Yona C, Nov S, Koby S, Baruch K, Altuvia S, Elgrably-Weiss M, Abe CM, Knutton S, Saper MA, Rosenshine I. 2005. Identification of an *Escherichia coli* operon required for formation of the O-antigen capsule. J Bacteriol 187:5259–5266. 10.1128/JB.187.15.5259-5266.2005. 16030220 PMC1196049

[42] Herzer PJ, Inouye S, Inouye M, Whittam TS. 1990. Phylogenetic distribution of branched RNA-linked multicopy single-stranded DNA among natural isolates of *Escherichia coli*. J Bacteriol 172:6175–6181. 10.1128/jb.172.11.6175-6181.1990. 1699928 PMC526797

[43] Clermont O, Bonacorsi S, Bingen E. 2000. Rapid and simple determination of the *Escherichia coli* phylogenetic group. Appl Environ Microbiol 66:4555–4558. 10.1128/aem.66.10.4555-4558.2000. 11010916 PMC92342

[44] Tenaillon O, Skurnik D, Picard B, Denamur E. 2010. The population genetics of commensal *Escherichia coli*. Nat Rev Microbiol 8:207–217. 10.1038/nrmicro2298. 20157339

[45] Honsa ES, Maresso AW, Sarah E, Anthony H, Maresso W. 2011. Mechanisms of iron import in anthrax. Biometals 24:533–545. 10.1007/s10534-011-9413-x. 21258843

[46] Skaar EP. 2010. The battle for iron between bacterial pathogens and their vertebrate hosts. PLoS Pathog 6:e1000949. 10.1371/journal.ppat.1000949. 20711357 PMC2920840

[47] Contreras H, Chim N, Credali A, Goulding CW. 2014. Heme uptake in bacterial pathogens. Curr Opin Chem Biol 19:34–41. 10.1016/j.cbpa.2013.12.014. 24780277 PMC4007353

[48] Jaureguy F, Landraud L, Passet V, Diancourt L, Frapy E, Guigon G, Carbonnelle E, Lortholary O, Clermont O, Denamur E, Picard B, Nassif X, Brisse S. 2008. Phylogenetic and genomic diversity of human bacteremic *Escherichia coli* strains. BMC Genomics 9:560. 10.1186/1471-2164-9-560. 19036134 PMC2639426

[49] Nowrouzian FL, Wold AE, Adlerberth I. 2005. *Escherichia coli* strains belonging to phylogenetic group B2 have superior capacity to persist in the intestinal microflora of infants. J Infect Dis 191:1078–1083. 10.1086/427996. 15747243

[50] Le Gall T, Clermont O, Gouriou S, Picard B, Nassif X, Denamur E, Tenaillon O. 2007. Extraintestinal virulence is a coincidental by-product of commensalism in B2 phylogenetic group *Escherichia coli* strains. Mol Biol Evol 24:2373–2384. 10.1093/molbev/msm172. 17709333

[51] Papouskova A, Masarikova M, Valcek A, Senk D, Cejkova D, Jahodarova E, Cizek A. 2020. Genomic analysis of *Escherichia coli* strains isolated from diseased chicken in the Czech Republic. BMC Vet Res 16:189. 10.1186/s12917-020-02407-2. 32522212 PMC7286222

[52] Cordoni G, Woodward MJ, Wu H, Alanazi M, Wallis T, La Ragione RM. 2016. Comparative genomics of European avian pathogenic *Escherichia coli* (APEC). BMC Genomics 17:960. 10.1186/s12864-016-3289-7. 27875980 PMC5120500

[53] Borzi MM, Cardozo MV, de Oliveira ES, de Pollo AS, Guastalli EAL, dos Santos LF, de Ávila FA. 2018. Characterization of avian pathogenic *Escherichia coli* isolated from free-range helmeted guineafowl. Brazilian J Microbiol 49:107–112. 10.1016/j.bjm.2018.04.011. PMC6328720 30170963

[54] Vangchhia B, Abraham S, Bell JM, Collignon P, Gibson JS, Ingram PR, Johnson JR, Kennedy K, Trott DJ, Turnidge JD, Gordon DM. 2016. Phylogenetic diversity, antimicrobial susceptibility, and virulence characteristics of phylogroup F *Escherichia coli* in Australia. Microbiology (Reading) 162:1904–1912. 10.1099/mic.0.000367. 27666313

[55] Clermont O, Dixit OVA, Vangchhia B, Condamine B, Dion S, Bridier‐Nahmias A, Denamur E, Gordon D. 2019. Characterization and rapid identification of phylogroup G in *Escherichia coli*, a lineage with high virulence and antibiotic resistance potential. Environ Microbiol 21:3107–3117. 10.1111/1462-2920.14713. 31188527

[56] Sayers S, Li L, Ong E, Deng S, Fu G, Lin Y, Yang B, Zhang S, Fa Z, Zhao B, Xiang Z, Li Y, Zhao X-M, Olszewski MA, Chen L, He Y. 2019. Victors: a web-based knowledge base of virulence factors in human and animal pathogens. Nucleic Acids Res 47:D693–D700. 10.1093/nar/gky999. 30365026 PMC6324020

[57] Davis JJ, Wattam AR, Aziz RK, Brettin T, Butler R, Butler RM, Chlenski P, Conrad N, Dickerman A, Dietrich EM, Gabbard JL, Gerdes S, Guard A, Kenyon RW, Machi D, Mao C, Murphy-Olson D, Nguyen M, Nordberg EK, Olsen GJ, Olson RD, Overbeek JC, Overbeek R, Parrello B, Pusch GD, Shukla M, Thomas C, VanOeffelen M, Vonstein V, Warren AS, Xia F, Xie D, Yoo H, Stevens R. 2020. The PATRIC Bioinformatics Resource Center: expanding data and analysis capabilities. Nucleic Acids Res 47:D606–D612. 10.1093/nar/gkz943. PMC7145515 31667520

[58] Chen L, Zheng D, Liu B, Yang J, Jin Q. 2016. VFDB 2016: hierarchical and refined dataset for big data analysis—10 years on. Nucleic Acids Res 44:D694–D697. 10.1093/nar/gkv1239. 26578559 PMC4702877

[59] Roubaud Baudron C, Panhard X, Clermont O, Mentre F, Fantin B, Denamur E, Lefort A. 2014. *Escherichia coli* bacteremia in adults: age-related differences in clinical and bacteriological characteristics, and outcome. Epidemiol Infect 142:2672–2683. 10.1017/S0950268814000211. 24559489 PMC9151316

[60] Gordon DM, Clermont O, Tolley H, Denamur E. 2008. Assigning *Escherichia coli* strains to phylogenetic groups: multilocus sequence typing versus the PCR triplex method. Environ Microbiol 10:2484–2496. 10.1111/j.1462-2920.2008.01669.x. 18518895

[61] Clermont O, Christenson JK, Denamur E, Gordon DM. 2013. The Clermont *Escherichia coli* phylo-typing method revisited: improvement of specificity and detection of new phylogroups. Environ Microbiol Rep 5:58–65. 10.1111/1758-2229.12019. 23757131

[62] Miajlovic H, Smith SG. 2014. Bacterial self-defence: how *Escherichia coli* evades serum killing. FEMS Microbiol Lett 354:1–9. 10.1111/1574-6968.12419. 24617921

[63] Sperandeo P, Martorana AM, Polissi A. 2017. Lipopolysaccharide biogenesis and transport at the outer membrane of Gram-negative bacteria. Biochim Biophys Acta Mol Cell Biol Lipids 1862:1451–1460. 10.1016/j.bbalip.2016.10.006. 27760389

[64] Whitfield C. 2006. Biosynthesis and assembly of capsular polysaccharides in *Escherichia coli*. Annu Rev Biochem 75:39–68. 10.1146/annurev.biochem.75.103004.142545. 16756484

[65] Whitfield GB, Marmont LS, Howell PL. 2015. Enzymatic modifications of exopolysaccharides enhance bacterial persistence. Front Microbiol 6:471. 10.3389/fmicb.2015.00471. 26029200 PMC4432689

[66] Sachdeva S, Palur RV, Sudhakar KU, Rathinavelan T. 2017. *E. coli* group 1 capsular polysaccharide exportation nanomachinary as a plausible antivirulence target in the perspective of emerging antimicrobial resistance. Front Microbiol 8:70. 10.3389/fmicb.2017.00070. 28217109 PMC5290995

[67] Franzin FM, Sircili MP. 2015. Locus of enterocyte effacement: a pathogenicity island involved in the virulence of enteropathogenic and enterohemorragic *Escherichia coli* subjected to a complex network of gene regulation. Biomed Res Int 2015:534738–534710. 10.1155/2015/534738. 25710006 PMC4332760

[68] Kotlowski R, Bernstein CN, Sepehri S, Krause DO. 2007. High prevalence of *Escherichia coli* belonging to the B2+D phylogenetic group in inflammatory bowel disease. Gut 56:669–675. 10.1136/gut.2006.099796. 17028128 PMC1942160

[69] Buckles EL, Wang X, Lane MC, Lockatell CV, Johnson DE, Rasko DA, Mobley HLT, Donnenberg MS. 2009. Role of the K2 capsule in *Escherichia coli* urinary tract infection and serum resistance. J Infect Dis 199:1689–1697. 10.1086/598524. 19432551 PMC3872369

[70] Goller CC, Seed PC. 2010. Revisiting the *Escherichia coli* polysaccharide capsule as a virulence factor during urinary tract infection: contribution to intracellular biofilm development. Virulence 1:333–337. 10.4161/viru.1.4.12388. 21178466

[71] Hafez M, Hayes K, Goldrick M, Warhurst G, Grencis R, Roberts IS. 2009. The K5 capsule of *Escherichia coli* strain Nissle 1917 is important in mediating interactions with intestinal epithelial cells and chemokine induction. Infect Immun 77:2995–3003. 10.1128/IAI.00040-09. 19380467 PMC2708560

[72] Kim KJ, Elliott SJ, Di Cello F, Stins MF, Kim KS. 2003. The K1 capsule modulates trafficking of *Escherichia coli*-containing vacuoles and enhances intracellular bacterial survival in human brain microvascular endothelial cells. Cell Microbiol 5:245–252. 10.1046/j.1462-5822.2003.t01-1-00271.x. 12675682

[73] Bertozzi Silva J, Storms Z, Sauvageau D. 2016. Host receptors for bacteriophage adsorption. FEMS Microbiol Lett 363:fnw002. 10.1093/femsle/fnw002. 26755501

[74] Mangalea MR, Duerkop BA. 2020. Fitness trade-offs resulting from bacteriophage resistance potentiate synergistic antibacterial strategies. Infect Immun 88:e00926-19. 10.1128/IAI.00926-19. 32094257 PMC7309606

[75] Antão E-M, Wieler LH, Ewers C. 2009. Adhesive threads of extraintestinal pathogenic *Escherichia coli*. Gut Pathog 1:22. 10.1186/1757-4749-1-22. 20003270 PMC2797515

[76] Klemm P. 1985. Fimbrial adhesins of *Escherichia coli*. Rev Infect Dis 7:321–340. 10.1093/clinids/7.3.321. 2862689

[77] Brinton CC. 1965. The structure, function, synthesis, and genetic control of bacterial pili and a molecular model for DNA and RNA transport in Gram-negative bacteria. Trans N Y Acad Sci 27:1003–1054. 10.1111/j.2164-0947.1965.tb02342.x. 5318403

[78] Duguid JP, Smith IW, Dempster G, Edmunds PN. 1955. Non-flagellar filamentous appendages (“fimbriæ”) and hæmagglutinating activity in bacterium coli. J Pathol Bacteriol 70:335–348. 10.1002/path.1700700210. 13295908

[79] Johnson JR. 1991. Virulence factors in *Escherichia coli* urinary tract infection. Clin Microbiol Rev 4:80–128. 10.1128/cmr.4.1.80. 1672263 PMC358180

[80] Bergsten G, Wullt B, Svanborg C. 2005. *Escherichia coli*, fimbriae, bacterial persistence, and host response induction in the human urinary tract. Int J Med Microbiol 295:487–502. 10.1016/j.ijmm.2005.07.008. 16238023

[81] Dhakal BK, Kulesus RR, Mulvey MA. 2008. Mechanisms and consequences of bladder cell invasion by uropathogenic *Escherichia coli*. Eur J Clin Invest 38:2–11. 10.1111/j.1365-2362.2008.01986.x. 18616559

[82] Pouttu R, Puustinen T, Virkola R, Hacker J, Klemm P, Korhonen TK. 1999. Amino acid residue Ala-62 in the FimH fimbrial adhesin is critical for the adhesiveness of meningitis-associated *Escherichia coli* to collagens. Mol Microbiol 31:1747–1757. 10.1046/j.1365-2958.1999.01311.x. 10209747

[83] Sokurenko EV, Courtney HS, Ohman DE, Klemm P, Hasty DL. 1994. FimH family of type 1 fimbrial adhesins: functional heterogeneity due to minor sequence variations among *fimH* genes. J Bacteriol 176:748–755. 10.1128/jb.176.3.748-755.1994. 7905476 PMC205112

[84] Kukkonen M, Raunio T, Virkola R, Lähteenmäki K, Mäkelä PH, Klemm P, Clegg S, Korhonen TK. 1993. Basement membrane carbohydrate as a target for bacterial adhesion: binding of type I fimbriae of *Salmonella enterica* and *Escherichia coli* to laminin. Mol Microbiol 7:229–237. 10.1111/j.1365-2958.1993.tb01114.x. 8095317

[85] Sharon N. 1987. Bacterial lectins, cell-cell recognition and infectious disease. FEBS Lett 217:145–157. 10.1016/0014-5793(87)80654-3. 2885220

[86] Barnich N, Carvalho FA, Glasser A, Darcha C, Jantscheff P, Allez M, Peeters H, Bommelaer G, Desreumaux P, Colombel J-F, Darfeuille-Michaud A. 2007. CEACAM6 acts as a receptor for mucosa colonization in Crohn disease. J Clin Invest 117:1566–1574. 10.1172/JCI30504. 17525800 PMC1868786

[87] Connell I, Agace W, Klemm P, Schembri M, Mărild S, Svanborg C. 1996. Type 1 fimbrial expression enhances *Escherichia coli* virulence for the urinary tract. Proc Natl Acad Sci U S A 93:9827–9832. 10.1073/pnas.93.18.9827. 8790416 PMC38514

[88] Wright KJ, Seed PC, Hultgren SJ. 2007. Development of intracellular bacterial communities of uropathogenic *Escherichia coli* depends on type 1 pili. Cell Microbiol 9:2230–2241. 10.1111/j.1462-5822.2007.00952.x. 17490405

[89] Bergsten G, Wullt B, Schembri MA, Leijonhufvud I, Svanborg C. 2007. Do type 1 fimbriae promote inflammation in the human urinary tract? Cell Microbiol 9:1766–1781. 10.1111/j.1462-5822.2007.00912.x. 17359236

[90] Moreira CG, Carneiro SM, Nataro JP, Trabulsi LR, Elias WP. 2003. Role of type I fimbriae in the aggregative adhesion pattern of enteroaggregative *Escherichia coli*. FEMS Microbiol Lett 226:79–85. 10.1016/S0378-1097(03)00561-5. 13129611

[91] Chaudhuri RR, Sebaihia M, Hobman JL, Webber MA, Leyton DL, Goldberg MD, Cunningham AF, Scott-Tucker A, Ferguson PR, Thomas CM, Frankel G, Tang CM, Dudley EG, Roberts IS, Rasko DA, Pallen MJ, Parkhill J, Nataro JP, Thomson NR, Henderson IR. 2010. Complete genome sequence and comparative metabolic profiling of the prototypical enteroaggregative *Escherichia coli* strain 042. PLoS One 5:e8801. 10.1371/journal.pone.0008801. 20098708 PMC2808357

[92] Servin AL. 2005. Pathogenesis of Afa/Dr diffusely adhering *Escherichia coli*. Clin Microbiol Rev 18:264–292. 10.1128/CMR.18.2.264-292.2005. 15831825 PMC1082796

[93] Beatson SA, Ben Zakour NL, Totsika M, Forde BM, Watts RE, Mabbett AN, Szubert JM, Sarkar S, Phan M-D, Peters KM, Petty NK, Alikhan N-F, Sullivan MJ, Gawthorne JA, Stanton-Cook M, Nhu NTK, Chong TM, Yin W-F, Chan K-G, Hancock V, Ussery DW, Ulett GC, Schembri MA. 2015. Molecular analysis of asymptomatic bacteriuria *Escherichia coli* strain VR50 reveals adaptation to the urinary tract by gene acquisition. Infect Immun 83:1749–1764. 10.1128/IAI.02810-14. 25667270 PMC4399054

[94] Le Bouguenec C, Lalioui L, Du Merle L, Jouve M, Courcoux P, Bouzari S, Selvarangan R, Nowicki BJ, Germani Y, Andremont A, Gounon P, Garcia M-I. 2001. Characterization of AfaE adhesins produced by extraintestinal and intestinal human *Escherichia coli* isolates: PCR assays for detection of Afa adhesins that do or do not recognize Dr blood group antigens. J Clin Microbiol 39:1738–1745. 10.1128/JCM.39.5.1738-1745.2001. 11325983 PMC88018

[95] Li Y-F, Poole S, Nishio K, Jang K, Rasulova F, McVeigh A, Savarino SJ, Xia D, Bullitt E. 2009. Structure of CFA/I fimbriae from enterotoxigenic *Escherichia coli*. Proc Natl Acad Sci U S A 106:10793–10798. 10.1073/pnas.0812843106. 19515814 PMC2705562

[96] Samadder P, Xicohtencatl-Cortes J, Saldaña Z, Jordan D, Tarr PI, Kaper JB, Girón JA. 2009. The *Escherichia coli* ycbQRST operon encodes fimbriae with laminin-binding and epithelial cell adherence properties in Shiga-toxigenic *E. coli* O157:H7. Environ Microbiol 11:1815–1826. 10.1111/j.1462-2920.2009.01906.x. 19508558 PMC2888687

[97] Riegman N, Kusters R, Van Veggel H, Bergmans H, Van Bergen En Henegouwen P, Hacker J, Van Die I. 1990. F1C fimbriae of a uropathogenic *Escherichia coli* strain: genetic and functional organization of the *foc* gene cluster and identification of minor subunits. J Bacteriol 172:1114–1120. 10.1128/jb.172.2.1114-1120.1990. 1967600 PMC208544

[98] Bäckhed F, Alsén B, Roche N, Ångström J, von Euler A, Breimer ME, Westerlund-Wikström B, Teneberg S, Richter-Dahlfors A. 2002. Identification of target tissue glycosphingolipid receptors for uropathogenic, F1C-fimbriated *Escherichia coli* and its role in mucosal inflammation. J Biol Chem 277:18198–18205. 10.1074/jbc.M111640200. 11877427

[99] Klemm P, Christiansen G, Kreft B, Marre R, Bergmans H. 1994. Reciprocal exchange of minor components of type 1 and F1C fimbriae results in hybrid organelles with changed receptor specificities. J Bacteriol 176:2227–2234. 10.1128/jb.176.8.2227-2234.1994. 7908902 PMC205343

[100] Khan AS, Kniep B, Oelschlaeger TA, Van Die I, Korhonen T, Hacker J. 2000. Receptor structure for F1C fimbriae of uropathogenic *Escherichia coli*. Infect Immun 68:3541–3547. 10.1128/iai.68.6.3541-3547.2000. 10816509 PMC97640

[101] Mulvey MA. 2002. Adhesion and entry of uropathogenic *Escherichia coli*. Cell Microbiol 4:257–271. 10.1046/j.1462-5822.2002.00193.x. 12027955

[102] Lasaro MA, Salinger N, Zhang J, Wang Y, Zhong Z, Goulian M, Zhu J. 2009. F1C fimbriae play an important role in biofilm formation and intestinal colonization by the *Escherichia coli* commensal strain Nissle 1917. Appl Environ Microbiol 75:246–251. 10.1128/AEM.01144-08. 18997018 PMC2612203

[103] Dobrindt U, Blum-Oehler G, Nagy G, Schneider G, Johann A, Gottschalk G, Hacker J. 2002. Genetic structure and distribution of four pathogenicity islands (PAI I536 to PAI IV536) of uropathogenic *Escherichia coli* strain 536. Infect Immun 70:6365–6372. 10.1128/iai.70.11.6365-6372.2002. 12379716 PMC130402

[104] Ott M, Hoschützky H, Jann K, Van Die I, Hacker J. 1988. Gene clusters for S fimbrial adhesin (*sfa*) and F1C fimbriae (*foc*) of *Escherichia coli*: comparative aspects of structure and function. J Bacteriol 170:3983–3990. 10.1128/jb.170.9.3983-3990.1988. 2900831 PMC211399

[105] Adlerberth I, Hanson ÅL, Svanborg C, Svennerholm A, Nordgren S, Wold AE. 1995. Adhesins of *Escherichia coli*-associated with extra-intestinal pathogenicity confer binding to colonic epithelial cells. Microb Pathog 18:373–385. 10.1006/mpat.1995.0034. 8551941

[106] Hull RA, Gill RE, Hsu P, Minshew BH, Falkow S. 1981. Construction and expression of recombinant plasmids encoding type 1 or d-mannose-resistant pili from a urinary tract infection *Escherichia coli* isolate. Infect Immun 33:933–938. 10.1128/IAI.33.3.933-938.1981. 6116675 PMC350799

[107] Rasko DA, Phillips JA, Li X, Mobley HLT. 2001. Identification of DNA sequences from a second pathogenicity island of uropathogenic *Escherichia coli* CFT073: probes specific for uropathogenic populations. J Infect Dis 184:1041–1049. 10.1086/323602. 11574920

[108] Jadhav S, Hussain A, Devi S, Kumar A, Parveen S, Gandham N, Wieler LH, Ewers C, Ahmed N. 2011. Virulence characteristics and genetic affinities of multiple drug-resistant uropathogenic *Escherichia coli* from a semi-urban locality in India. PLoS One 6:e18063. 10.1371/journal.pone.0018063. 21464963 PMC3064663

[109] Lindberg S, Xia Y, Sondén B, Göransson M, Hacker J, Uhlin BE. 2008. Regulatory interactions among adhesin gene systems of uropathogenic *Escherichia coli*. Infect Immun 76:771–780. 10.1128/IAI.01010-07. 18039830 PMC2223471

[110] Simms AN, Mobley HLT. 2008. PapX, a P fimbrial operon-encoded inhibitor of motility in uropathogenic *Escherichia coli*. Infect Immun 76:4833–4841. 10.1128/IAI.00630-08. 18710869 PMC2573324

[111] Kawamura T, Le LUK, Zhou H, Dahlquist FW. 2007. Solution structure of *Escherichia coli* PapI, a key regulator of the Pap pilus phase variation. J Mol Biol 365:1130–1142. 10.1016/j.jmb.2006.10.066. 17109885 PMC2594013

[112] Lund B, Lindberg F, Marklund BI, Normark S. 1987. The PapG protein is the α-d-galactopyranosyl-(1–4)-β-d-galactopyranose-binding adhesin of uropathogenic *Escherichia coli*. Proc Natl Acad Sci U S A 84:5898–5902. 10.1073/pnas.84.16.5898. 2886993 PMC298970

[113] Wilks A, Heinzl G. 2014. Heme oxygenation and the widening paradigm of heme degradation. Arch Biochem Biophys 544:87–95. 10.1016/j.abb.2013.10.013. 24161941 PMC6476305

[114] Weinberg ED. 2009. Iron availability and infection. Biochim Biophys Acta 1790:600–605. 10.1016/j.bbagen.2008.07.002. 18675317

[115] Skaar EP, Raffatellu M. 2015. Metals in infectious diseases and nutritional immunity. Metallomics 7:926–928. 10.1039/c5mt90021b. 26017093

[116] Subashchandrabose S, Mobley HLT. 2015. Back to the metal age: battle for metals at the host-pathogen interface during urinary tract infection. Metallomics 7:935–942. 10.1039/c4mt00329b. 25677827 PMC4634365

[117] Galardini M, Clermont O, Baron A, Busby B, Dion S, Schubert S, Beltrao P, Denamur E. 2020. Major role of iron uptake systems in the intrinsic extra-intestinal virulence of the genus *Escherichia* revealed by a genome-wide association study. PLoS Genet 16:e1009065. 10.1371/journal.pgen.1009065. 33112851 PMC7592755

[118] Perry RD, Fetherston JD. 2011. Yersiniabactin iron uptake: mechanisms and role in *Yersinia pestis* pathogenesis. Microbes Infect 13:808–817. 10.1016/j.micinf.2011.04.008. 21609780 PMC3148425

[119] Torres AG, Payne SM. 1997. Haem iron-transport system in enterohaemorrhagic *Escherichia coli* O157:H7. Mol Microbiol 23:825–833. 10.1046/j.1365-2958.1997.2641628.x. 9157252

[120] Crossman LC, Chaudhuri RR, Beatson SA, Wells TJ, Desvaux M, Cunningham AF, Petty NK, Mahon V, Brinkley C, Hobman JL, Savarino SJ, Turner SM, Pallen MJ, Penn CW, Parkhill J, Turner AK, Johnson TJ, Thomson NR, Smith SGJ, Henderson IR. 2010. A commensal gone bad: complete genome sequence of the prototypical enterotoxigenic *Escherichia coli* strain H10407. J Bacteriol 192:5822–5831. 10.1128/JB.00710-10. 20802035 PMC2953697

[121] Reigstad CS, Hultgren SJ, Gordon JI. 2007. Functional genomic studies of uropathogenic *Escherichia coli* and host urothelial cells when intracellular bacterial communities are assembled. J Biol Chem 282:21259–21267. 10.1074/jbc.M611502200. 17504765

[122] Torres AG, Redford P, Welch RA, Payne SM. 2001. TonB-dependent systems of uropathogenic *Escherichia coli*: aerobactin and heme transport and TonB are required for virulence in the mouse. Infect Immun 69:6179–6185. 10.1128/IAI.69.10.6179-6185.2001. 11553558 PMC98749

[123] Spurbeck RR, Dinh PC, Walk ST, Stapleton AE, Hooton TM, Nolan LK, Kim KS, Johnson JR, Mobley HLT. 2012. *Escherichia coli* isolates that carry *vat*, *fyuA*, *chuA*, and *yfcV* efficiently colonize the urinary tract. Infect Immun 80:4115–4122. 10.1128/IAI.00752-12. 22966046 PMC3497434

[124] Russo TA, McFadden CD, Carlino-MacDonald UB, Beanan JM, Barnard TJ, Johnson JR. 2002. IroN functions as a siderophore receptor and is a urovirulence factor in an extraintestinal pathogenic isolate of *Escherichia coli*. Infect Immun 70:7156–7160. 10.1128/iai.70.12.7156-7160.2002. 12438401 PMC133021

[125] Desvaux M, Dalmasso G, Beyrouthy R, Barnich N, Delmas J, Bonnet R. 2020. Pathogenicity factors of genomic islands in intestinal and extraintestinal *Escherichia coli*. Front Microbiol 11:2065. 10.3389/fmicb.2020.02065. 33101219 PMC7545054

[126] Sabri M, Léveillé S, Dozois CM. 2006. A SitABCD homologue from an avian pathogenic *Escherichia coli* strain mediates transport of iron and manganese and resistance to hydrogen peroxide. Microbiology (Reading) 152:745–758. 10.1099/mic.0.28682-0. 16514154

[127] Peigne C, Bidet P, Mahjoub-Messai F, Plainvert C, Barbe V, Médigue C, Frapy E, Nassif X, Denamur E, Bingen E, Bonacorsi S. 2009. The plasmid of *Escherichia coli* strain S88 (O45:K1:H7) that causes neonatal meningitis is closely related to avian pathogenic *E. coli* plasmids and is associated with high-level bacteremia in a neonatal rat meningitis model. Infect Immun 77:2272–2284. 10.1128/IAI.01333-08. 19307211 PMC2687354

[128] Li Y, Dai J, Zhuge X, Wang H, Hu L, Ren J, Chen L, Li D, Tang F. 2016. Iron-regulated gene *ireA* in avian pathogenic *Escherichia coli* participates in adhesion and stress-resistance. BMC Vet Res 12:167. 10.1186/s12917-016-0800-y. 27531140 PMC4988017

[129] Feldmann F, Sorsa LJ, Hildinger K, Schubert S. 2007. The salmochelin siderophore receptor IroN contributes to invasion of urothelial cells by extraintestinal pathogenic *Escherichia coli in vitro*. Infect Immun 75:3183–3187. 10.1128/IAI.00656-06. 17353289 PMC1932905

[130] Bauer RJ, Zhang L, Foxman B, Siitonen A, Jantunen ME, Saxen H, Marrs CF. 2002. Molecular epidemiology of 3 putative virulence genes for *Escherichia coli* urinary tract infection: *usp*, *iha*, and *iroN E. coli*. J Infect Dis 185:1521–1524. 10.1086/340206. 11992291

[131] Hantke K, Nicholson G, Rabsch W, Winkelmann G. 2003. Salmochelins, siderophores of *Salmonella enterica* and uropathogenic *Escherichia coli* strains, are recognized by the outer membrane receptor IroN. Proc Natl Acad Sci U S A 100:3677–3682. 10.1073/pnas.0737682100. 12655053 PMC152981

[132] Russo TA, Carlino UB, Mong A, Jodush ST. 1999. Identification of genes in an extraintestinal isolate of *Escherichia coli* with increased expression after exposure to human urine. Infect Immun 67:5306–5314. 10.1128/IAI.67.10.5306-5314.1999. 10496910 PMC96885

[133] Caza M, Lépine F, Milot S, Dozois CM. 2008. Specific roles of the *iroBCDEN* genes in virulence of an avian pathogenic *Escherichia coli* O78 strain and in production of salmochelins. Infect Immun 76:3539–3549. 10.1128/IAI.00455-08. 18541653 PMC2493193

[134] Hagan EC, Lloyd AL, Rasko DA, Faerber GJ, Mobley HLT. 2010. *Escherichia coli* global gene expression in urine from women with urinary tract infection. PLoS Pathog 6:e1001187. 10.1371/journal.ppat.1001187. 21085611 PMC2978726

[135] Garcia EC, Brumbaugh AR, Mobley HLT. 2011. Redundancy and specificity of *Escherichia coli* iron acquisition systems during urinary tract infection. Infect Immun 79:1225–1235. 10.1128/IAI.01222-10. 21220482 PMC3067483

[136] Snyder JA, Haugen BJ, Buckles EL, Lockatell CV, Johnson DE, Donnenberg MS, Welch RA, Mobley HLT. 2004. Transcriptome of uropathogenic *Escherichia coli* during urinary tract infection. Infect Immun 72:6373–6381. 10.1128/IAI.72.11.6373-6381.2004. 15501767 PMC523057

[137] Hagan EC, Mobley HLT. 2009. Haem acquisition is facilitated by a novel receptor Hma and required by uropathogenic *Escherichia coli* for kidney infection. Mol Microbiol 71:79–91. 10.1111/j.1365-2958.2008.06509.x. 19019144 PMC2736550

[138] Mike LA, Smith SN, Sumner CA, Eaton KA, Mobley HLT. 2016. Siderophore vaccine conjugates protect against uropathogenic *Escherichia coli* urinary tract infection. Proc Natl Acad Sci U S A 113:13468–13473. 10.1073/pnas.1606324113. 27821778 PMC5127358

[139] Gorden PJ, Kleinhenz MD, Ydstie JA, Brick TA, Slinden LM, Peterson MP, Straub DE, Burkhardt DT. 2018. Efficacy of vaccination with a *Klebsiella pneumoniae* siderophore receptor protein vaccine for reduction of *Klebsiella mastitis* in lactating cattle. J Dairy Sci 101:10398–10408. 10.3168/jds.2017-14267. 30197148

[140] Fox JT, Thomson DU, Drouillard JS, Thornton AB, Burkhardt DT, Emery DA, Nagaraja TG. 2009. Efficacy of *Escherichia coli* O157:H7 siderophore receptor/porin protein-based vaccine in feedlot cattle naturally shedding *E. coli* O157. Foodborne Pathog Dis 6:893–899. 10.1089/fpd.2009.0336. 19737065

[141] Thornton AB, Thomson DU, Loneragan GH, Fox JT, Burkhardt DT, Emery DA, Nagaraja TG. 2009. Effects of a siderophore receptor and porin protein-based vaccination on fecal shedding of *Escherichia coli* O157:H7 in experimentally inoculated cattle. J Food Prot 72:866–869. 10.4315/0362-028X-72.4.866. 19435240

[142] Sassone-Corsi M, Chairatana P, Zheng T, Perez-Lopez A, Edwards RA, George MD, Nolan EM, Raffatellu M. 2016. Siderophore-based immunization strategy to inhibit growth of enteric pathogens. Proc Natl Acad Sci U S A 113:13462–13467. 10.1073/pnas.1606290113. 27821741 PMC5127304

[143] Saha M, Sarkar S, Sarkar B, Sharma BK, Bhattacharjee S, Tribedi P. 2016. Microbial siderophores and their potential applications: a review. Environ Sci Pollut Res Int 23:3984–3999. 10.1007/s11356-015-4294-0. 25758420

[144] Habibi M, Asadi Karam MR, Bouzari S. 2017. Evaluation of prevalence, immunogenicity and efficacy of FyuA iron receptor in uropathogenic *Escherichia coli* isolates as a vaccine target against urinary tract infection. Microb Pathog 110:477–483. 10.1016/j.micpath.2017.07.037. 28754265

[145] Forsyth VS, Himpsl SD, Smith SN, Sarkissian CA, Mike LA, Stocki JA, Sintsova A, Alteri CJ, Mobley HLT. 2020. Optimization of an experimental vaccine to prevent *Escherichia coli* urinary tract infection. mBio 11:e00555-20. 10.1128/mBio.00555-20. 32345645 PMC7188996

[146] Jinadasa RN, Bloom SE, Weiss RS, Duhamel GE. 2011. Cytolethal distending toxin: a conserved bacterial genotoxin that blocks cell cycle progression, leading to apoptosis of a broad range of mammalian cell lineages. Microbiology (Reading) 157:1851–1875. 10.1099/mic.0.049536-0. 21565933 PMC3167888

[147] Hofman P, Le Negrate G, Mograbi B, Hofman V, Brest P, Alliana-Schmid A, Flatau G, Boquet P, Rossi B. 2000. *Escherichia coli* cytotoxic necrotizing factor-1 (CNF-1) increases the adherence to epithelia and the oxidative burst of human polymorphonuclear leukocytes but decreases bacteria phagocytosis. J Leukoc Biol 68:522–528. 11037974

[148] Ludwig A, von Rhein C, Bauer S, Hüttinger C, Goebel W. 2004. Molecular analysis of cytolysin A (ClyA) in pathogenic *Escherichia coli* strains. J Bacteriol 186:5311–5320. 10.1128/JB.186.16.5311-5320.2004. 15292132 PMC490944

[149] Wallace AJ, Stillman TJ, Atkins A, Jamieson SJ, Bullough PA, Green J, Artymiuk PJ. 2000. *Escherichia coli* hemolysin E (HlyE, ClyA, SheA). Cell 100:265–276. 10.1016/S0092-8674(00)81564-0. 10660049

[150] Kerenyi M, Allison HE, Batai I, Sonnevend A, Emody L, Plaveczky N, Pal T. 2005. Occurrence of *hlyA* and *sheA* genes in extraintestinal *Escherichia coli* strains. J Clin Microbiol 43:2965–2968. 10.1128/JCM.43.6.2965-2968.2005. 15956433 PMC1151894

[151] Wiles TJ, Mulvey MA. 2013. The RTX pore-forming toxin α-hemolysin of uropathogenic *Escherichia coli*: progress and perspectives. Future Microbiol 8:73–84. 10.2217/fmb.12.131. 23252494 PMC3570152

[152] Schroeder GN, Hilbi H. 2008. Molecular pathogenesis of *Shigella* spp.: controlling host cell signaling, invasion, and death by type III secretion. Clin Microbiol Rev 21:134–156. 10.1128/CMR.00032-07. 18202440 PMC2223840

[153] Estrada-Garcia T, Navarro-Garcia F. 2012. Enteroaggregative *Escherichia coli* pathotype: a genetically heterogeneous emerging foodborne enteropathogen. FEMS Immunol Med Microbiol 66:281–298. 10.1111/j.1574-695X.2012.01008.x. 22775224

[154] Vila J, Vargas M, Henderson IR, Gascón J, Nataro JP. 2000. Enteroaggregative *Escherichia coli* virulence factors in traveler’s diarrhea strains. J Infect Dis 182:1780–1783. 10.1086/317617. 11069254

[155] Mirzarazi M, Rezatofighi SE, Pourmahdi M, Mohajeri MR. 2015. Occurrence of genes encoding enterotoxins in uropathogenic *Escherichia coli* isolates. Braz J Microbiol 46:155–159. 10.1590/S1517-838246120130860. 26221102 PMC4512059

[156] Östblom A, Adlerberth I, Wold AE, Nowrouzian FL. 2011. Pathogenicity island markers, virulence determinants *malX* and *usp*, and the capacity of *Escherichia coli* to persist in infants’ commensal microbiotas. Appl Environ Microbiol 77:2303–2308. 10.1128/AEM.02405-10. 21317254 PMC3067437

[157] Hanley P, Lalonde G, Ji G. 1991. Alpha-hemolysin contributes to the pathogenicity of piliated digalactoside-binding *Escherichia coli* in the kidney: efficacy of an alpha-hemolysin vaccine in preventing renal injury in the BALB/c mouse model of pyelonephritis. Infect Immun 59:1153–1161. 10.1128/IAI.59.3.1153-1161.1991. 1671776 PMC258381

[158] Smith MA, Weingarten RA, Russo LM, Ventura CL, O’Brien AD. 2015. Antibodies against hemolysin and cytotoxic necrotizing factor type 1 (CNF1) reduce bladder inflammation in a mouse model of urinary tract infection with toxigenic uropathogenic *Escherichia coli*. Infect Immun 83:1661–1673. 10.1128/IAI.02848-14. 25667267 PMC4363410

[159] Henderson IR, Navarro-Garcia F, Desvaux M, Fernandez RC, Ala’Aldeen D. 2004. Type V protein secretion pathway: the autotransporter story. Microbiol Mol Biol Rev 68:692–744. 10.1128/MMBR.68.4.692-744.2004. 15590781 PMC539010

[160] Wells TJ, Totsika M, Schembri MA. 2010. Autotransporters of *Escherichia coli*: a sequence-based characterization. Microbiology (Reading) 156:2459–2469. 10.1099/mic.0.039024-0. 20447993

[161] Isberg RR, Voorhis DL, Falkow S. 1987. Identification of invasin: a protein that allows enteric bacteria to penetrate cultured mammalian cells. Cell 50:769–778. 10.1016/0092-8674(87)90335-7. 3304658

[162] Haltner E, Easson JH, Lehr C-M. 1997. Lectins and bacterial invasion factors for controlling endo- and transcytosis of bioadhesive drug carrier systems. Eur J Pharm Biopharm 44:3–13. 10.1016/S0939-6411(97)00096-9.

[163] Roche AJ, McFadden JP, Owen P. 2001. Antigen 43, the major phase-variable protein of the *Escherichia coli* outer membrane, can exist as a family of proteins encoded by multiple alleles. Microbiology 147:161–169. 10.1099/00221287-147-1-161. 11160810

[164] Ulett GC, Valle J, Beloin C, Sherlock O, Ghigo J-M, Schembri MA. 2007. Functional analysis of antigen 43 in uropathogenic *Escherichia coli* reveals a role in long-term persistence in the urinary tract. Infect Immun 75:3233–3244. 10.1128/IAI.01952-06. 17420234 PMC1932929

[165] Wells TJ, Sherlock O, Rivas L, Mahajan A, Beatson SA, Torpdahl M, Webb RI, Allsopp LP, Gobius KS, Gally DL, Schembri MA. 2008. EhaA is a novel autotransporter protein of enterohemorrhagic *Escherichia coli* O157:H7 that contributes to adhesion and biofilm formation. Environ Microbiol 10:589–604. 10.1111/j.1462-2920.2007.01479.x. 18237301

[166] Paxman JJ, Lo AW, Sullivan MJ, Panjikar S, Kuiper M, Whitten AE, Wang G, Luan C-H, Moriel DG, Tan L, Peters KM, Phan M-D, Gee CL, Ulett GC, Schembri MA, Heras B. 2019. Unique structural features of a bacterial autotransporter adhesin suggest mechanisms for interaction with host macromolecules. Nat Commun 10:1967. 10.1038/s41467-019-09814-6. 31036849 PMC6488583

[167] Allsopp LP, Beloin C, Ulett GC, Valle J, Totsika M, Sherlock O, Ghigo J-M, Schembri MA. 2012. Molecular characterization of UpaB and UpaC, two new autotransporter proteins of uropathogenic *Escherichia coli* CFT073. Infect Immun 80:321–332. 10.1128/IAI.05322-11. 21930758 PMC3255655

[168] Allsopp LP, Totsika M, Tree JJ, Ulett GC, Mabbett AN, Wells TJ, Kobe B, Beatson SA, Schembri MA. 2010. UpaH is a newly identified autotransporter protein that contributes to biofilm formation and bladder colonization by uropathogenic *Escherichia coli* CFT073. Infect Immun 78:1659–1669. 10.1128/IAI.01010-09. 20145097 PMC2849410

[169] Allsopp LP, Beloin C, Moriel DG, Totsika M, Ghigo J-M, Schembri MA. 2012. Functional heterogeneity of the UpaH autotransporter protein from uropathogenic *Escherichia coli*. J Bacteriol 194:5769–5782. 10.1128/JB.01264-12. 22904291 PMC3486098

[170] Valle J, Mabbett AN, Ulett GC, Toledo-Arana A, Wecker K, Totsika M, Schembri MA, Ghigo J-M, Beloin C. 2008. UpaG, a new member of the trimeric autotransporter family of adhesins in uropathogenic *Escherichia coli*. J Bacteriol 190:4147–4161. 10.1128/JB.00122-08. 18424525 PMC2446758

[171] Totsika M, Wells TJ, Beloin C, Valle J, Allsopp LP, King NP, Ghigo J-M, Schembri MA. 2012. Molecular characterization of the EhaG and UpaG trimeric autotransporter proteins from pathogenic *Escherichia coli*. Appl Environ Microbiol 78:2179–2189. 10.1128/AEM.06680-11. 22286983 PMC3302597

[172] Stein M, Kenny B, Stein MA, Finlay BB. 1996. Characterization of EspC, a 110-kilodalton protein secreted by enteropathogenic *Escherichia coli* which is homologous to members of the immunoglobulin A protease-like family of secreted proteins. J Bacteriol 178:6546–6554. 10.1128/jb.178.22.6546-6554.1996. 8932311 PMC178541

[173] Mellies JL, Navarro-Garcia F, Okeke I, Frederickson J, Nataro JP, Kaper JB. 2001. espC pathogenicity island of enteropathogenic *Escherichia coli* encodes an enterotoxin. Infect Immun 69:315–324. 10.1128/IAI.69.1.315-324.2001. 11119520 PMC97886

[174] Stathopoulos C, Provence DL, Curtiss R. 1999. Characterization of the avian pathogenic *Escherichia coli* hemagglutinin Tsh, a member of the immunoglobulin A protease-type family of autotransporters. Infect Immun 67:772–781. 10.1128/IAI.67.2.772-781.1999. 9916089 PMC96385

[175] Dozois CM, Dho-Moulin M, Brée A, Fairbrother JM, Desautels C, Curtiss R. 2000. Relationship between the Tsh autotransporter and pathogenicity of avian *Escherichia coli* and localization and analysis of the *tsh* genetic region. Infect Immun 68:4145–4154. 10.1128/iai.68.7.4145-4154.2000. 10858231 PMC101714

[176] Vijayakumar V, Santiago A, Smith R, Smith M, Robins-Browne RM, Nataro JP, Ruiz-Perez F. 2014. Role of class 1 serine protease autotransporter in the pathogenesis of *Citrobacter rodentium* colitis. Infect Immun 82:2626–2636. 10.1128/IAI.01518-13. 24711562 PMC4019172

[177] Celik N, Webb CT, Leyton DL, Holt KE, Heinz E, Gorrell R, Kwok T, Naderer T, Strugnell RA, Speed TP, Teasdale RD, Likić VA, Lithgow T. 2012. A bioinformatic strategy for the detection, classification and analysis of bacterial autotransporters. PLoS One 7:e43245. 10.1371/journal.pone.0043245. 22905239 PMC3419190

[178] Hart E, Yang J, Tauschek M, Kelly M, Wakefield MJ, Frankel G, Hartland EL, Robins-Browne RM. 2008. RegA, an AraC-like protein, is a global transcriptional regulator that controls virulence gene expression in *Citrobacter rodentium*. Infect Immun 76:5247–5256. 10.1128/IAI.00770-08. 18765720 PMC2573378

[179] Nichols KB, Totsika M, Moriel DG, Lo AW, Yang J, Wurpel DJ, Rossiter AE, Strugnell RA, Henderson IR, Ulett GC, Beatson SA, Schembri MA. 2016. Molecular characterization of the vacuolating autotransporter toxin in uropathogenic *Escherichia coli*. J Bacteriol 198:1487–1498. 10.1128/JB.00791-15. 26858103 PMC4859599

[180] Otto BR, Sijbrandi R, Luirink J, Oudega B, Heddle JG, Mizutani K, Park S-Y, Tame JRH. 2005. Crystal structure of hemoglobin protease, a heme binding autotransporter protein from pathogenic *Escherichia coli*. J Biol Chem 280:17339–17345. 10.1074/jbc.M412885200. 15728184

[181] Benjelloun-Touimi Z, Sansonetti PJ, Parsot C. 1995. SepA, the major extracellular protein of *Shigella flexneri*: autonomous secretion and involvement in tissue invasion. Mol Microbiol 17:123–135. 10.1111/j.1365-2958.1995.mmi_17010123.x. 7476198

[182] Guyer DM, Radulovic S, Jones F-E, Mobley HLT. 2002. Sat, the secreted autotransporter toxin of uropathogenic *Escherichia coli*, is a vacuolating cytotoxin for bladder and kidney epithelial cells. Infect Immun 70:4539–4546. 10.1128/IAI.70.8.4539-4546.2002. 12117966 PMC128167

[183] Guyer DM, Henderson IR, Nataro JP, Mobley HLT. 2000. Identification of Sat, an autotransporter toxin produced by uropathogenic *Escherichia coli*. Mol Microbiol 38:53–66. 10.1046/j.1365-2958.2000.02110.x. 11029690

[184] Nesta B, Spraggon G, Alteri C, Gomes Moriel D, Rosini R, Veggi D, Smith S, Bertoldi I, Pastorello I, Ferlenghi I, Fontana MR, Frankel G, Mobley HLT, Rappuoli R, Pizza M, Serino L, Soriani M. 2012. FdeC, a novel broadly conserved *Escherichia coli* adhesin eliciting protection against urinary tract infections. mBio 3:e00010-12. 10.1128/mBio.00010-12. 22496310 PMC3324786

[185] Valeri M, Rossi Paccani S, Kasendra M, Nesta B, Serino L, Pizza M, Soriani M. 2015. Pathogenic *Escherichia coli* exploits SslE mucinase activity to translocate through the mucosal barrier and get access to host cells. PLoS One 10:e0117486. 10.1371/journal.pone.0117486. 25789808 PMC4366376

[186] Moriel DG, Bertoldi I, Spagnuolo A, Marchi S, Rosini R, Nesta B, Pastorello I, Corea VAM, Torricelli G, Cartocci E, Savino S, Scarselli M, Dobrindt U, Hacker J, Tettelin H, Tallon LJ, Sullivan S, Wieler LH, Ewers C, Pickard D, Dougan G, Fontana MR, Rappuoli R, Pizza M, Serino L. 2010. Identification of protective and broadly conserved vaccine antigens from the genome of extraintestinal pathogenic *Escherichia coli*. Proc Natl Acad Sci U S A 107:9072–9077. 10.1073/pnas.0915077107. 20439758 PMC2889118

[187] Slater SL, Frankel G. 2020. Advances and challenges in studying type III secretion effectors of attaching and effacing pathogens. Front Cell Infect Microbiol 10:337. 10.3389/fcimb.2020.00337. 32733819 PMC7358347

[188] Sheikh A, Luo Q, Roy K, Shabaan S, Kumar P, Qadri F, Fleckenstein JM. 2014. Contribution of the highly conserved EaeH surface protein to enterotoxigenic *Escherichia coli* pathogenesis. Infect Immun 82:3657–3666. 10.1128/IAI.01890-14. 24935979 PMC4187836

[189] Kansal R, Rasko DA, Sahl JW, Munson GP, Roy K, Luo Q, Sheikh A, Kuhne KJ, Fleckenstein JM. 2013. Transcriptional modulation of enterotoxigenic *Escherichia coli* virulence genes in response to epithelial cell interactions. Infect Immun 81:259–270. 10.1128/IAI.00919-12. 23115039 PMC3536156

[190] Batisson I, Guimond M-P, Girard F, An H, Zhu C, Oswald E, Fairbrother JM, Jacques M, Harel J. 2003. Characterization of the novel factor Paa involved in the early steps of the adhesion mechanism of attaching and effacing *Escherichia coli*. Infect Immun 71:4516–4525. 10.1128/iai.71.8.4516-4525.2003. 12874331 PMC166039

[191] Li T, Han R, Wang Q, Wang S, Fang H, Li Z, Tu W, Wang D, Wang H. 2013. Immunogenicity of recombinant porcine attaching and effacing-associated protein compared with intimin fragment in *Escherichia coli* O157:H7-infected mice. Foodborne Pathog Dis 10:1016–1022. 10.1089/fpd.2013.1496. 24093308

[192] Germon P, Chen Y-H, He L, Blanco JE, Brée A, Schouler C, Huang S-H, Moulin-Schouleur M. 2005. *ibeA*, a virulence factor of avian pathogenic *Escherichia coli*. Microbiology 151:1179–1186. 10.1099/mic.0.27809-0. 15817785

[193] Reidl J, Boos W. 1991. The *malX malY* operon of *Escherichia coli* encodes a novel enzyme II of the phosphotransferase system recognizing glucose and maltose and an enzyme abolishing the endogenous induction of the maltose system. J Bacteriol 173:4862–4876. 10.1128/jb.173.15.4862-4876.1991. 1856179 PMC208166

[194] Johnson JR, Oswald E, O’Bryan TT, Kuskowski MA, Spanjaard L. 2002. Phylogenetic distribution of virulence-associated genes among *Escherichia coli* isolates associated with neonatal bacterial meningitis in The Netherlands. J Infect Dis 185:774–784. 10.1086/339343. 11920295

[195] Sannes MR, Kuskowski MA, Owens K, Gajewski A, Johnson JR. 2004. Virulence factor profiles and phylogenetic background of *Escherichia coli* isolates from veterans with bacteremia and uninfected control subjects. J Infect Dis 190:2121–2128. 10.1086/425984. 15551210

[196] Johnson JR, Jelacic S, Schoening LM, Clabots C, Shaikh N, Mobley HLT, Tarr PI. 2005. The IrgA homologue adhesin Iha is an *Escherichia coli* virulence factor in murine urinary tract infection. IAI 73:965–971. 10.1128/IAI.73.2.965-971.2005. PMC546986 15664939

[197] Fagan RP, Lambert MA, Smith SGJ. 2008. The Hek outer membrane protein of *Escherichia coli* strain RS218 binds to proteoglycan and utilizes a single extracellular loop for adherence, invasion, and autoaggregation. Infect Immun 76:1135–1142. 10.1128/IAI.01327-07. 18160475 PMC2258800

[198] Fagan RP, Smith SGJ. 2007. The Hek outer membrane protein of *Escherichia coli* is an auto-aggregating adhesin and invasin. FEMS Microbiol Lett 269:248–255. 10.1111/j.1574-6968.2006.00628.x. 17241243

[199] Binns MM, Davies DL, Hardy KG. 1979. Cloned fragments of the plasmid ColV,I-K94 specifying virulence and serum resistance. Nature 279:778–781. 10.1038/279778a0. 377103

[200] Binns MM, Mayden J, Levine RP. 1982. Further characterization of complement resistance conferred on *Escherichia coli* by the plasmid genes *traT* of R100 and *iss* of ColV,I-K94. Infect Immun 35:654–659. 10.1128/IAI.35.2.654-659.1982. 7035371 PMC351091

[201] Lynne AM, Foley SL, Nolan LK. 2006. Immune response to recombinant *Escherichia coli* Iss protein in poultry. Avian Dis 50:273–276. 10.1637/7441-092105R.1. 16863080

[202] Lynne AM, Skyberg JA, Logue CM, Nolan LK. 2007. Detection of Iss and Bor on the surface of *Escherichia coli*. J Appl Microbiol 102:660–666. 10.1111/j.1365-2672.2006.03133.x. 17309614

[203] Johnson TJ, Wannemuehler YM, Nolan LK. 2008. Evolution of the *iss* gene in *Escherichia coli*. Appl Environ Microbiol 74:2360–2369. 10.1128/AEM.02634-07. 18281426 PMC2293169

[204] Picard B, Garcia JS, Gouriou S, Duriez P, Brahimi N, Bingen E, Elion J, Denamur E. 1999. The link between phylogeny and virulence in *Escherichia coli* extraintestinal infection. Infect Immun 67:546–553. 10.1128/IAI.67.2.546-553.1999. 9916057 PMC96353

[205] Olier M, Marcq I, Salvador-Cartier C, Secher T, Dobrindt U, Boury M, Bacquié V, Penary M, Gaultier E, Nougayrède J-P, Fioramonti J, Oswald E. 2012. Genotoxicity of *Escherichia coli* Nissle 1917 strain cannot be dissociated from its probiotic activity. Gut Microbes 3:501–509. 10.4161/gmic.21737. 22895085 PMC3495787

[206] Schultz M. 2008. Clinical use of *Escherichia coli* Nissle 1917 in inflammatory bowel disease. Inflamm Bowel Dis 14:1012–1018. 10.1002/ibd.20377. 18240278

[207] Escobar-Páramo P, Grenet K, Le Menac’h A, Rode L, Salgado E, Amorin C, Gouriou S, Picard B, Rahimy MC, Andremont A, Denamur E, Ruimy R. 2004. Large-scale population structure of human commensal *Escherichia coli* isolates. Appl Environ Microbiol 70:5698–5700. 10.1128/AEM.70.9.5698-5700.2004. 15345464 PMC520916

[208] Duriez P, Clermont O, Bonacorsi S, Bingen E, Chaventré A, Elion J, Picard B, Denamur E. 2001. Commensal *Escherichia coli* isolates are phylogenetically distributed among geographically distinct human populations. Microbiology (Reading) 147:1671–1676. 10.1099/00221287-147-6-1671. 11390698

[209] Gomez A, Petrzelkova KJ, Burns MB, Yeoman CJ, Amato KR, Vlckova K, Modry D, Todd A, Jost Robinson CA, Remis MJ, Torralba MG, Morton E, Umaña JD, Carbonero F, Gaskins HR, Nelson KE, Wilson BA, Stumpf RM, White BA, Leigh SR, Blekhman R. 2016. Gut microbiome of coexisting BaAka pygmies and Bantu reflects gradients of traditional subsistence patterns. Cell Rep 14:2142–2153. 10.1016/j.celrep.2016.02.013. 26923597

[210] Lescat M, Clermont O, Woerther PL, Glodt J, Dion S, Skurnik D, Djossou F, Dupont C, Perroz G, Picard B, Catzeflis F, Andremont A, Denamur E. 2013. Commensal *Escherichia coli* strains in Guiana reveal a high genetic diversity with host-dependent population structure. Environ Microbiol Rep 5:49–57. 10.1111/j.1758-2229.2012.00374.x. 23757130

[211] Toh H, Oshima K, Toyoda A, Ogura Y, Ooka T, Sasamoto H, Park S-H, Iyoda S, Kurokawa K, Morita H, Itoh K, Taylor TD, Hayashi T, Hattori M. 2010. Complete genome sequence of the wild-type commensal *Escherichia coli* strain SE15, belonging to phylogenetic group B2. J Bacteriol 192:1165–1166. 10.1128/JB.01543-09. 20008064 PMC2812965

[212] Forde BM, Ben Zakour NL, Stanton-Cook M, Phan M-D, Totsika M, Peters KM, Chan KG, Schembri MA, Upton M, Beatson SA. 2014. The complete genome sequence of *Escherichia coli* EC958: a high-quality reference sequence for the globally disseminated multidrug-resistant *E. coli* O25b:H4-ST131 clone. PLoS One 9:e104400. 10.1371/journal.pone.0104400. 25126841 PMC4134206

[213] Avasthi TS, Kumar N, Baddam R, Hussain A, Nandanwar N, Jadhav S, Ahmed N. 2011. Genome of multidrug-resistant uropathogenic *Escherichia coli* strain NA114 from India. J Bacteriol 193:4272–4273. 10.1128/JB.05413-11. 21685291 PMC3147708

[214] Lewis AJ, Richards AC, Mulvey MA. 2016. Invasion of host cells and tissues by uropathogenic bacteria. Microbiol Spectr 4:10.1128/microbiolspec.UTI-0026-2016. 10.1128/microbiolspec.UTI-0026-2016. PMC5244466 28087946

[215] Wijetunge DSS, Gongati S, DebRoy C, Kim KS, Couraud PO, Romero IA, Weksler B, Kariyawasam S. 2015. Characterizing the pathotype of neonatal meningitis causing *Escherichia coli* (NMEC). BMC Microbiol 15:211. 10.1186/s12866-015-0547-9. 26467858 PMC4606507

[216] Camprubí-Font C, Ewers C, Lopez-Siles M, Martinez-Medina M. 2019. Genetic and phenotypic features to screen for putative adherent-invasive *Escherichia coli*. Front Microbiol 10. 10.3389/fmicb.2019.00108 PMC6393329 30846972

[217] Palmela C, Chevarin C, Xu Z, Torres J, Sevrin G, Hirten R, Barnich N, Ng SC, Colombel J-F. 2018. Adherent-invasive *Escherichia coli* in inflammatory bowel disease. Gut 67:574–587. 10.1136/gutjnl-2017-314903. 29141957

[218] Low D, Tran HT, Lee I, Dreux N, Kamba A, Reinecker H, Darfeuille–Michaud A, Barnich N, Mizoguchi E. 2013. Chitin-binding domains of *Escherichia coli* ChiA mediate interactions with intestinal epithelial cells in mice with colitis. Gastroenterology 145:602–612.e9. 10.1053/j.gastro.2013.05.017. 23684751 PMC3755095

[219] Nobles CL, Green SI, Maresso AW. 2013. A product of heme catabolism modulates bacterial function and survival. PLoS Pathog 9:e1003507. 10.1371/journal.ppat.1003507. 23935485 PMC3723568

[220] Adachi JA, Ericsson CD, Jiang Z, DuPont MW, Pallegar SR, DuPont HL. 2002. Natural history of enteroaggregative and enterotoxigenic *Escherichia coli* infection among US travelers to Guadalajara, Mexico. J Infect Dis 185:1681–1683. 10.1086/340419. 12023779

[221] Grad YH, Godfrey P, Cerquiera GC, Mariani-Kurkdjian P, Gouali M, Bingen E, Shea TP, Haas BJ, Griggs A, Young S, Zeng Q, Lipsitch M, Waldor MK, Weill F-X, Wortman JR, Hanage WP. 2013. Comparative genomics of recent Shiga toxin-producing *Escherichia coli* O104:H4: short-term evolution of an emerging pathogen. mBio 4 10.1128/mBio.00452-12. PMC3551546 23341549

[222] Qadri F, Svennerholm A-M, Faruque ASG, Sack RB. 2005. Enterotoxigenic *Escherichia coli* in developing countries: epidemiology, microbiology, clinical features, treatment, and prevention. Clin Microbiol Rev 18:465–483. 10.1128/CMR.18.3.465-483.2005. 16020685 PMC1195967

[223] Larsen MV, Cosentino S, Rasmussen S, Friis C, Hasman H, Marvig RL, Jelsbak L, Sicheritz-Ponten T, Ussery DW, Aarestrup FM, Lund O, Sicheritz-Pontén T, Ussery DW, Aarestrup FM, Lund O. 2012. Multilocus sequence typing of total-genome-sequenced bacteria. J Clin Microbiol 50:1355–1361. 10.1128/JCM.06094-11. 22238442 PMC3318499

[224] Wirth T, Falush D, Lan R, Colles F, Mensa P, Wieler LH, Karch H, Reeves PR, Maiden MCJ, Ochman H, Achtman M. 2006. Sex and virulence in *Escherichia coli*: an evolutionary perspective. Mol Microbiol 60:1136–1151. 10.1111/j.1365-2958.2006.05172.x. 16689791 PMC1557465

[225] Joensen KG, Tetzschner AMM, Iguchi A, Aarestrup FM, Scheutz F. 2015. Rapid and easy in silico serotyping of *Escherichia coli* isolates by use of whole-genome sequencing data. J Clin Microbiol 53:2410–2426. 10.1128/JCM.00008-15. 25972421 PMC4508402

[226] Roer L, Tchesnokova V, Allesøe R, Muradova M, Chattopadhyay S, Ahrenfeldt J, Thomsen MCF, Lund O, Hansen F, Hammerum AM, Sokurenko E, Hasman H. 2017. Development of a web tool for *Escherichia coli* subtyping based on *fimH* alleles. J Clin Microbiol 55:2538–2543. 10.1128/JCM.00737-17. 28592545 PMC5527432

[227] Kurylo CM, Alexander N, Dass RA, Parks MM, Altman RA, Vincent CT, Mason CE, Blanchard SC. 2016. Genome sequence and analysis of *Escherichia coli* MRE600, a colicinogenic, nonmotile strain that lacks RNase I and the type I methyltransferase, EcoKI. Genome Biol Evol 8:742–752. 10.1093/gbe/evw008. 26802429 PMC4825418

[228] Jeong H, Barbe V, Lee CH, Vallenet D, Yu DS, Choi S-H, Couloux A, Lee S-W, Yoon SH, Cattolico L, Hur C-G, Park H-S, Ségurens B, Kim SC, Oh TK, Lenski RE, Studier FW, Daegelen P, Kim JF. 2009. Genome sequences of *Escherichia coli* B strains REL606 and BL21(DE3). J Mol Biol 394:644–652. 10.1016/j.jmb.2009.09.052. 19786035

[229] Segura A, Auffret P, Klopp C, Bertin Y, Forano E. 2017. Draft genome sequence and characterization of commensal *Escherichia coli* strain BG1 isolated from bovine gastro-intestinal tract. Stand Genomic Sci 12:61. 10.1186/s40793-017-0272-0. 29046740 PMC5634895

[230] Oshima K, Toh H, Ogura Y, Sasamoto H, Morita H, Park S-H, Ooka T, Iyoda S, Taylor TD, Hayashi T, Itoh K, Hattori M. 2008. Complete genome sequence and comparative analysis of the wild-type commensal *Escherichia coli* strain SE11 isolated from a healthy adult. DNA Res 15:375–386. 10.1093/dnares/dsn026. 18931093 PMC2608844

[231] Wei J, Goldberg MB, Burland V, Venkatesan MM, Deng W, Fournier G, Mayhew GF, Plunkett G, Rose DJ, Darling A, Mau B, Perna NT, Payne SM, Runyen-Janecky LJ, Zhou S, Schwartz DC, Blattner FR. 2003. Complete genome sequence and comparative genomics of *Shigella flexneri* serotype 2a strain 2457T. Infect Immun 71:2775–2786. 10.1128/iai.71.5.2775-2786.2003. 12704152 PMC153260

[232] Reister M, Hoffmeier K, Krezdorn N, Rotter B, Liang C, Rund S, Dandekar T, Sonnenborn U, Oelschlaeger TA. 2014. Complete genome sequence of the Gram-negative probiotic *Escherichia coli* strain Nissle 1917. J Biotechnol 187:106–107. 10.1016/j.jbiotec.2014.07.442. 25093936

[233] Touchon M, Hoede C, Tenaillon O, Barbe V, Baeriswyl S, Bidet P, Bingen E, Bonacorsi S, Bouchier C, Bouvet O, Calteau A, Chiapello H, Clermont O, Cruveiller S, Danchin A, Diard M, Dossat C, El Karoui M, Frapy E, Garry L, Ghigo JM, Gilles AM, Johnson J, Le Bouguénec C, Lescat M, Mangenot S, Martinez-Jéhanne V, Matic I, Nassif X, Oztas S, Petit MA, Pichon C, Rouy Z, Saint Ruf C, Schneider D, Tourret J, Vacherie B, Vallenet D, Médigue C, Rocha EPC, Denamur E. 2009. Organized genome dynamics in the *Escherichia coli* species results in highly diverse adaptive paths. PLoS Genet 5:e1000344. 10.1371/journal.pgen.1000344. 19165319 PMC2617782

[234] Clermont O, Lescat M, O’Brien CL, Gordon DM, Tenaillon O, Denamur E. 2008. Evidence for a human-specific *Escherichia coli* clone. Environ Microbiol 10:1000–1006. 10.1111/j.1462-2920.2007.01520.x. 18177373

[235] Stephens CM, Skerker JM, Sekhon MS, Arkin AP, Riley LW. 2015. Complete genome sequences of four *Escherichia coli* ST95 isolates from bloodstream infections. Genome Announc 3:e01241-15. 10.1128/genomeA.01241-15. 26543109 PMC4645194

[236] Johnson TJ, Danzeisen JL, Youmans B, Case K, Llop K, Munoz-Aguayo J, Flores-Figueroa C, Aziz M, Stoesser N, Sokurenko E, Price LB, Johnson JR. 2016. Separate F-type plasmids have shaped the evolution of the H30 subclone of *Escherichia coli* sequence type 131. mSphere 1:e00121-16. 10.1128/mSphere.00121-16. 27390780 PMC4933990

[237] Stoesser N, Sheppard AE, Peirano G, Sebra R, Lynch T, Anson L, Kasarskis A, Motyl MR, Crook DW, Pitout JD. 2016. Complete sequencing of plasmids containing *bla*_OXA-163_ and *bla*_OXA-48_ in *Escherichia coli* sequence type 131. Antimicrob Agents Chemother 60:6948–6951. 10.1128/AAC.01130-16. 27600043 PMC5075115

[238] Peirano G, Bradford PA, Kazmierczak KM, Badal RE, Hackel M, Hoban DJ, Pitout JDD. 2014. Global incidence of carbapenemase-producing *Escherichia coli* ST131. Emerg Infect Dis 20:1928–1931. 10.3201/eid2011.141388. 25340464 PMC4214325

[239] Johnson TJ, Aziz M, Liu CM, Sokurenko E, Kisiela DI, Paul S, Andersen P, Johnson JR, Price LB. 2016. Complete genome sequence of a CTX-M-15-producing *Escherichia coli* strain from the H30Rx subclone of sequence type 131 from a patient with recurrent urinary tract infections, closely related to a lethal urosepsis isolate from the patient’s sister. Genome Announc 4:e00334-16. 10.1128/genomeA.00334-16.. 27174264 PMC4866839

[240] Johnson TJ, Hargreaves M, Shaw K, Snippes P, Lynfield R, Aziz M, Price LB. 2015. Complete genome sequence of a carbapenem-resistant extraintestinal pathogenic *Escherichia coli* strain belonging to the sequence type 131 H30R subclade. Genome Announc 3:e00272-15. 10.1128/genomeA.00272-15.. 25858844 PMC4392156

[241] Lescat M, Calteau A, Hoede C, Barbe V, Touchon M, Rocha E, Tenaillon O, Médigue C, Johnson JR, Denamur E. 2009. A module located at a chromosomal integration hot spot is responsible for the multidrug resistance of a reference strain from *Escherichia coli* clonal group A. Antimicrob Agents Chemother 53:2283–2288. 10.1128/AAC.00123-09. 19364861 PMC2687200

[242] Roos V, Nielsen EM, Klemm P. 2006. Asymptomatic bacteriuria *Escherichia coli* strains: adhesins, growth and competition. FEMS Microbiol Lett 262:22–30. 10.1111/j.1574-6968.2006.00355.x. 16907735

[243] Mehershahi KS, Abraham SN, Chen SL. 2015. Complete genome sequence of uropathogenic *Escherichia coli* strain CI5. Genome Announc 3:e00558-15. 10.1128/genomeA.00558-15. 26021932 PMC4447917

[244] Andersson P, Engberg I, Lidin-Janson G, Lincoln K, Hull R, Hull S, Svanborg C. 1991. Persistence of *Escherichia coli* bacteriuria is not determined by bacterial adherence. Infect Immun 59:2915–2921. 10.1128/IAI.59.9.2915-2921.1991. 1879917 PMC258113

[245] Mobley HL, Green DM, Trifillis AL, Johnson DE, Chippendale GR, Lockatell CV, Jones BD, Warren JW. 1990. Pyelonephritogenic *Escherichia coli* and killing of cultured human renal proximal tubular epithelial cells: role of hemolysin in some strains. Infect Immun 58:1281–1289. 10.1128/IAI.58.5.1281-1289.1990. 2182540 PMC258621

[246] Welch RA, Burland V, Plunkett G, Redford P, Roesch P, Rasko D, Buckles EL, Liou S-R, Boutin A, Hackett J, Stroud D, Mayhew GF, Rose DJ, Zhou S, Schwartz DC, Perna NT, Mobley HLT, Donnenberg MS, Blattner FR. 2002. Extensive mosaic structure revealed by the complete genome sequence of uropathogenic *Escherichia coli*. Proc Natl Acad Sci U S A 99:17020–17024. 10.1073/pnas.252529799. 12471157 PMC139262

[247] Chen SL, Hung C-S, Xu J, Reigstad CS, Magrini V, Sabo A, Blasiar D, Bieri T, Meyer RR, Ozersky P, Armstrong JR, Fulton RS, Latreille JP, Spieth J, Hooton TM, Mardis ER, Hultgren SJ, Gordon JI. 2006. Identification of genes subject to positive selection in uropathogenic strains of *Escherichia coli*: a comparative genomics approach. Proc Natl Acad Sci U S A 103:5977–5982. 10.1073/pnas.0600938103. 16585510 PMC1424661

[248] Mulvey MA, Schilling JD, Hultgren SJ. 2001. Establishment of a persistent *Escherichia coli* reservoir during the acute phase of a bladder infection. Infect Immun 69:4572–4579. 10.1128/IAI.69.7.4572-4579.2001. 11402001 PMC98534

[249] Hochhut B, Wilde C, Balling G, Middendorf B, Dobrindt U, Brzuszkiewicz E, Gottschalk G, Carniel E, Hacker J. 2006. Role of pathogenicity island-associated integrases in the genome plasticity of uropathogenic *Escherichia coli* strain 536. Mol Microbiol 61:584–595. 10.1111/j.1365-2958.2006.05255.x. 16879640

[250] Zurfluh K, Tasara T, Stephan R. 2016. Full-genome sequence of *Escherichia coli* K-15KW01, a uropathogenic *E. coli* B2 sequence type 127 isolate harboring a chromosomally carried bla CTX-M-15 gene. Genome Announc 4:e00927-16. 10.1128/genomeA.00927-16. 27587831 PMC5009988

[251] Hancock SJ, Phan M-D, Peters KM, Forde BM, Chong TM, Yin W-F, Chan K-G, Paterson DL, Walsh TR, Beatson SA, Schembri MA. 2017. Identification of IncA/C plasmid replication and maintenance genes and development of a plasmid multilocus sequence typing scheme. Antimicrob Agents Chemother 61:e01740-16. 10.1128/AAC.01740-16. 27872077 PMC5278728

[252] Achtman M, Mercer A, Kusecek B, Pohl A, Heuzenroeder M, Aaronson W, Sutton A, Silver RP. 1983. Six widespread bacterial clones among *Escherichia coli* K1 isolates. Infect Immun 39:315–335. 10.1128/IAI.39.1.315-335.1983. 6218094 PMC347943

[253] Silver RP, Aaronson W, Sutton A, Schneerson R. 1980. Comparative analysis of plasmids and some metabolic characteristics of *Escherichia coli* K1 from diseased and healthy individuals. Infect Immun 29:200–206. 10.1128/IAI.29.1.200-206.1980. 6995336 PMC551096

[254] Day MW, Jackson LA, Akins DR, Dyer DW, Chavez-Bueno S. 2015. Whole-genome sequences of the archetypal K1 *Escherichia coli* neonatal isolate RS218 and contemporary neonatal bacteremia clinical isolates SCB11, SCB12, and SCB15. Genome Announc 3:e01598-14. 10.1128/genomeA.01598-14.. 25720688 PMC4342429

[255] Wijetunge DSS, Katani R, Kapur V, Kariyawasam S. 2015. Complete genome sequence of *Escherichia coli* strain RS218 (O18:H7:K1), associated with neonatal meningitis. Genome Announc 3:e00804-15. 10.1128/genomeA.00804-15. 26205862 PMC4513156

[256] Nicholson BA, Wannemuehler YM, Logue CM, Li G, Nolan LK. 2016. Complete genome sequence of the neonatal meningitis-causing *Escherichia coli* strain NMEC O18. Genome Announc 4:e01239-16. 10.1128/genomeA.01239-16. 27811114 PMC5095484

[257] Tivendale KA, Logue CM, Kariyawasam S, Jordan D, Hussein A, Li G, Wannemuehler Y, Nolan LK. 2010. Avian-pathogenic *Escherichia coli* strains are similar to neonatal meningitis *E. coli* strains and are able to cause meningitis in the rat model of human disease. Infect Immun 78:3412–3419. 10.1128/IAI.00347-10. 20515929 PMC2916289

[258] Iqbal J, Dufendach KR, Wellons JC, Kuba MG, Nickols HH, Gómez-Duarte OG, Wynn JL. 2016. Lethal neonatal meningoencephalitis caused by multidrug-resistant, highly virulent *Escherichia coli*. Infect Dis (Auckl) 48:461–466. 10.3109/23744235.2016.1144142. PMC4818964 27030919

[259] Nielsen DW, Ricker N, Barbieri NL, Wynn JL, Gómez-Duarte OG, Iqbal J, Nolan LK, Allen HK, Logue CM. 2018. Complete genome sequence of the multidrug-resistant neonatal meningitis *Escherichia coli* serotype O75:H5:K1 strain mcjchv-1 (NMEC-O75). Microbiol Resour Announc 7:e01043-18. 10.1128/MRA.01043-18. 30533615 PMC6256591

[260] Lu S, Zhang X, Zhu Y, Kim KS, Yang J, Jin Q. 2011. Complete genome sequence of the neonatal-meningitis-associated *Escherichia coli* strain CE10. J Bacteriol 193:7005–7005. 10.1128/JB.06284-11. 22123760 PMC3232859

[261] Wang X, Wei L, Wang B, Zhang R, Liu C, Bi D, Chen H, Tan C. 2016. Complete genome sequence and characterization of avian pathogenic *Escherichia coli* field isolate ACN001. Stand Genomic Sci 11:13. 10.1186/s40793-015-0126-6. 26823959 PMC4730748

[262] Mangiamele P, Nicholson B, Wannemuehler Y, Seemann T, Logue CM, Li G, Tivendale KA, Nolan LK. 2013. Complete genome sequence of the avian pathogenic *Escherichia coli* strain APEC O78. Genome Announc 1:e0002613. 10.1128/genomeA.00026-13. 23516182 PMC3622999

[263] Huja S, Oren Y, Trost E, Brzuszkiewicz E, Biran D, Blom J, Goesmann A, Gottschalk G, Hacker J, Ron EZ, Dobrindt U. 2015. Genomic avenue to avian colisepticemia. mBio 6:e01681-14. 10.1128/mBio.01681-14. 25587010 PMC4313913

[264] Johnson TJ, Kariyawasam S, Wannemuehler Y, Mangiamele P, Johnson SJ, Doetkott C, Skyberg JA, Lynne AM, Johnson JR, Nolan LK. 2007. The genome sequence of avian pathogenic *Escherichia coli* strain O1:K1:H7 shares strong similarities with human extraintestinal pathogenic *E. coli* genomes. J Bacteriol 189:3228–3236. 10.1128/JB.01726-06. 17293413 PMC1855855

[265] Mangiamele P. 2014. From sequencing to analysis: building a comparative genomics tool for the biologist end-user. Iowa State University Digital Repository, Ames, IA.

[266] Nielsen DW, Mangiamele P, Ricker N, Barbieri NL, Allen HK, Nolan LK, Logue CM. 2018. Complete genome sequence of avian pathogenic *Escherichia coli* strain APEC O2-211. Microbiol Resour Announc 7:e01046-18. 10.1128/MRA.01046-18. 30533666 PMC6256689

[267] Li G, Laturnus C, Ewers C, Wieler LH. 2005. Identification of genes required for avian *Escherichia coli* septicemia by signature-tagged mutagenesis. Infect Immun 73:2818–2827. 10.1128/IAI.73.5.2818-2827.2005. 15845486 PMC1087346

[268] Zhu Ge X, Jiang J, Pan Z, Hu L, Wang S, Wang H, Leung FC, Dai J, Fan H. 2014. Comparative genomic analysis shows that avian pathogenic *Escherichia coli* isolate IMT5155 (O2:K1:H5; ST complex 95, ST140) shares close relationship with ST95 APEC O1:K1 and human ExPEC O18:K1 strains. PLoS One 9:e112048. 10.1371/journal.pone.0112048. 25397580 PMC4232414

[269] Miquel S, Peyretaillade E, Claret L, de Vallée A, Dossat C, Vacherie B, Zineb EH, Segurens B, Barbe V, Sauvanet P, Neut C, Colombel J-F, Medigue C, Mojica FJM, Peyret P, Bonnet R, Darfeuille-Michaud A. 2010. Complete genome sequence of Crohn’s disease-associated adherent-invasive *Escherichia coli* strain LF82. PLoS One 5:e12714. 10.1371/journal.pone.0012714. 20862302 PMC2941450

[270] Nash JH, Villegas A, Kropinski AM, Aguilar-Valenzuela R, Konczy P, Mascarenhas M, Ziebell K, Torres AG, Karmali MA, Coombes BK. 2010. Genome sequence of adherent-invasive *Escherichia coli* and comparative genomic analysis with other *E. coli* pathotypes. BMC Genomics 11:667. 10.1186/1471-2164-11-667. 21108814 PMC3091784

[271] Krause DO, Little AC, Dowd SE, Bernstein CN. 2011. Complete genome sequence of adherent invasive *Escherichia coli* UM146 isolated from ileal Crohn’s disease biopsy tissue. J Bacteriol 193:583–583. 10.1128/JB.01290-10. 21075930 PMC3019814

[272] Kim SC, Tonkonogy SL, Albright CA, Tsang J, Balish EJ, Braun J, Huycke MM, Sartor RB. 2005. Variable phenotypes of enterocolitis in interleukin-10-deficient mice monoassociated with two different commensal bacteria. Gastroenterology 128:891–906. 10.1053/j.gastro.2005.02.009. 15825073

[273] Ellermann M, Huh EY, Liu B, Carroll IM, Tamayo R, Sartor RB. 2015. Adherent-invasive *Escherichia coli* production of cellulose influences iron-induced bacterial aggregation, phagocytosis, and induction of colitis. Infect Immun 83:4068–4080. 10.1128/IAI.00904-15. 26216423 PMC4567620

[274] Ogura Y, Ooka T, Iguchi A, Toh H, Asadulghani M, Oshima K, Kodama T, Abe H, Nakayama K, Kurokawa K, Tobe T, Hattori M, Hayashi T. 2009. Comparative genomics reveal the mechanism of the parallel evolution of O157 and non-O157 enterohemorrhagic *Escherichia coli*. Proc Natl Acad Sci U S A 106:17939–17944. 10.1073/pnas.0903585106. 19815525 PMC2764950

[275] González-Escalona N, Allard MA, Brown EW, Sharma S, Hoffmann M. 2019. Nanopore sequencing for fast determination of plasmids, phages, virulence markers, and antimicrobial resistance genes in Shiga toxin-producing *Escherichia coli*. PLoS One 14:e0220494. 10.1371/journal.pone.0220494. 31361781 PMC6667211

[276] Lindsey RL, Rowe L, Garcia-Toledo L, Loparev V, Knipe K, Stripling D, Martin H, Trees E, Juieng P, Batra D, Strockbine N. 2016. High-quality draft genome sequences for five non-O157 Shiga toxin-producing *Escherichia coli* strains generated with PacBio sequencing and optical maps. Genome Announc 4:e00626-16. 10.1128/genomeA.00626-16. 27365352 PMC4929515

[277] Yan X, Fratamico PM, Bono JL, Baranzoni GM, Chen C-Y. 2015. Genome sequencing and comparative genomics provides insights on the evolutionary dynamics and pathogenic potential of different H-serotypes of Shiga toxin-producing *Escherichia coli* O104. BMC Microbiol 15:83. 10.1186/s12866-015-0413-9. 25887577 PMC4393859

[278] Cowley LA, Dallman TJ, Fitzgerald S, Irvine N, Rooney PJ, McAteer SP, Day M, Perry NT, Bono JL, Jenkins C, Gally DL. 2016. Short-term evolution of Shiga toxin-producing *Escherichia coli* O157:H7 between two food-borne outbreaks. Microb Genom 2:e000084. 10.1099/mgen.0.000084. 28348875 PMC5320650

[279] Oh D-H, Pan Y, Berry E, Cooley M, Mandrell R, Breidt F. 2009. *Escherichia coli* O157:H7 strains isolated from environmental sources differ significantly in acetic acid resistance compared with human outbreak strains. J Food Prot 72:503–509. 10.4315/0362-028X-72.3.503. 19343937

[280] Baranzoni GM, Fratamico PM, Reichenberger ER, Kim G-H, Breidt F, Kay K, Oh D-H. 2016. Complete genome sequences of *Escherichia coli* O157:H7 strains SRCC 1675 and 28RC, which vary in acid resistance. Genome Announc 4. 10.1128/genomeA.00743-16. PMC4966468 27469964

[281] Kudva IT, Hatfield PG, Hovde CJ. 1996. *Escherichia coli* O157:H7 in microbial flora of sheep. J Clin Microbiol 34:431–433. 10.1128/JCM.34.2.431-433.1996. 8789031 PMC228813

[282] Marques LRM, Moore MA, Wells JG, Wachsmuth IK, O’brien AD. 1986. Production of Shiga-like toxin by *Escherichia coli*. J Infect Dis 154:338–341. 10.1093/infdis/154.2.338. 3522760

[283] Eppinger M, Mammel MK, Leclerc JE, Ravel J, Cebula TA. 2011. Genomic anatomy of *Escherichia coli* O157:H7 outbreaks. Proc Natl Acad Sci U S A 108:20142–20147. 10.1073/pnas.1107176108. 22135463 PMC3250189

[284] Perna NT, Plunkett G, Burland V, Mau B, Glasner JD, Rose DJ, Mayhew GF, Evans PS, Gregor J, Kirkpatrick HA, Pósfai G, Hackett J, Klink S, Boutin A, Shao Y, Miller L, Grotbeck EJ, Davis NW, Lim A, Dimalanta ET, Potamousis KD, Apodaca J, Anantharaman TS, Lin J, Yen G, Schwartz DC, Welch RA, Blattner FR. 2001. Genome sequence of enterohaemorrhagic *Escherichia coli* O157:H7. Nature 409:529–533. 10.1038/35054089. 11206551

[285] Park D, Stanton E, Ciezki K, Parrell D, Bozile M, Pike D, Forst SA, Jeong KC, Ivanek R, Döpfer D, Kaspar CW. 2013. Evolution of the Stx2-encoding prophage in persistent bovine *Escherichia coli* O157:H7 strains. Appl Environ Microbiol 79:1563–1572. 10.1128/AEM.03158-12. 23275514 PMC3591979

[286] Shere JA, Bartlett KJ, Kaspar CW. 1998. Longitudinal study of *Escherichia coli* O157:H7 dissemination on four dairy farms in Wisconsin. Appl Environ Microbiol 64:1390–1399. 10.1128/AEM.64.4.1390-1399.1998. 9546176 PMC106160

[287] Teng L, Ginn A, Jeon S, Kang M, Jeong KC. 2016. Complete genome sequence of an *Escherichia coli* O157:H7 strain isolated from a super-shedder steer. Genome Announc 4:e00258-16. 10.1128/genomeA.00258-16. 27056233 PMC4824266

[288] Makino K, Ishii K, Yasunaga T, Hattori M, Yokoyama K, Yutsudo CH, Kubota Y, Yamaichi Y, Iida T, Yamamoto K, Honda T, Han CG, Ohtsubo E, Kasamatsu M, Hayashi T, Kuhara S, Shinagawa H. 1998. Complete nucleotide sequences of 93-kb and 3.3-kb plasmids of an enterohemorrhagic *Escherichia coli* O157:H7 derived from Sakai outbreak. DNA Res 5:1–9. 10.1093/dnares/5.1.1. 9628576

[289] Hayashi T. 2001. Complete genome sequence of enterohemorrhagic *Escherichia coli* O157:H7 and genomic comparison with a laboratory strain K-12. DNA Res 8:11–22. 10.1093/dnares/8.1.11. 11258796

[290] Katani R, Cote R, Raygoza Garay JA, Li L, Arthur TM, DebRoy C, Mwangi MM, Kapur V. 2015. Complete genome sequence of SS52, a strain of *Escherichia coli* O157:H7 recovered from supershedder cattle. Genome Announc 3:e01569-14. 10.1128/genomeA.01569-14. 25792068 PMC4395050

[291] Kulasekara BR, Jacobs M, Zhou Y, Wu Z, Sims E, Saenphimmachak C, Rohmer L, Ritchie JM, Radey M, McKevitt M, Freeman TL, Hayden H, Haugen E, Gillett W, Fong C, Chang J, Beskhlebnaya V, Waldor MK, Samadpour M, Whittam TS, Kaul R, Brittnacher M, Miller SI. 2009. Analysis of the genome of the *Escherichia coli* O157:H7 2006 spinach-associated outbreak isolate indicates candidate genes that may enhance virulence. Infect Immun 77:3713–3721. 10.1128/IAI.00198-09. 19564389 PMC2738036

[292] Xiong Y, Wang P, Lan R, Ye C, Wang H, Ren J, Jing H, Wang Y, Zhou Z, Bai X, Cui Z, Luo X, Zhao A, Wang Y, Zhang S, Sun H, Wang L, Xu J. 2012. A novel *Escherichia coli* O157:H7 clone causing a major hemolytic uremic syndrome outbreak in China. PLoS One 7:e36144. 10.1371/journal.pone.0036144. 22558360 PMC3338595

[293] Cooper KK, Mandrell RE, Louie JW, Korlach J, Clark TA, Parker CT, Huynh S, Chain PS, Ahmed S, Carter M. 2014. Comparative genomics of enterohemorrhagic *Escherichia coli* O145:H28 demonstrates a common evolutionary lineage with *Escherichia coli* O157:H7. BMC Genomics 15:17. 10.1186/1471-2164-15-17. 24410921 PMC3893438

[294] Scheutz F, Møller Nielsen E, Frimodt-Møller J, Boisen N, Morabito S, Tozzoli R, Nataro JP, Caprioli A. 2011. Characteristics of the enteroaggregative Shiga toxin/verotoxin-producing *Escherichia coli* O104:H4 strain causing the outbreak of haemolytic uraemic syndrome in Germany, May to June 2011. Eurosurveillance 16:19889. 10.2807/ese.16.24.19889-en. 21699770

[295] Ahmed SA, Awosika J, Baldwin C, Bishop-Lilly KA, Biswas B, Broomall S, Chain PSG, Chertkov O, Chokoshvili O, Coyne S, Davenport K, Detter JC, Dorman W, Erkkila TH, Folster JP, Frey KG, George M, Gleasner C, Henry M, Hill KK, Hubbard K, Insalaco J, Johnson S, Kitzmiller A, Krepps M, Lo C-C, Luu T, McNew LA, Minogue T, Munk CA, Osborne B, Patel M, Reitenga KG, Rosenzweig CN, Shea A, Shen X, Strockbine N, Tarr C, Teshima H, van Gieson E, Verratti K, Wolcott M, Xie G, Sozhamannan S, Gibbons HS. Threat Characterization Consortium. 2012. Genomic comparison of *Escherichia coli* O104:H4 isolates from 2009 and 2011 reveals plasmid, and prophage heterogeneity, including Shiga toxin encoding phage stx2. PLoS One 7:e48228. 10.1371/journal.pone.0048228. 23133618 PMC3486847

[296] Beaulaurier J, Zhang X-S, Zhu S, Sebra R, Rosenbluh C, Deikus G, Shen N, Munera D, Waldor MK, Chess A, Blaser MJ, Schadt EE, Fang G. 2015. Single molecule-level detection and long read-based phasing of epigenetic variations in bacterial methylomes. Nat Commun 6:7438. 10.1038/ncomms8438. 26074426 PMC4490391

[297] Strockbine NA, Faruque SM, Kay BA, Haider K, Alam K, Alam AN, Tzipori S, Wachsmuth IK. 1992. DNA probe analysis of diarrhoeagenic *Escherichia coli*: detection of EAF-positive isolates of traditional enteropathogenic *E. coli* serotypes among Bangladeshi paediatric diarrhoea patients. Mol Cell Probes 6:93–99. 10.1016/0890-8508(92)90052-y. 1513347

[298] Evans DJ, Evans DG. 1973. Three characteristics associated with enterotoxigenic *Escherichia coli* isolated from man. Infect Immun 8:322–328. 10.1128/IAI.8.3.322-328.1973. 4581006 PMC422851

[299] Shepard SM, Danzeisen JL, Isaacson RE, Seemann T, Achtman M, Johnson TJ. 2012. Genome sequences and phylogenetic analysis of K88- and F18-positive porcine enterotoxigenic *Escherichia coli*. J Bacteriol 194:395–405. 10.1128/JB.06225-11. 22081385 PMC3256668

[300] Sack D. 1975. Diarrhea associated with heat-stable enterotoxin-producing strains of *Escherichia coli*. Lancet 306:239–241. 10.1016/S0140-6736(75)90958-7. 49793

[301] Wachsmuth K, Wells J, Shipley P, Ryder R. 1979. Heat-labile enterotoxin production in isolates from a shipboard outbreak of human diarrheal illness. Infect Immun 24:793–797. 10.1128/IAI.24.3.793-797.1979. 381200 PMC414376

[302] Sahl JW, Rasko DA. 2012. Analysis of global transcriptional profiles of enterotoxigenic *Escherichia coli* isolate E24377A. Infect Immun 80:1232–1242. 10.1128/IAI.06138-11. 22215741 PMC3294641

[303] Levine MM, Nalin DR, Hoover DL, Bergquist EJ, Hornick RB, Young CR. 1979. Immunity to enterotoxigenic *Escherichia coli*. Infect Immun 23:729–736. 10.1128/IAI.23.3.729-736.1979. 378834 PMC414227

[304] Youssef M, Shurman A, Bougnoux M-E, Rawashdeh M, Bretagne S, Strockbine N. 2000. Bacterial, viral and parasitic enteric pathogens associated with acute diarrhea in hospitalized children from northern Jordan. FEMS Immunol Med Microbiol 28:257–263. 10.1111/j.1574-695X.2000.tb01485.x. 10865179

[305] Smith P, Lindsey RL, Rowe LA, Batra D, Stripling D, Garcia-Toledo L, Drapeau D, Knipe K, Strockbine N. 2018. High-quality whole-genome sequences for 21 enterotoxigenic *Escherichia coli* strains generated with PacBio sequencing. Genome Announc 6:e01311-18. 10.1128/genomeA.01311-17. 29326203 PMC5764927

[306] Iguchi A, Thomson NR, Ogura Y, Saunders D, Ooka T, Henderson IR, Harris D, Asadulghani M, Kurokawa K, Dean P, Kenny B, Quail MA, Thurston S, Dougan G, Hayashi T, Parkhill J, Frankel G. 2009. Complete genome sequence and comparative genome analysis of enteropathogenic *Escherichia coli* O127:H6 strain E2348/69. J Bacteriol 191:347–354. 10.1128/JB.01238-08. 18952797 PMC2612414

[307] Zhou Z, Li X, Liu B, Beutin L, Xu J, Ren Y, Feng L, Lan R, Reeves PR, Wang L. 2010. Derivation of *Escherichia coli* O157:H7 from its O55:H7 precursor. PLoS One 5:e8700. 10.1371/journal.pone.0008700. 20090843 PMC2806823

[308] Kyle JL, Cummings CA, Parker CT, Quinones B, Vatta P, Newton E, Huynh S, Swimley M, Degoricija L, Barker M, Fontanoz S, Nguyen K, Patel R, Fang R, Tebbs R, Petrauskene O, Furtado M, Mandrell RE. 2012. *Escherichia coli* serotype O55:H7 diversity supports parallel acquisition of bacteriophage at Shiga toxin phage insertion sites during evolution of the O157:H7 lineage. J Bacteriol 194:1885–1896. 10.1128/JB.00120-12. 22328665 PMC3318487

