# Supplementary material for: Comparative Pathogenomics of Escherichia coli: Polyvalent Vaccine Target Identification through Virulome Analysis
Source: Infect Immun. 2021 Jul 15;89(8):e00115-21. doi: 10.1128/IAI.00115-21 (PMC8281228; doi:10.1128/IAI.00115-21)
Supplement: Supplemental file 1 — Supplemental material. Download IAI00115-21_Supp_1_seq4.pdf, PDF file, 1.3 MB [file iai00115-21_supp_1_seq4.pdf]

Supporting Information

Supporting Appendix 1. Information About Phylogroup Characterization.

Phylogroup categorization was done using an in-house method inspired by Clermont Phylotyping, a routine lab multiplex PCR assay used to categorize isolates into phylogroups [1]. The method is based on the presence of amplicon fragments that create fragment patterns that are phylogroup-specific. The main quadruplex PCR assay uses four primer sets to detect: *arpA*, *chuA*, *yjaA*, and *TspE4C2*. The genotype obtained from using these primer sets can be related back to phylogroup. For example, many B2 strains show a (-/+/+/+) genotype that corresponds to (*arpA-/chuA+/yjaA+/TspE4C2+*). These are supplemented with additional allele specific primers, *trpA_gpC* and *arpA_gpE*, which allow one to resolve phylogroups C from A, and E from D, respectively [2]. A recently discovered phylogroup, phylogroup G, can be resolved from phylogroups B2 and F using primers for *ybgD* [3]. Using these primers, *in silico* PCR was done on strains from phylogroups that were known to contain the genes. Primer sequences and fragment sequences can be found in Supp Fig 1. The resulting fragments were aligned to strains using megaBLASTs to bin strains into hit vs no hit. In essence, our pathotype database was used to train and verify our method.

When categorizing phylogroups, some cases arose where the PCR fragments were present, but with mutations in the primer region that prevents them from being detected by PCR. In these cases, percent identity cutoffs had to be used to differentiate strains. The best example and most complicated example of this is *arpA*, which is supposedly absent in B2 and F strains. Our results, however, indicate that every strain contains this fragment. The reason the *arpA* fragment doesn't amplify in PCR assays is probably due to an AAGA>GCGG mutation in the reverse primer binding region. To differentiate between a "Hit" in other phylogroups and a "No Hit" in phylogroup B2, F, and G we took a 61 bp region of the original 400 bp *arpA* fragment that contained many of the point mutations seen in the B2, F, and G phylogroups. Alignment of this fragment with members of the B2 (ED1a, Nissle 1917, SE15, CD306, JJ1887, etc) and F phylogroups (IA39 and CE10) resulted in a percent identity <95.7%, while alignments to members from other phylogroups, including the D phylogroup that is closely related to the B2 and F phylogroups, resulted in a percent identity of 100% in most cases (see below). Using this as a guide, an E value cutoff of 97% was used when differentiating between Hit and No Hit in the phylogroup database. This is the only fragment where we did not use the whole fragment sequence. For the other targets, the full sequence length was used as there was easily identifiable percent identity values to differentiate phylogroups. These percent identities verified by using the autoMLST server to produce core genome phylogeny to verify which sequences clustered together.

After initial characterization of phylogroups, each strain in the phylogroups were aligned with the hypothetical PCR fragments used in the megaBLAST search of the characterization. This served two purposes. The first and main purpose was to look for mutations in the primer binding regions. The second was to verify that the mutational patterns for the fragments were largely consistent. If any strain showed a mutation in the primer binding region or a different SNP pattern, it was double-checked using both the EZClermont [4] and the ClermonTyping [5] software. Our results were largely consistent, with 100% of A, B1, C showing the expected patterns.

One place this did cause trouble was in the (-+-+;) genotype. These were all originally classified as B2 strains, but the alignments showed that there was a C>G mutation at the last 3' nucleotide of the

forward primer sequence. Both EZClermont and ClermonTyping classified these as phylogroup F strains instead of phylogroup B2. To verify this, we megaBLASTed all of the (-+-+) genotype strains against *ibeA* and all 17 strains that carried the C>G mutation lacked this gene. Given this, the *ibeA*- strains were reclassified as F phylogroup strains. Additionally, these 17 strains also carried the CFA fimbriae, supporting their inclusion in the F phylogroup.

Classification was more difficult with the D phylogroup. Starting with the *arpA*_gpE primer used to distinguish phylogroup D from phylogroup E, all of these strains contained this fragment since it is a allele specific primer set. However, many D strains that carried it had a G>A SNP in the reverse primer region and these strains were classified as phylogroup D by both EZClermont and ClermonTyping. Together, 8 out of the 67 strains originally classified as phylogroup D ended up being strains from phylogroup E. This was exclusively because mutations outside of the primer region, which made it hard to distinguish hit vs no hit based on percent identity alone. The only other strain that showed a possibly divergent pattern was BEC1-S17-ESBL-09 (AP022298.1). According to EZClermont, it belongs to the F phylogroup, but according to ClermonTyping, it belongs to the either the E or the D (mash) phylogroup. We left it characterized as a phylogroup D strain because it showed a SNP pattern similar in the hypothetical amplicon to other members of the phylogroup D.

Some phylogroup E strains did show mutations in the reverse primer of *arp*A_gpE. These were classified as strains from phylogroup D in both the EZClermont and ClermonTyping software, but the alternative mesh prediction in ClermonTyping categorized it as a strain from phylogroup E. These strains are O145:H28 EHEC strains, which have representatives in our phylogroup database in RM13514 and RM13516. Whole genome comparisons suggests that thee strains are closer to phylogroup E than to phylogroup D. With this in mind, we left these strains classified as phylogroup E [6].

Phylogroup G also frustrated attempts to easily classify it. Initially it was classified based on the presences of *ybgD* in (-+-+) and (-+--) genotypes [3]. However, of the 39 (-+-+) genotype strains that were *ybgD*+, 22 of them were classified as phylogroup D strains by both ClermonTyping and EZClermont. This is because, despite the having *arpA* primer sequences only having a single SNP, these 22 strains did not elicit a hit from our modified *arpA*_T_61 sequence because of mutations just outside of the primer binding sequence. In fact, they are the only strain originally classified as (-+-+) that did not—the others had a fragment hit but were below the percent identity cut off. The simplest way to distinguish the phylogroup G strains from these phylogroup D strains appears to be the *arpA*_gpE sequence. This sequence (though not the phylogroup E allele, so designed *arpA*_gpE* in Supp Fig 2) is only found in phylogroup D strains. List of strains with phylogroup assignment can be found in Supp Data 2)

To verify our phylogroup assignments, 20 strains from each phylogroup were chosen at random using Microsoft Excel 16 random number generator function (=RANDBETWEEN; duplicate numbers were rerolled), and these numbers were used to select strains from a list. These 140 strains were assigned to phylogroup using the stand-alone version of ClermonTyping (https://github.com/A-BN/ClermonTyping.git). Results for this verification can be found in supplemental data 3.

All of this information was used to enhance our ability to categorize strains and will be used in the future on the larger RefSeq database.

Supporting Figure 1

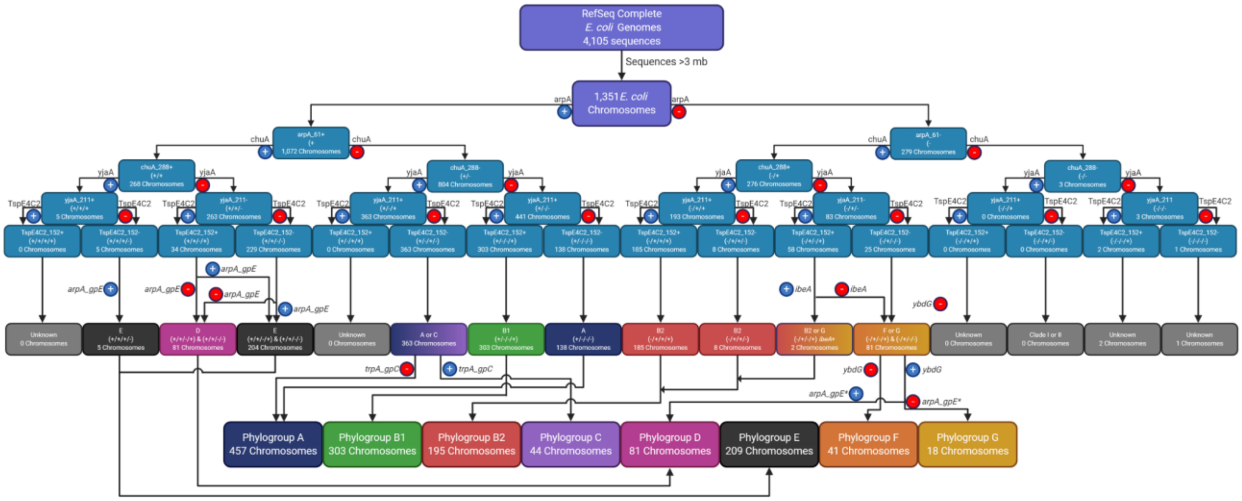

Supporting Figure 1: Construction of Phylogroup Database. RefSeq sequences designated as *E. coli* were downloaded. Of these 4,105 sequences, sequences shorter than 3 mbps were excluded in order to exclude plasmids. The remaining 1,351 presumptive *E. coli* genomes were then iteratively megaBLASTed against each fragment with an output binning sequences into hit vs no hit. By doing this for each fragment, a pattern emerged that could be used to designate phylogroup, similar to the PCR patterns read during Clermont Phylotyping. Made using Biorender.

Supporting Figure 2

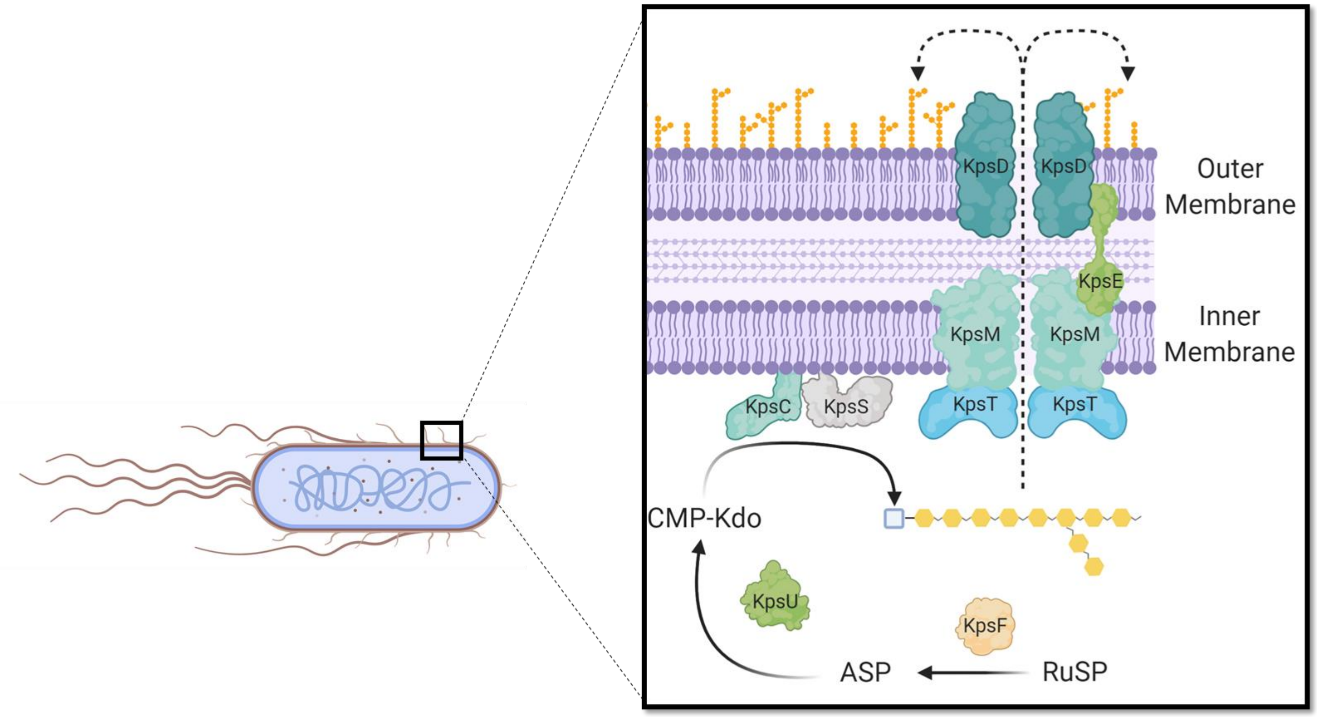

Supporting Figure 2: Group 2 and 3 Capsule Export. Visual representation of the export of group 2 and 3 capsule. Of these genes, *kpsC, kpsD, kpsE, kpsF, kpsS,* and *kpsU* are used by both group 2 and group 3 capsule. However, *kpsM* and *kps*T are specific for group 2 capsule and as such offer a convenient way to distinguish the two groups. Made using Biorender.

Supporting Figure 3

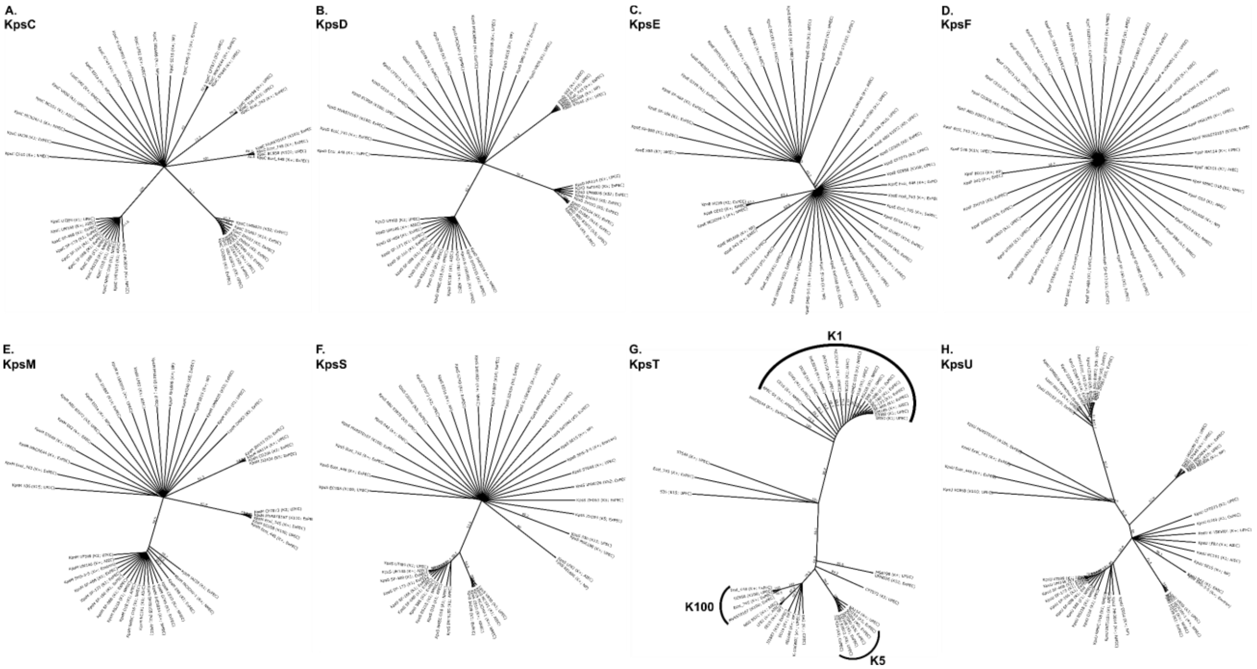

Supporting Figure 3: Relationship Between Kps Proteins. Trees were built from Geneious protein alignments using Geneious Tree Builder with bootstrap support from 1,000 replicates. Branch labels represent percent consensus support. Branches were transformed proportionally for easier comparison. Each panel shows results for a different protein. (A) KpsC: membrane bound β-Kdo transferase that extends the poly 3-deoxy-D-*manno*-oct-2-ulosonic acid (Kdo) linker of capsular polysaccharide [7]. (B) KpsD: Outer membrane capsular polysaccharide transport protein. (C) KpsE: inner membrane-associated periplasmic protein involved in capsular polysaccharide transport. (D) KpsF: arabinose 5-phosphate isomerase involved in biosynthesis of capsular polysaccharide. (E) KpsM: Transmembrane component of ABC transporter complex involved in capsular polysaccharide transport across the inner membrane. (F) KpsS: β-Kdo transferase that adds initial Kdo of the Kdo linker of capsular polysaccharide [7]. (G) KpsT ATP-binding component of the ABC transporter complex involved in capsular polysaccharide transport across the inner membrane. (H) KpsU: 3-deoxy-*manno*-octulosonate cytidylyltransferase (CMP-Kdo synthetase which allows for the Kdo linker to be embedded in the outer membrane [8].

Supprting Figure 4

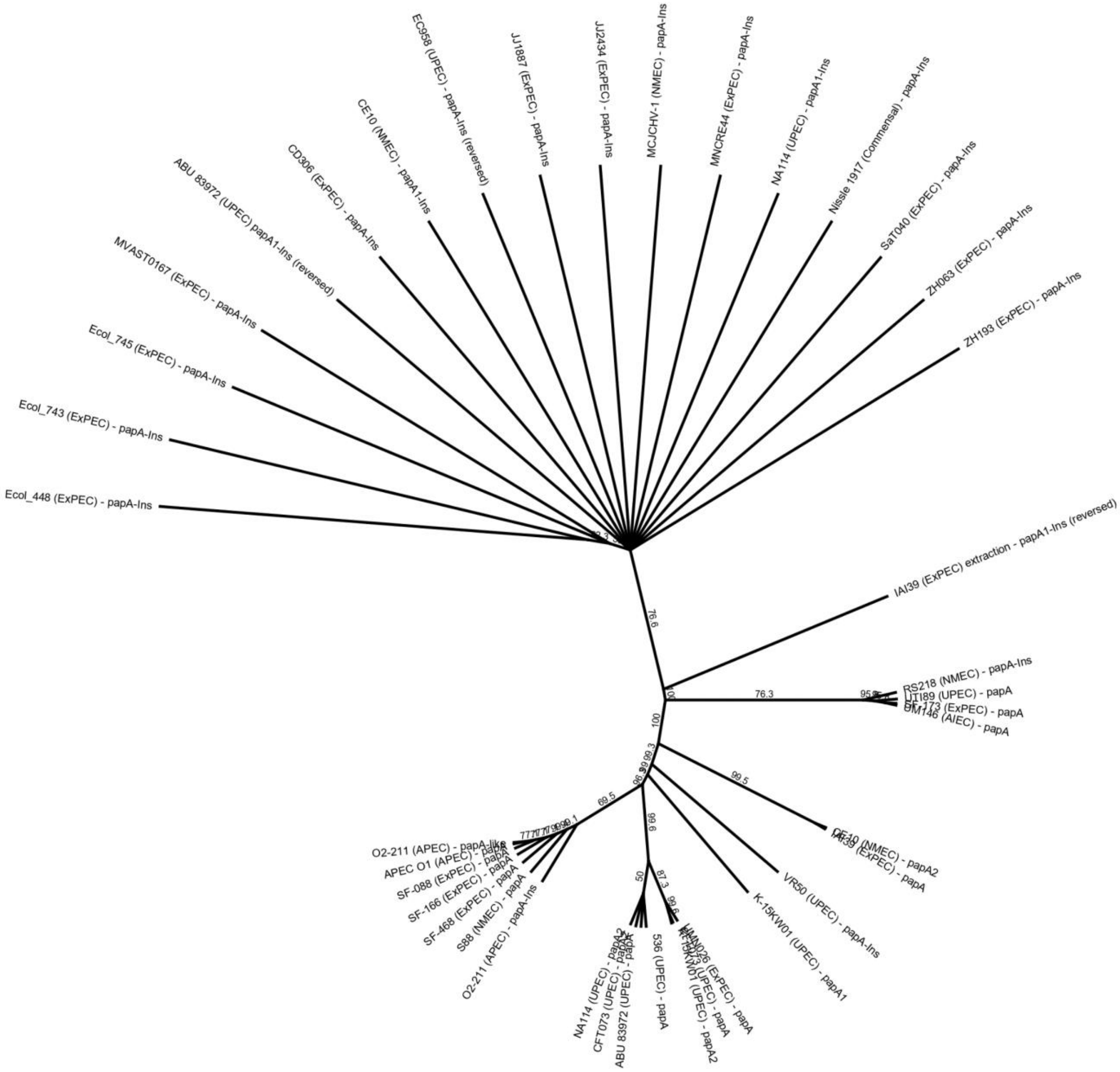

Supporting Figure 4: *papA* is Susceptible to Transposon Insertion. Unrooted tree shows relatedness of *papA* from all *papA* carrying strains from our pathotype database. "*papA-Ins*" indicates instances where *papA* was interrupted by transposon insertion. Results suggest that insertion of a transposon likely happened more than once: see O2-211, VR50, RS218, and IAI39. Branches labeled with percent consensus support. Nucleotide alignment created using Geneious Alignment. Tree created using Geneious Tree maker using Jukes-Cantor genetic distance model with neighbor-joining method and bootstrap support from 1,000 replicates.

Supporting Figure 5

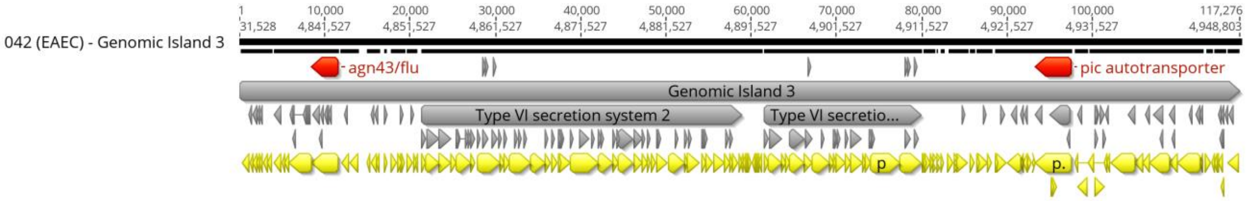

Supporting Figure 5: Genomic island 3 from *E. coli* 042, an EAEC strain from phylogroup D.

Supporting Figure 6

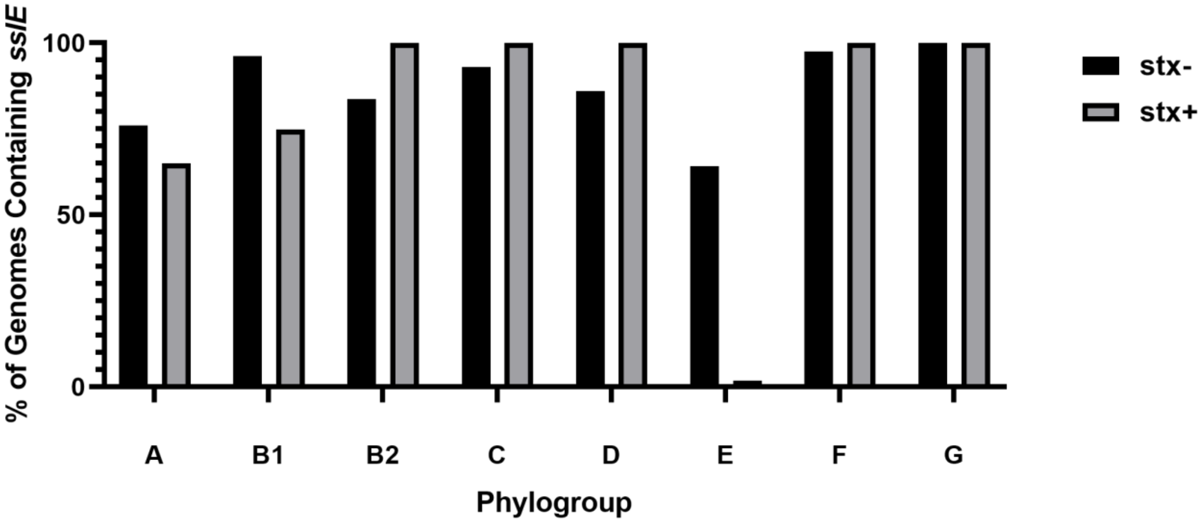

Supporting Figure 6: Phylogroup Distribution of *sslE* in *stx+* and *stx-* Strains. Strains from each phylogroup were divided based on hits vs no hits of Shiga Toxin (*stx1* or *stx2*). These strains were then blasted against *sslE* reference and the % of those genomes containing *sslE* were reported.

Supporting Figure 7

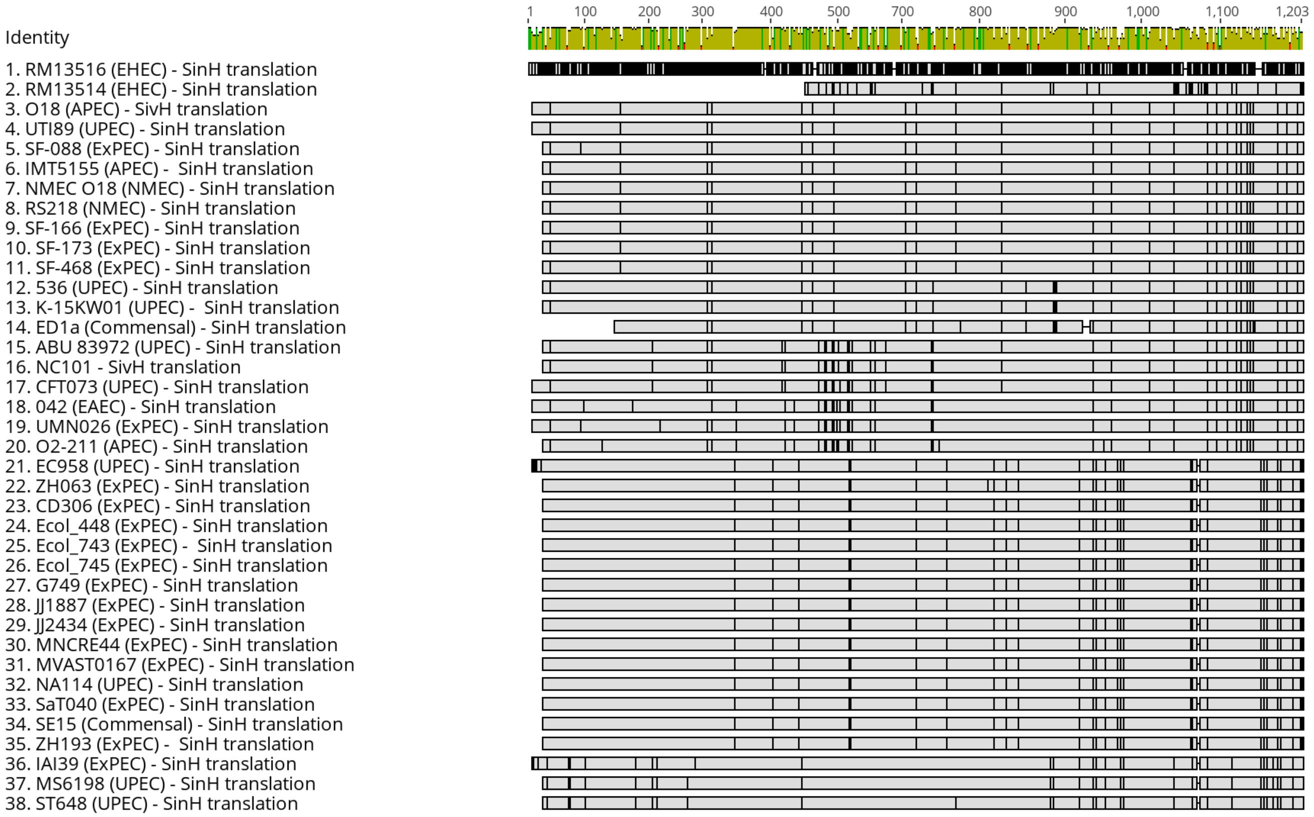

Supporting Figure 7: Amino Acid Alignment of SinH. This alignment shows every SinH protein in our phylogroup database. Black marks indicate disagreements from majority consensus. Identity histogram can be found at the top of the graph. Alignment created using Geneious Alignment.

Supporting Figure 8

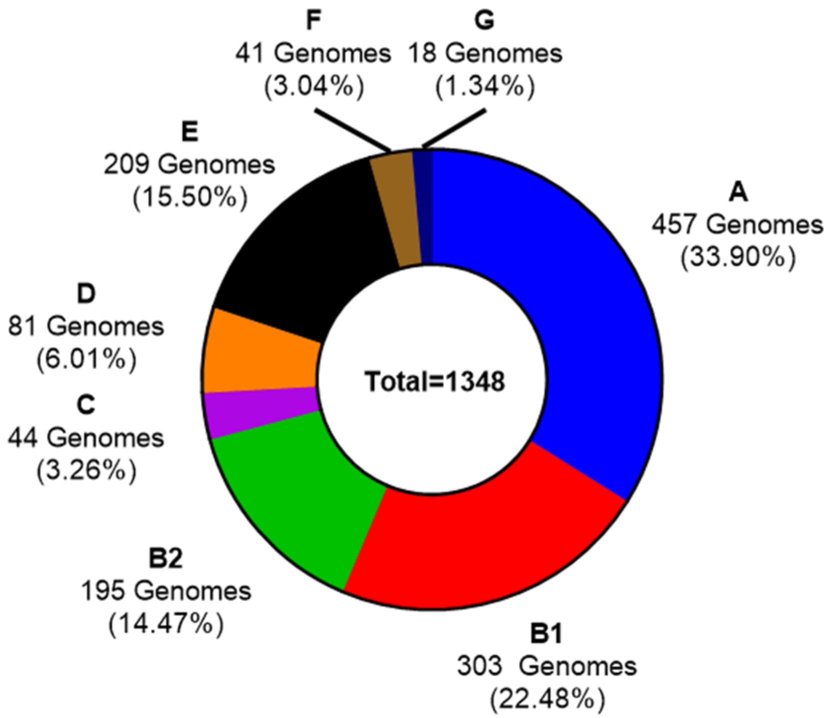

Supporting Figure 8: Phylogroup Breakdown of Phylogroup Database.

Supporting Figure 9

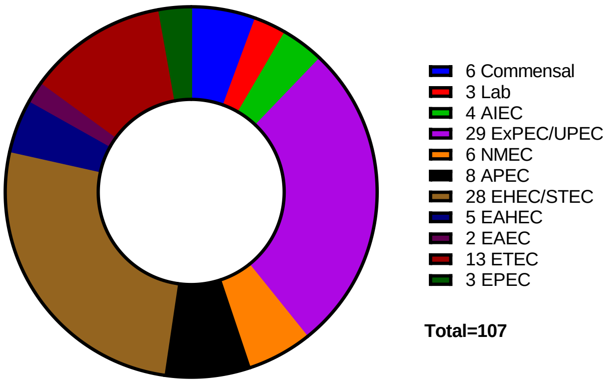

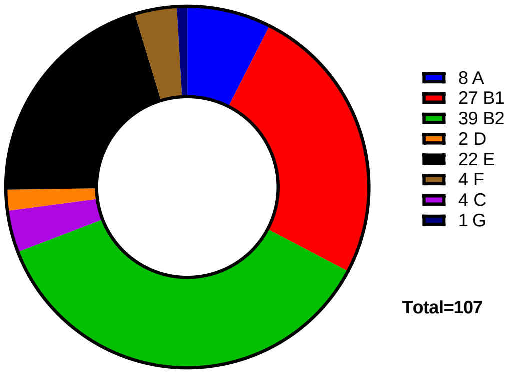

Supporting Figure 9: Pathotype and Phylogroup Breakdown of Pathotype Database.

References

1. Clermont O, Christenson JK, Denamur E, Gordon DM. The Clermont Escherichia coli phylo-typing method revisited: improvement of specificity and detection of new phylo-groups. Environ Microbiol Rep. 2013;5: 58–65. doi:10.1111/1758-2229.12019

2. Lescat M, Clermont O, Woerther PL, Glodt J, Dion S, Skurnik D, et al. Commensal Escherichia coli strains in Guiana reveal a high genetic diversity with host-dependant population structure. Environ Microbiol Rep. 2013;5: 49–57. doi:10.1111/j.1758-2229.2012.00374.x

3. Clermont O, Dixit OVA, Vangchhia B, Condamine B, Dion S, Bridier-Nahmias A, et al. Characterization and rapid identification of phylogroup G in Escherichia coli , a lineage with high virulence and antibiotic resistance potential. Environ Microbiol. 2019;21: 3107–3117. doi:10.1111/1462-2920.14713

4. Waters NR, Abram F, Brennan F, Holmes A, Pritchard L. Easy phylotyping of Escherichia coli via the EzClermont web app and command-line tool. Access Microbiol. 2020;2. doi:10.1099/acmi.0.000143

5. Beghain J, Bridier-Nahmias A, Le Nagard H, Denamur E, Clermont O. ClermonTyping: an easy-to-use and accurate in silico method for Escherichia genus strain phylotyping. Microb Genomics. 2018;4. doi:10.1099/mgen.0.000192

6. Cooper KK, Mandrell RE, Louie JW, Korlach J, Clark TA, Parker CT, et al. Comparative genomics of enterohemorrhagic Escherichia coli O145:H28 demonstrates a common evolutionary lineage with Escherichia coli O157:H7. BMC Genomics. 2014;15: 17. doi:10.1186/1471-2164-15-17

7. Willis LM, Whitfield C. KpsC and KpsS are retaining 3-deoxy-D-manno-oct-2-ulosonic acid (Kdo) transferases involved in synthesis of bacterial capsules. Proc Natl Acad Sci. 2013;110: 20753–20758. doi:10.1073/pnas.1312637110

8. Sugai T, Lin C-H, Shen G-J, Wong C-H. CMP-KDO synthetase: Overproduction and application to the synthesis of CMP-KDO and analogs. Bioorg Med Chem. 1995;3: 313–320. doi:10.1016/0968-0896(95)00023-A
